# Supplementary material for: Bispecific GLP-1/GLP-2 agonism in advanced type 2 diabetes: preclinical characterization and a randomized, double-blind, placebo-controlled phase I trial
Source: Nat Commun. 2026 Mar 24;17:4477. doi: 10.1038/s41467-026-71080-0 (PMC13187202; doi:10.1038/s41467-026-71080-0)
Supplement: Supplementary file 1 — Supplementary Information [file 41467_2026_71080_MOESM1_ESM.pdf]

Supplementary Information

Bispecific GLP-1/GLP-2 Agonism in Advanced Type 2 Diabetes: Preclinical Characterization and a Randomized, Double-blind, Placebo-controlled Phase I Trial

Sang-In Yang, Sae Won Kim, Kyung-Hwa Son, Seung-Ah Lee, Jong-Gyun Kim, Jae-Il Roh, and Young Chul Sung

Supplementary Methods

Comparison of PG-105 and PG-102. Eight-week-old male C57BL/6J mice (Koatech, Korea; n = 4 per group) were maintained at 21 ± 2 °C and 50 ± 10% relative humidity on a 12-h light/dark cycle, with ad libitum access to food and water. PG-102 or PG-105 was administered subcutaneously at 30 nmol/kg every other day (Q2D) for 2 weeks. All procedures were approved by the Genenbio Institutional Animal Care and Use Committee (IACUC No. R20-09-09). Comparative assessments included length and weight of the small and large intestines, serum endotoxin (LPS) concentration, and gallbladder volume.

***In vitro* bioactivity assay for receptor selectivity.** To evaluate off-target agonism, receptor activity was measured as cAMP accumulation in CHO-K1 cells stably expressing human GIPR or GCGR (Eurofins DiscoverX Ltd., Shanghai, China). Cells were maintained and assayed according to the manufacturers' instructions. On the assay day, cells were exposed to serial dilutions of PG-102, and cAMP responses were quantified using the vendor-supplied detection protocol. Parallel wells received positive controls: GIP and tirzepatide for GIPR, and glucagon and retatrutide for GCGR. Concentration–response curves were fitted with a four-parameter logistic model (GraphPad Prism), and responses were normalized to the mean maximal signal of the respective native ligand run on the same plate. Lack of detectable activity was concluded

when PG-102 responses were indistinguishable from vehicle across the concentration range.

Immunohistochemistry for CD45. Pancreatic tissues were harvested from the advanced type 2 diabetes (T2D) mouse model described in the main text, fixed in 10% neutral-buffered formalin, embedded in paraffin, and sectioned for histological evaluation. Immunohistochemical staining was performed using an anti-CD45 antibody (Invitrogen (Carlsbad, CA, USA), 11-0451-82; 1: 300; rat). Images were acquired using an ECLIPSE Ci-L microscope (Nikon Instruments Inc., Melville, NY, USA), and CD45-positive areas were quantified with ImageJ software.

***In vivo* study: metabolic assessments, pancreatic histology and immunofluorescence.** Fourteen-week-old male *db/db* mice (BKS-Leprdb/db/JOriRj) with moderate hyperglycemia (baseline HbA1c 9–10%) were allocated into three groups (vehicle, tirzepatide, PG-102; n = 6 per group), ensuring between-group balance with respect to baseline glucose, HbA1c, and body weight. Mice were housed at 21 ± 2 °C and 50 ± 10% relative humidity on a 12-h light/dark cycle with ad libitum access to food and water. PG-102 (30 nmol/kg) or tirzepatide (30 nmol/kg) was administered subcutaneously every three days for 12 weeks. Vehicle controls received matched volumes of formulation buffer on the same schedule. For metabolic assessments, non-fasting blood glucose was measured from tail vein blood at weekly intervals using a handheld glucometer. HbA1c was measured every 4 weeks using a validated immunoassay according to the manufacturer's instructions. Body weight was recorded weekly throughout the study. At the end of study (Week 26), pancreatic tissues were harvested, fixed in 10% neutral-buffered formalin, embedded in paraffin, and sectioned at 4 µm. Sections were subjected to antigen retrieval, blocked, and incubated with primary antibodies against insulin (Abcam, ab181547; 1:10,000; rabbit) and glucagon (Abcam, ab10988; 1:200; mouse), followed by appropriate fluorescent secondary antibodies and nuclear counterstaining with DAPI (Abcam, Cambridge,

UK; Santa Cruz Biotechnology, Dallas, TX, USA). Images were acquired using an ECLIPSE Ci-L microscope (Nikon Instruments Inc., Melville, NY, USA) under identical exposure settings across groups. For each mouse, 5–10 non-overlapping islets were analyzed. Total islet area was delineated manually, and insulin$^+$ and glucagon$^+$ areas were quantified by threshold-based segmentation in ImageJ. β-cell area and α-cell area were expressed as insulin$^+$ or glucagon$^+$ area normalized to total islet area (%) for each field. All animal procedures were approved by the GI Biome Institutional Animal Care and Use Committee (GIB-23-02-007) and conducted in accordance with the Guide for the Care and Use of Laboratory Animals.

Cell culture and gene expression. INS-1 β cells and 3T3-L1 preadipocytes were maintained in DMEM (Thermo Fisher Scientific) with 10% FBS and 1% penicillin–streptomycin at 37 °C, 5% CO_2; L6-GLUT4myc and C2C12 myoblasts were maintained in α-MEM (Thermo Fisher Scientific) with the same supplements. 3T3-L1 differentiation was induced at confluence (day 0) with DMEM containing 0.5 mM IBMX, 1 μM dexamethasone, and 10 μg/mL insulin; on day 2 the medium was changed to DMEM with 10% FBS and 10 μg/mL insulin and refreshed every 2 days. Differentiation was complete by day 6–8 (lipid droplets visible). L6-GLUT4myc and C2C12 differentiation was initiated at 80–90% confluence using α-MEM with 2% horse serum, replaced every 2–3 days until multinucleated myotubes formed (5–7 days). Total RNA was extracted with TRIzol reagent (Life Technologies, Waltham, MA, USA). cDNA was synthesized from 1 μg total RNA using SuperScript II Reverse Transcriptase (Promega, Madison, WI, USA) according to the manufacturer's instructions. RT-PCR was performed to amplify each cDNA using the AccuPower PCR premix (Promega). Quantitative RT-PCR was performed using SYBR Premix ExTaq (Takara, Shiga, Japan) and the ABI 7500 real-time PCR system (Applied Biosystems, Foster City, CA). Each cycle threshold (Ct) values was subtracted from the glyceraldehyde 3-phosphate dehydrogenase (Gapdh) Ct value of the same samples

(dCt) and then subtracted from the dCt value of each control set (ddCt). Relative mRNA levels were expressed as 2-ddCt.

Primer sequences. Quantitative PCR primers used in this study were selected based on previously published sequences and synthesized by Cosmo Genetech (Seoul, Republic of Korea). Primer sequences (5'–3') are listed below. All primers were validated for specificity prior to use. The primer sequences generated and used in this study are available for academic reuse upon reasonable request. Requests for materials should be directed to the corresponding author.

Gene	Forward primer (5'→3')	Reverse primer (5'→3')
Mafa	GCT TCA GCA AGG AGG AGG TCA T	TCT CGC TCT CCA GAA TGT GCC G
Pdx1	GGA TGA AAT CCA CCA AAG CTC AC	AGC ATC ACT GCC AGC TCC A
Neurod1	CCC TAA CTG ATT GCA CCA GC	TGC AGG GTA GTG CAT GGT AA
Tnfa	GCC TCT TCT CAT TCC TGC TT	TGG GAA CTT CTC ATC CCT TTG
Atrogin-1	CTT CTC GAC TGC CAT CCT GGA T	TCT TTT GGG CGA TGC CAC TCA G
MuRF-1	TAC CAA GCC TGT GGT CAT CCT G	ACG GAA ACG ACC TCC AGA CAT G
Gapdh	GCT GGT CAT CAA CGG GAA A	ACG CCA GTA GAC TCC ACG ACA

Flow cytometry. GLP-1 and GLP-2 receptor expression on pancreatic INS-1 β-cells, differentiated 3T3-L1 adipocytes and L6-GLUT4myc myotubes was analyzed via flow cytometry. Cells were harvested, washed with PBS containing 2% FBS, and resuspended in FACS buffer (PBS with 2% FBS). Primary antibodies against GLP-1R (FITC-conjugated; AGR-021-F, Alomone Labs, Jerusalem, Israel; 1:50) and GLP-2R (AGR-022, Alomone Labs, Jerusalem, Israel; 1:50) were applied for 30 min at 4°C in the dark. After washing, cells were incubated with fluorophore-conjugated secondary antibodies for 30 min at 4°C. Appropriate controls, including unstained, single-stained, and isotype controls, were used for gating and background correction. Data acquisition was performed on a CytoFLEX LX flow cytometer (Beckman Coulter, Brea, CA, USA), and fluorescence intensity was analyzed using FlowJo software (TreeStar, Ashland, OR, USA). Receptor expression was quantified as mean

fluorescence intensity (MFI) relative to isotype controls.

Glucose uptake assay using antagonists. Glucose uptake in differentiated 3T3-L1 adipocytes and L6-GLUT4myc myotubes was measured using a glucose uptake cell-based assay kit (Cayman, Ann Arbor, MI, USA), according to the manufacturer's protocol. After 2 h of serum starvation, cells were pre-treated for 30 min with 10 nM Exendin (9-39) (GLP-1 receptor antagonist; Angene Chemical, Nanjing, China) or GLP-2 (3-33) (GLP-2 receptor antagonist; R&D system, Minneapolis, MN, USA) prior to co-treatment with the indicated drugs (300 nM) and insulin for 10 min. Cells were washed three times with Krebs buffer (20 mM HEPES, 5 mM KH_2PO_4, 1 mM $MgSO_4$, 1 mM $CaCl_2$, 136 mM NaCl, 4.7 mM KCl, pH 7.4), then incubated with 500 µg/ml 2-NBDG, a fluorescence-labelled deoxyglucose analog, for 20 min at room temperature. Reactions were terminated by washing with ice-cold Krebs buffer. Cells were lysed using Mammalian Protein Extraction Reagent Thermo Fisher Scientific) and centrifuged at 15000 × g for 10 sec. After removing the supernatant, assay buffer added, and 2-NBDG fluorescence was measured using a microplate reader (excitation/emission = 485/535 nm). Glucose uptake was normalized to protein content quantified using a bicinchoninic acid (BCA) assay (Thermo Fisher Scientific).

Receptor trafficking analysis. HEK293 cells co-expressing human GLP-1R and GLP-2R were seeded on attachment factor (AF)-coated coverslips in 24-well plates (5×10^5 cells per well) and cultured for 2 days. Cells were treated with the indicated ligands and fixed with 4% paraformaldehyde (PFA) for 15 minutes at room temperature, followed by permeabilization with 0.1% Triton X-100 for 5–10 minutes and blocking with 5% bovine serum albumin (BSA) for 60 minutes. Fluorophore-conjugated primary antibodies were applied overnight at 4 °C: phycoerythrin (PE)-conjugated anti-human GLP-1R (FAB2814P, R&D Systems; 1:100) and Alexa Fluor 488-conjugated anti-human GLP-2R (FAB4285G, R&D Systems; 1:100). Nuclei

were counterstained with 4′,6-diamidino-2-phenylindole (DAPI) (ab104139, Abcam; 1 μg/mL). Coverslips were mounted with fluorescence mounting medium and imaged using a Zeiss LSM 880 confocal microscope. Confocal images were processed with ImageJ (v1.54x). Internalized receptor puncta were identified by automated thresholding with size filtering, quantified per cell, normalized to cell area, and reported as puncta density (puncta·μm^{-2}). Time-course data are presented as internalized GLP-1R or GLP-2R puncta per area.

26-week GLP repeat-dose toxicology study in Sprague–Dawley rats. A GLP-compliant 26-week repeat-dose toxicity study was conducted in male and female Sprague–Dawley rats at Ina Research Inc. (Japan) in accordance with OECD GLP principles and approved by the institutional IACUC (protocol ZO23241). Rats received PG-102 subcutaneously once weekly at 4, 10, or 20 mg/kg for 26 consecutive weeks. Animals were housed under controlled environmental conditions with ad libitum access to standard diet and water and were monitored at least twice daily throughout the study. Clinical observations included general condition and body weight. Humane endpoints were predefined and included sustained body weight loss, persistent anorexia, or signs of systemic distress; no unscheduled euthanasia related to study drug occurred. At scheduled termination, animals were euthanized under anesthesia and subjected to complete gross necropsy. Organs and tissues were processed for histopathological evaluation as summarized in Supplementary Table 1.

Exploratory exposure–response analysis. An exploratory exposure–response analysis was performed by plotting individual PK exposure ($AUC_{0\text{-}t}$) against OGTT glucose $AUC_{0–2h}$. Linear regression with 95% confidence intervals was applied across all active treatment cohorts. Pearson and Spearman correlation analyses were conducted to evaluate the association. Analyses were performed using GraphPad Prism (version 9.0, GraphPad Software, San Diego, CA, USA).

Exploratory analyses of body weight, composition, glycemic, and inflammatory biomarkers. Exploratory endpoints included changes in body weight, percent weight change, BMI, waist circumference, waist–hip ratio (WHR), body composition (fat mass and lean mass by dual-energy X-ray absorptiometry [DEXA]), glycated hemoglobin (HbA1c), fasting plasma glucose (FPG), and high-sensitivity C-reactive protein (hsCRP). Body weight and composition were assessed at Day 36 (D36, one week after the last administration), with the exception of DEXA scans, which were performed at Day 30 (D30, one days after the final dose). Glycemic and inflammatory biomarkers were measured at Day 28 (D28, after four weekly doses, prior to the fifth administration) and Day 57 (D57, four weeks after the last administration). All exploratory endpoints were summarized using descriptive statistics (mean ± SD). Change-from-baseline values were derived as post-treatment minus baseline. No formal hypothesis testing was performed, as these analyses were exploratory and not powered for statistical inference.

Statistical analysis. Data are expressed as mean ± standard error of the mean (SEM). Statistical analyses were performed using GraphPad Prism (version 9.0, GraphPad Software, San Diego, CA, USA). For comparisons among multiple groups, one-way ANOVA followed by Tukey's post hoc test was used to assess all possible pairwise differences. For time-course experiments, two-way ANOVA with Tukey's post hoc test was conducted. For analyses requiring comparisons against PG-102, Dunnett's multiple comparisons test was applied with PG-102 as the reference group.

Clinical Supplementary Results

Exploratory exposure–response relationship between PK exposure and OGTT glucose excursion

Exploratory analyses were conducted to examine the relationship between systemic PK exposure and OGTT glucose excursion. Across active treatment cohorts (15 mg: n = 5; 30 mg: n = 6; 30/60 mg: n = 6), a weak negative association between PK exposure and OGTT glucose $AUC_{0–2h}$ was observed. Linear regression yielded a slope of $−1.95 \times 10^{-3}$ (95% CI $−5.21 \times 10^{-3}$ to $+1.30 \times 10^{-3}$; $R^2 = 0.10$), with correlation analyses showing Pearson $r = −0.31$ ($p = 0.22$) and Spearman $\rho = −0.20$ ($p = 0.45$). Given the small sample size and low correlation coefficients, these findings should be interpreted with caution and are considered exploratory, without evidence sufficient to support a definitive exposure–response relationship.

Exploratory effects of PG-102 on body weight and body composition

Exploratory analyses of body weight and anthropometric measures demonstrated modest, dose-dependent reductions with PG-102 compared with placebo (Supplementary Table 5). Absolute and percent changes in body weight were −1.2 ± 1.5 kg (−1.5 ± 2.1%) in the placebo group versus −1.7 ± 1.8 to −3.0 ± 2.0 kg (−2.2 ± 2.3% to −3.5 ± 2.2%) across PG-102 groups. Corresponding decreases in BMI were −0.41 ± 0.53 kg/m² with placebo compared with −0.58 ± 0.62 to −0.94 ± 0.59 kg/m² with PG-102. Waist circumference declined by −1.8 ± 1.4 cm in the placebo group and by −2.3 ± 2.8 to −4.1±0.8 cm with PG-102. Changes in waist–hip ratio, fat mass, and lean mass were small and comparable to placebo. Collectively, these findings suggest that PG-102 was associated with numerically greater reductions in body weight and related measures than placebo, although changes were modest and consistent with the short

treatment duration and high baseline insulin sensitivity of the study population.

Exploratory effects of PG-102 on glycemic and inflammatory biomarker

Exploratory analyses of glycemic and inflammatory markers revealed minimal changes with PG-102 compared with placebo (Supplementary Table 6). HbA1c levels remained stable across all treatment groups, ranging from 5.1–5.5% at baseline to 5.1–5.4% post-treatment. FPG values fluctuated modestly, with placebo increasing from 87.7 ± 6.3 mg/dL to 93.3 ± 6.7 mg/dL at Day 28, while PG-102 groups showed small decreases or variable changes (range: −5.6 to + 2.7 mg/dL from baseline). hsCRP values were low at baseline (0.05–0.10 mg/dL) and exhibited no consistent treatment-related changes. These findings indicate that glycemic and inflammatory parameters were not meaningfully affected during the short treatment period in this healthy volunteer population.

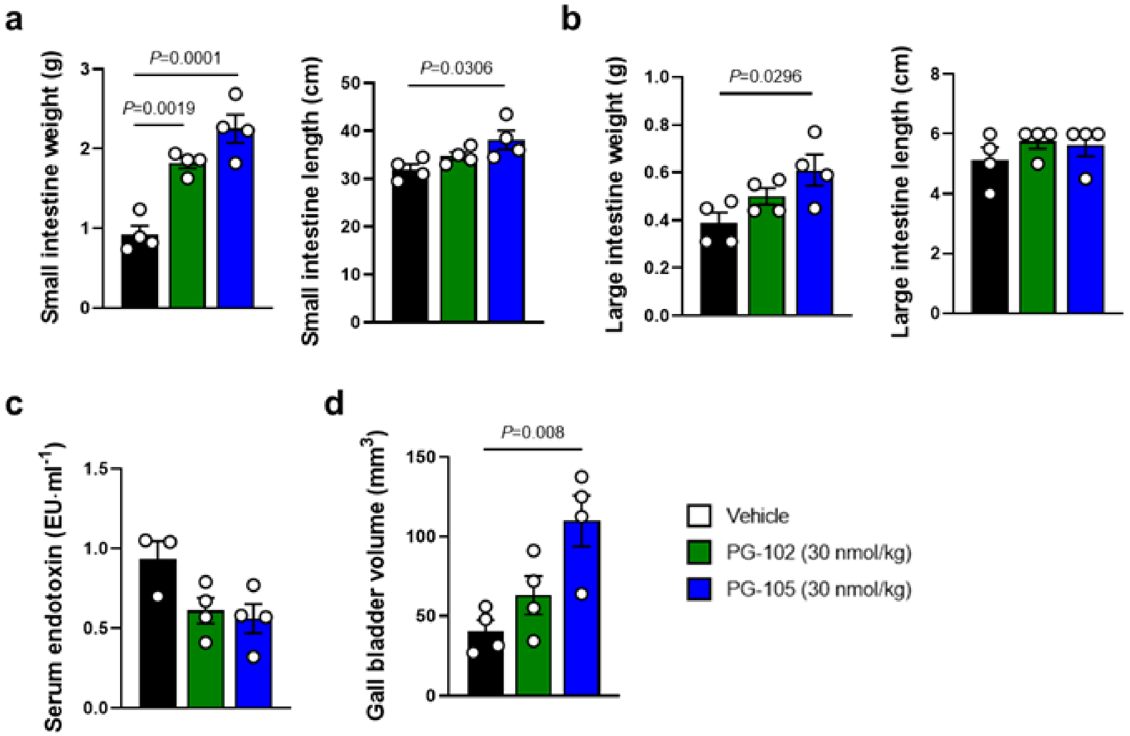

Supplementary Fig. 1. Comparative change of PG-105 and PG-102. Eight-week-old male C57BL/6J mice were treated subcutaneously every two days for 2 weeks with PG-105 (30 nmol/kg) or PG-102 (30 nmol/kg). Comparative evaluations were performed for intestinal weight and length change, including (**a**) small intestine and (**b**) large intestine. In addition, (**c**) serum endotoxin levels were assessed, and (**d**) gallbladder volume changes were compared. Data are presented as mean ± SEM (standard error of the mean) with individual data points overlaid (n = 4 mice per group). Statistical significance was determined by one-way ANOVA with Tukey's post hoc test. Source data are provided as a Source Data file.

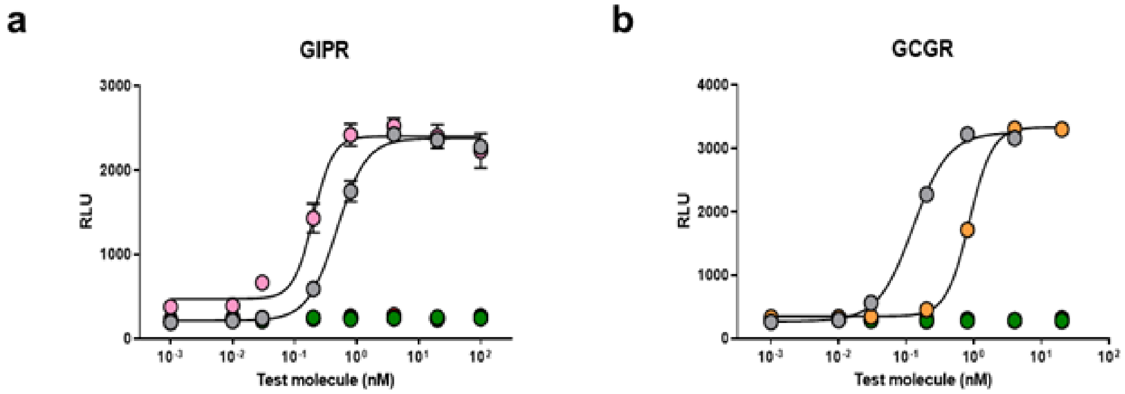

Test molecule	EC50 for agonist stimulation (nM)
GIP	0.48
Tirzepatide	0.20
GLP-1-Fc	-
GLP-2-Fc	-
PG-102	-

Test molecule	EC50 for agonist stimulation (nM)
GCG	0.13
Retatrutide	0.85
GLP-1-Fc	-
GLP-2-Fc	-
PG-102	-

Supplementary Fig. 2. *In vitro* assessment of PG-102 activity on other class B GPCRs (GIPR and GCGR). CHO-K1 cell lines stably expressing (**a**) GIPR or (**b**) GCGR (Eurofins DiscoverX Ltd., Shanghai, China) were used to assess receptor activity. GIP and tirzepatide were included as positive controls for GIPR activation, while glucagon and retatrutide served as positive controls for GCGR activation. GLP-1-Fc and GLP-2-Fc were also tested for comparison. Data are presented as mean ± SEM (standard error of the mean) with individual data points overlaid (n = 3 independent experiments). No statistical analysis was performed. Source data are provided as a Source Data file.

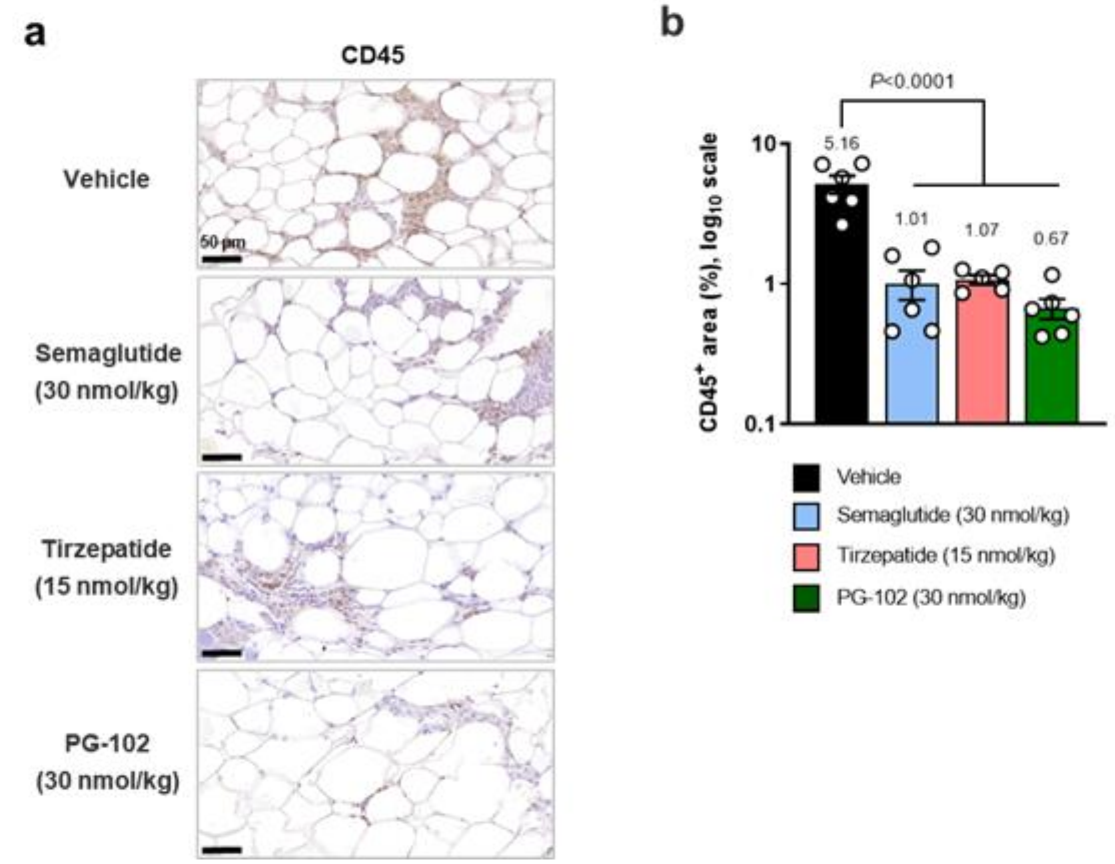

Supplementary Fig. 3. Peri-pancreatic fat inflammation analysis of PG-102 compared to semaglutide and tirzepatide in a *db/db* mouse model. (**a**) Representative immunohistochemistry (IHC) images for CD45 (pan-leukocyte marker) in peri-pancreatic adipose tissues of *db/db* mice treated semaglutide (30 nmol/kg), tirzepatide (15 nmol/kg), and PG-102 (30 nmol/kg). Scale bars, 50 µm. Representative images are shown from n = 6 mice per group with similar results. (**b**) Quantification of CD45+ area (% of tissue area). The y-axis is shown on a $\log_{10}$ scale. Data are presented as mean ± SEM (standard error of the mean) with individual data points overlaid (n = 6 mice per group). Statistical significance was determined by one-way ANOVA with Dunnett's multiple comparisons test. Source data are provided as a Source Data file.

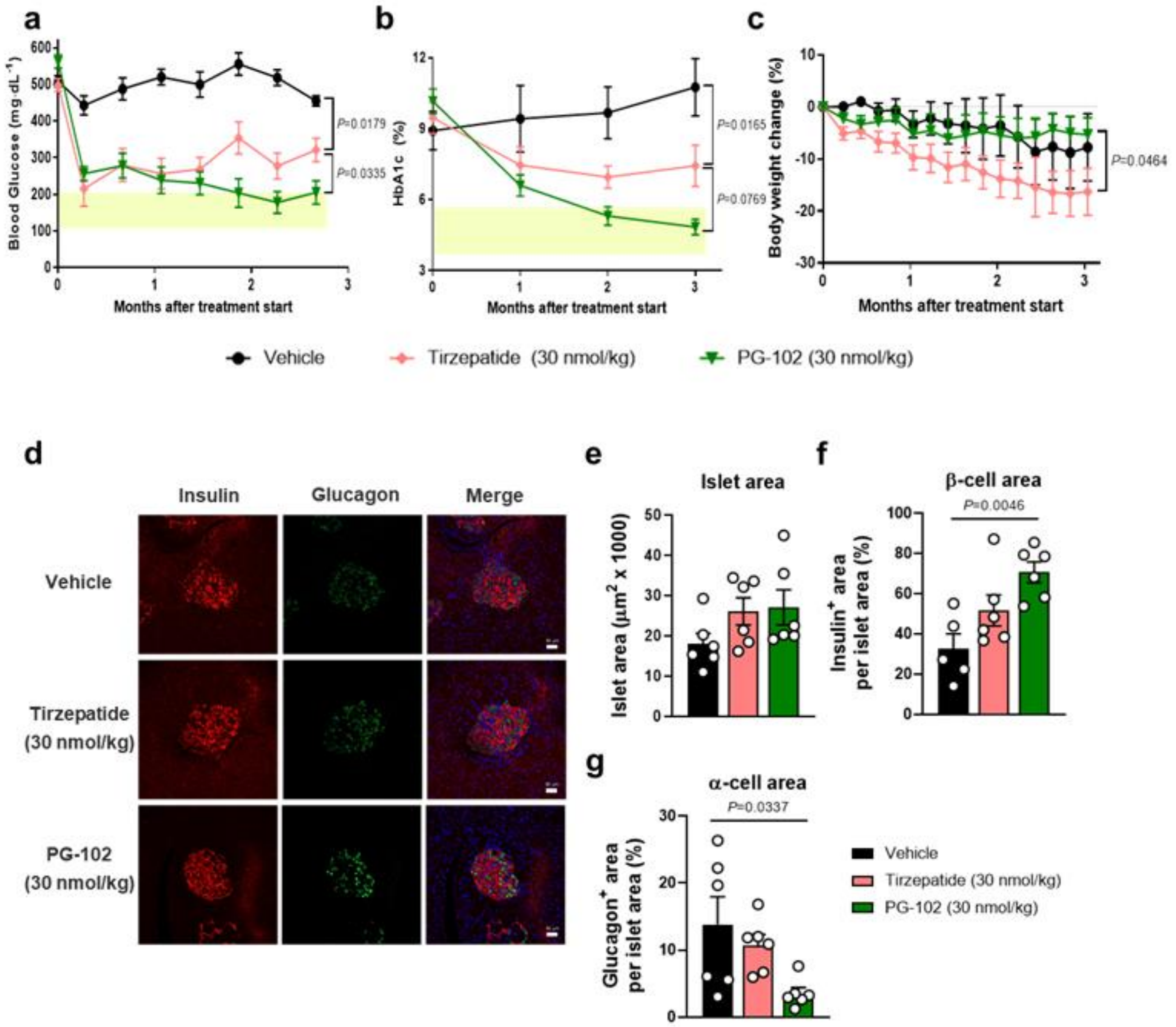

Supplementary Fig. 4. Comparative effects of PG-102 and tirzepatide in a moderate hyperglycemia *db/db* mouse model. Fourteen-week-old male *db/db* mice with moderate hyperglycemia (baseline HbA1c 9–10%) were treated subcutaneously every three days for 12 weeks with PG-102 (30 nmol/kg) or tirzepatide (30 nmol/kg). (**a–c**) Longitudinal assessment of non-fasting blood glucose, HbA1c, and body weight (vehicle, n = 6 mice; PG-102, n = 6 mice; tirzepatide, n = 7 mice). (**d**) Representative immunofluorescence staining of pancreatic islets for insulin (red) and glucagon (green). Representative images are shown from n = 6 mice per group with similar results. (**e–g**) Quantification of islet area, β-cell area (insulin^{+} area/islet area), and α-cell area (glucagon^{+} area/islet area). Data are presented as mean ± SEM (standard

error of the mean) with individual data points overlaid (n = 6 mice per group). Statistical significance was determined by two-way ANOVA for longitudinal analyses (**a–c**) or one-way ANOVA with Tukey's post hoc test for endpoint comparisons (**e–g**). Source data are provided as a Source Data file.

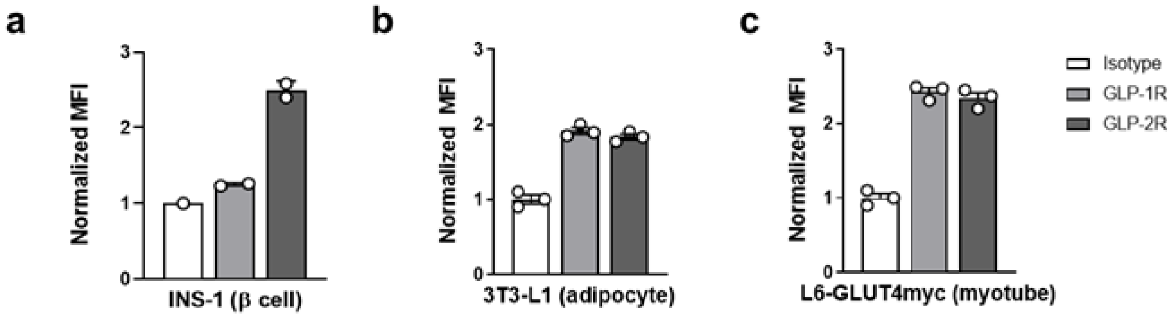

Supplementary Fig. 5. GLP-1R and GLP-2R expression on indicated cell lines. Surface expression of GLP-1 receptor (GLP-1R) and GLP-2 receptor (GLP-2R) on (**a**) INS-1 pancreatic β cells (n = 2 independent experiments), (**b**) differentiated 3T3-L1 adipocytes (n = 3 independent experiments), and (**c**) differentiated L6-GLUT4myc myotubes (n = 3 independent experiments), measured as normalized mean fluorescence intensity (MFI). Data are presented as mean ± SEM (standard error of the mean) with individual data points overlaid. No statistical analysis was performed. Source data are provided as a Source Data file.

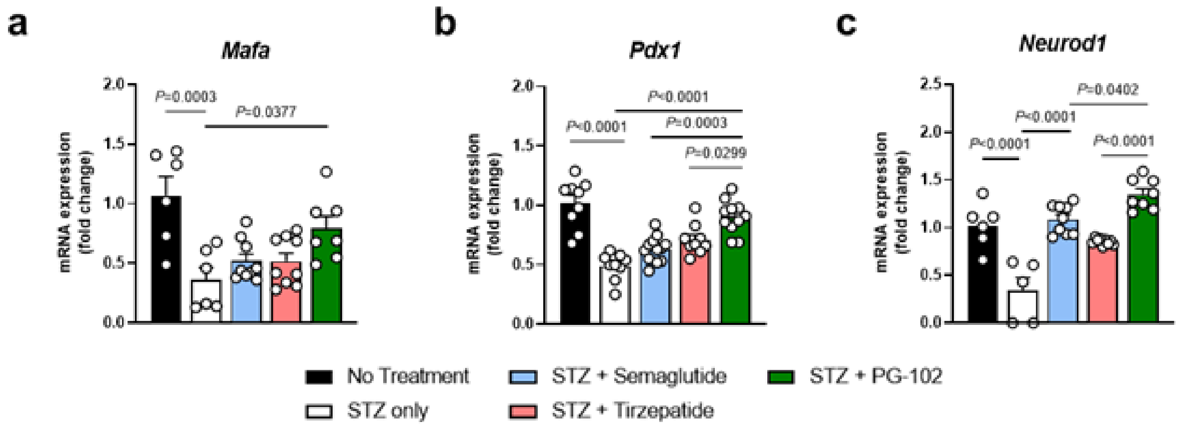

Supplementary Fig. 6. Cytoprotective effect of PG-102 in STZ-induced INS-1 pancreatic β cells. Cells were pretreated with 300 nM of the indicated agents for 24 h, followed by exposure to 10 nM STZ for 3 h to induce beta cell injury. Quantitative PCR analysis was performed to measure the expression of *Mafa* (**a**), *Pdx1* (**b**), and *Neurod1* (**c**) in STZ-induced INS-1 cells. Data are presented as mean ± SEM (standard error of the mean) with individual data points overlaid. *n* values represent independent cell culture wells and are provided for each group in the Source Data file. Statistical significance was determined by one-way ANOVA with Tukey's post hoc test (**a**–**c**). Source data are provided as a Source Data file.

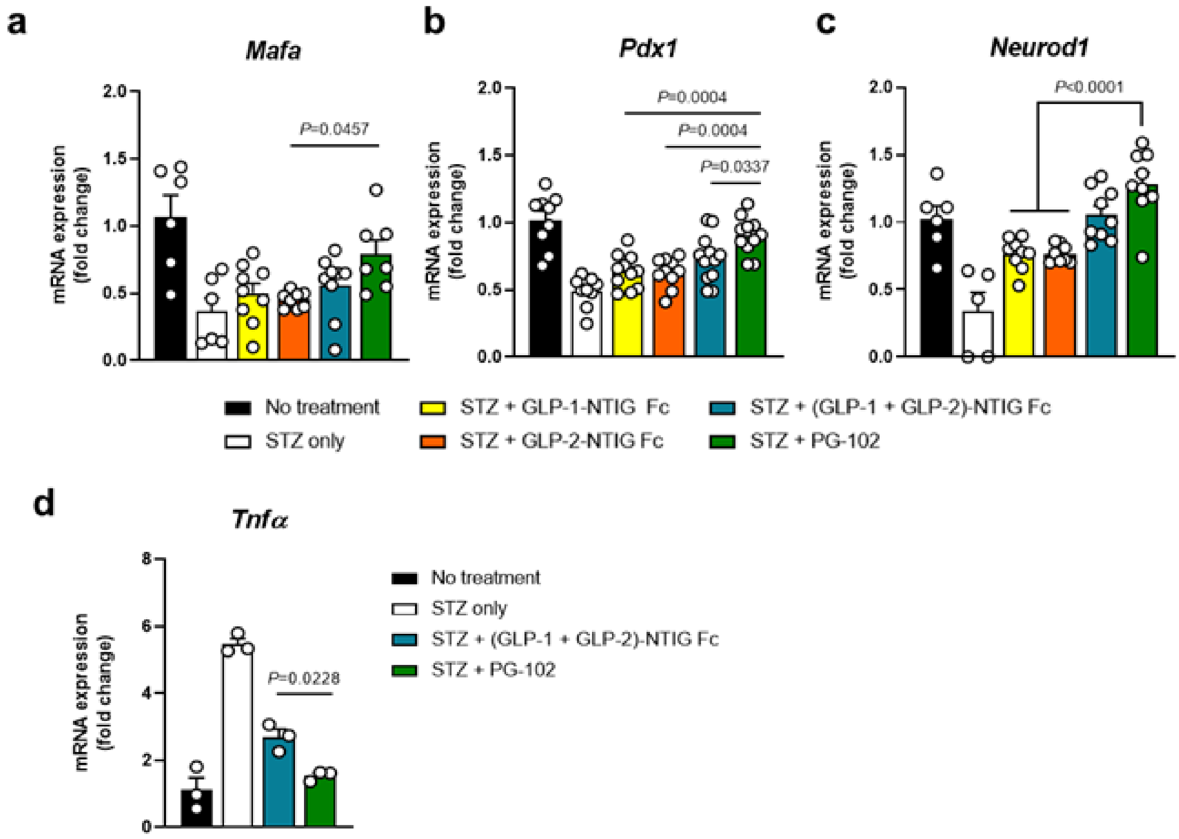

Supplementary Fig. 7. Dual GLP-1R/GLP-2R engagement by PG-102 in STZ-induced pancreatic INS-1 cells. INS-1 cell were pretreated for 24 h with GLP-1-NTIG Fc, GLP-2-NTIG Fc, their combination, or PG-102 (each 300 nM), then exposed to STZ (5 mM, 3 h). Gene expression of *Mafa* (**a**)*, Pdx1* (**b**), *Neurod1* (**c**), and *Tnfα* (**d**) was determined by qPCR, normalized to GAPDH, and expressed as fold-change over untreated. Data are presented as mean ± SEM (standard error of the mean) with individual data points overlaid. *n* values represent independent cell culture wells and vary across treatment groups and target genes; exact *n* values for each condition are provided in the Source Data file. Statistical significance was evaluated using one-way ANOVA with Dunnett’s multiple comparisons test. Source data are provided as a Source Data file.

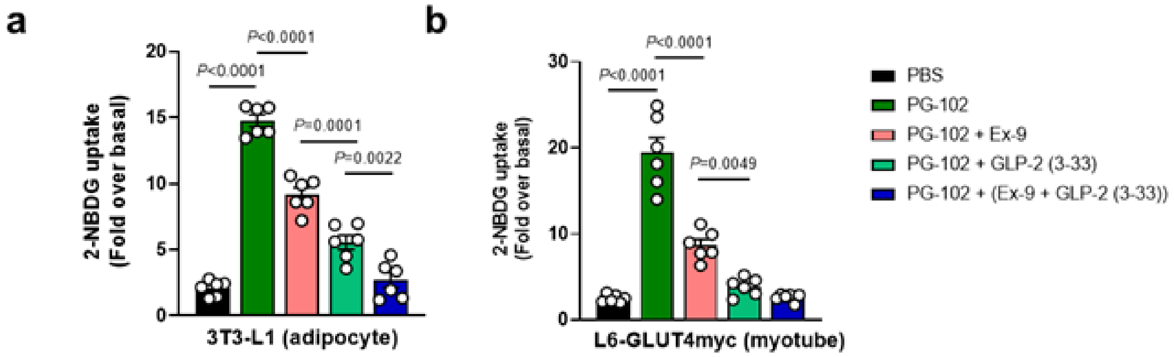

Supplementary Fig. 8. Mechanistic validation of glucose uptake with receptor antagonists. Insulin-stimulated glucose uptake (2-NBDG) was measured in differentiated 3T3-L1 adipocytes (**a**) and L6-GLUT4myc myotubes (**b**) following treatment with 300 nM of the indicated agents, 100 nM insulin, and/or 10 nM GLP-1 or GLP-2 receptor antagonists. Ex-9: exendin (9-39), GLP-1 antagonist corresponding to amino acids 9 through 39 of the full exending-4 peptide; GLP-2 (3-33): GLP-2 receptor antagonist corresponding to amino acids 3 through 33 of the full GLP-2 peptide. Data are presented as mean ± SEM (standard error of the mean) with individual data points overlaid (n = 6 independent experiments). Statistical significance was assessed using one-way ANOVA with Tukey's post hoc test. Source data are provided as a Source Data file.

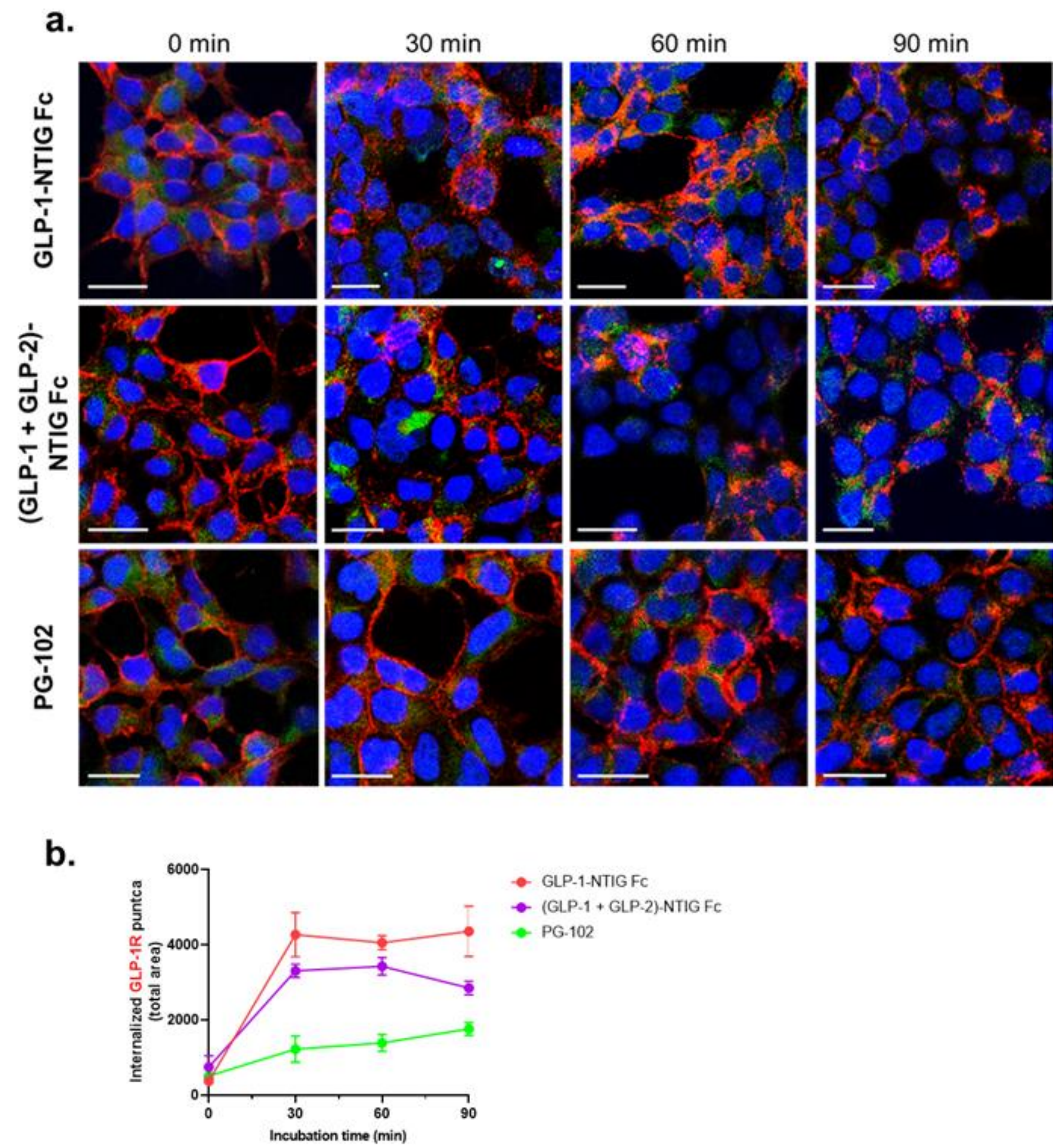

Supplementary Fig. 9. Receptor trafficking of PG-102 compared with monospecific and combination controls. (**a**) HEK293 cells co-expressing human GLP-1 receptor (GLP-1R) and human GLP-2 receptor (GLP-2R) were incubated with 200 nM of indicated agonists for 0-90 minutes. Receptors were visualized by confocal microscopy using PE-conjugated anti-GLP-1R antibody (red) and Alexa Fluor 488–conjugated anti-GLP-2R antibody (green); nuclei were counterstained with DAPI (blue). Internalized receptors appear as puncta within the cytoplasm.

Scale bars, 20 µm. Representative images are shown from n = 3 independent experiments with similar results. (**b**) Quantification of receptor internalization over time. Puncta were segmented by automated thresholding and size filtering in ImageJ, normalized to cell area, and expressed as puncta per μm^2. Data are mean ± SEM (standard error of the mean) with individual data points overlaid (n = 3 independent experiments). No statistical analysis was performed. Source data are provided as a Source Data file.

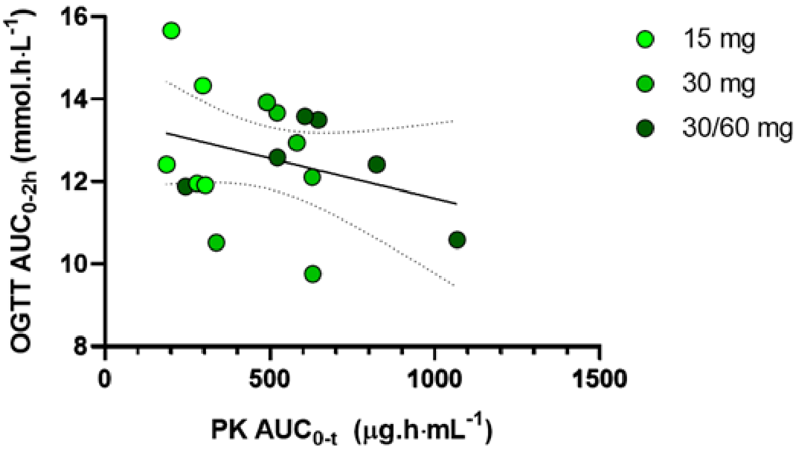

Supplementary Fig. 10. Exploratory exposure–response relationship between pharmacokinetic (PK) exposure and glucose excursion during oral glucose tolerance testing (OGTT) in the phase 1 multiple ascending dose (MAD) study. Scatter plot of PK exposure ($AUC_{0\text{-}t}$) versus OGTT glucose $AUC_{0–2h}$ in participants administered PG-102 (15 mg: n = 5, 30 mg: n = 6, 30/60 mg: n = 6). Each point represents an individual participant, with symbols/colors distinguishing dose groups. The solid line depicts the linear regression fit across all active cohorts and the dotted lines represent the 95% confidence interval. Source data are provided as a Source Data file.

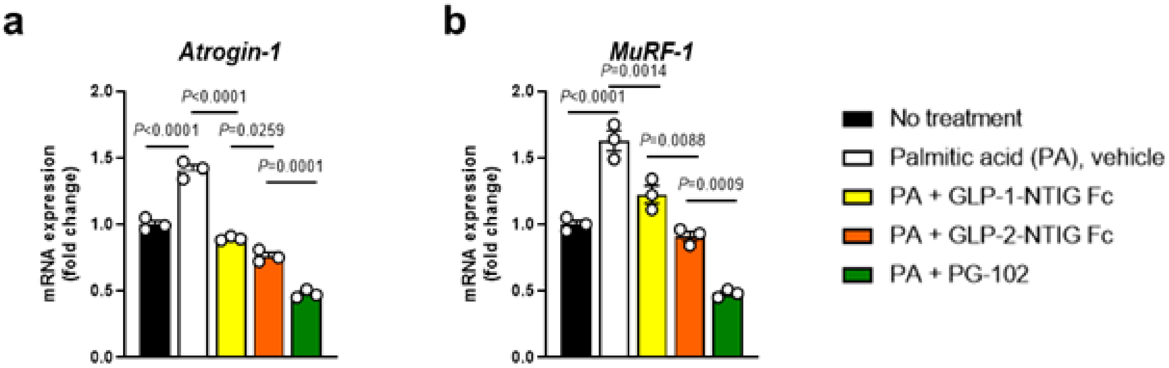

Supplementary Fig. 11. PG-102 attenuates palmitic acid–induced atrophy markers in C2C12 myotubes. C2C12 myoblasts were differentiated into myotubes in medium containing 2% horse serum (HS) and then exposed to palmitic acid (PA, 0.5 mM, 24 h) with the indicated agents (each 300 nM). mRNA expression of the atrophy-related genes *Atrogin-1* (**a**) and *MuRF-1* (**b**) was quantified by qPCR. Data are presented as mean ± SEM (standard error of the mean) with individual data points overlaid (n = 3 independent experiments). Statistical significance was assessed by one-way ANOVA with Tukey's post-hoc test. Source data are provided as a Source Data file.

a

Class	Drug	Species (Duration)	Dosing Interval	Documents (Study Number) Dose	Hyperplasia finding dose
GLP-1	Semaglutide	CD-1 mouse (13 weeks)	Daily	NDA 20637 (Study 200663) 1, 3, or 10 mg/kg/day	≥ 1 mg/kg/day
	Dulaglutide	SD rat (6 months)	Twice weekly	BLA 125469 (Study 7608-236) 1.63, 4.89 or 16.29 mg/kg/dose	Not observed
GLP-2	Teduglutide	CD-1 mouse (26 weeks)	Twice daily	NDA 203441 (Study 7203-112) 1, 5, or 25 mg/kg/dose	Not observed
GLP-1/GIP	Tirzepatide	SD rat (6 weeks)	Twice weekly	NDA 215866 0.5, 1.5, or 3 mg/kg/dose	3 mg/kg/dose
GLP-1/GLP-2	PG-102	SD rat (26 weeks)	Weekly	ZO23241 4, 10, or 20 mg/kg/dose	Not observed

b

Class	Drug	Species (Duration)	Dosing Interval	Documents (Study Number) Dose	Hyperplasia finding dose
GLP-1	Semaglutide	Cynomolgus monkey (52 weeks)	Twice weekly	NDA 209637 (Study JLY0140) 0.01, 0.06, or 0.36 mg/kg/day	Not observed
	Dulaglutide	Cynomolgus monkey (9 months)	Twice weekly	BLA 125469 (Study 7608-235) 0.41, 1.63, or 8.15 mg/kg/dose	Not observed
GLP-2	Teduglutide	Cynomolgus monkey (13 weeks)	Twice daily	NDA 203441 (Study 2303-100) 0.5, 2.5, or 12.5 mg/kg/dose	≥ 0.5 mg/kg/day
GLP-1/GIP	Tirzepatide	Cynomolgus monkey (6 months)	Weekly	NDA 215866 0.05, 0.15, or 0.5 mg/kg/dose	Not observed
GLP-1/GLP-2	PG-102	Cynomolgus monkey (26 weeks)	Weekly	ZO23242 2, 5, or 10 mg/kg/dose	Not observed

Supplementary Table 1. Thyroid C cell and gastrointestinal hyperplasia in long-term repeated-dose toxicity studies in rats and cynomolgus monkeys. Data are compiled from FDA toxicological documents describing chronic toxicity studies in rats (**a**) and cynomolgus monkeys (**b**), including study duration, dosing frequency, and dose levels at which thyroid C cell or gastrointestinal hyperplasia was observed. PG-102 did not induce thyroid C cell or gastrointestinal hyperplasia in 26-week repeated-dose toxicity studies in either species.

Descriptions	Cohort 1 (15mg)	Cohort 2 (30mg)	Cohort 3 (30/60mg)	Total
**Screening summary**				
Number of volunteers screened	17	19	31	67
Number of subjects enrolled†	8	8	8	24
Number of subjects NOT enrolled †	9	11	23	43
**Reason for Not enrolled**				
Eligibility criteria not met	7	9	20	36
Withdrew consent	1	0	0	1
Exceeded subject limit	1	2	3	6
**Subject allocation (PG-102: Placebo)**				
Enrolled	6:2	6:2	6:2	24
Treated	6:2	6:2	6:2	24
Non-treated	0:0	0:0	0:0	0
Completed study	5:2	6:2	6:2	23
Discontinued	1:0	0:0	0:0	1
**Reason for discontinuation**				
Withdrew consent	0	0	0	0
Serious protocol non-compliance	0	0	0	0
Investigator's decision related to an adverse event	1††	0	0	1
Others	0	0	0	0

†Enrolled = randomized; Not enrolled = screened but not randomized.
††One participant in the 15 mg cohort discontinued after the fourth dose due to an adverse event assessed as unrelated to the investigational product and was included in the safety analysis set only

Supplementary Table 2. Subject disposition across cohorts in the phase 1 multiple ascending dose (MAD) study. Summary of subject screening, enrollment, treatment allocation, and study completion for three PG-102 dose cohorts and the placebo group in the Phase 1 MAD study. Enrollment was defined as randomization; all enrolled participants were randomized within each cohort in a 6:2 ratio (PG-102: placebo). Data are presented for subjects enrolled, treated, and completed, as well as for dropouts with reasons specified (e.g., consent withdrawal, protocol non-compliance, investigator's decision related to an adverse event). Source data are provided as a Source Data file.

Study Type	Dosing Information	Species	Dose (mg/kg)	NOAEL (mg/kg)	MRSD (mg)
4-week repeated toxicity study	Weekly SC for 4 weeks	Cynomolgus monkey	2, 6, 20	6	15
SC, subcutaneous; NOAEL, no-observed-adverse-effect level; MRSD, maximum recommended starting dose; GLP, Good Laboratory Practice.					

Supplementary Table 3. Summary of the GLP 4 weeks repeated-dose toxicity study. Cynomolgus monkeys received weekly subcutaneous injections of PG-102 for 4 weeks at doses of 2, 6, and 20 mg/kg. The no-observed-adverse-effect level (NOAEL) was determined to be 6 mg/kg, supporting a maximum recommended starting dose (MRSD) of 15 mg in humans.

PK parameter	15 mg (n=5)	30 mg (n=6)	30/60 mg (n=6)
**T_{max} (h)**	72.0 [48.0, 72.0]	48.0 [24.0, 72.0]	60.0 [6.0, 72.0]
**$C_{max,ss}$ (ng/mL)**	1020 ± 295	2270 ± 484	2650 ± 1230
**$AUC_{0\text{-}t}$ (μg.h/mL)**	253 ± 55.5	531 ± 110	651 ± 278
**$AUC_{0\text{-}inf}$ (μg.h/mL)**	259 ± 55.5	540 ± 113	660 ± 281
**$t_{1/2}$ (h)**	118 ± 21.4	108 ± 5.96	107 ± 11.0
**V_z/F (L)**	10.3 ± 3.27	9.01 ± 1.88	18.1 ± 13.4
**CL_{ss}/F ($L \cdot h^{-1}$)**	0.06 ± 0.01	0.06 ± 0.02	0.11 ± 0.07

Supplementary Table 4. Summary of pharmacokinetic (PK) parameters of PG-102 following multiple subcutaneous administrations. PK parameters of PG-102 after the final dose at weekly administrations of 15 mg, 30 mg, and 30/60 mg (n = 5–6 per group). Data are presented as mean ± SD (standard deviation), except T_{max}, shown as median [min, max]. Parameters include $AUC_{0\text{–}t}$ (area under the concentration–time curve to the last measurable concentration), $AUC_{0\text{-}inf}$ (area under the concentration–time curve extrapolated to infinity), C_{max} (maximum serum concentration), T_{max} (time to C_{max}), $t_{1/2}$ (elimination half-life), V_z/F (apparent volume of distribution), and CL_{ss}/F (apparent clearance at steady state). Source data are provided as a Source Data file.

Parameter	Placebo (n=6)	PG-102		
		15 mg (n=5)	30 mg (n=6)	30/60 mg (n=6)
Absolute change in body weight (kg)	-1.2 ± 1.5	-1.7 ± 1.8	-2.2 ± 2.1	-3.0 ± 2.0
Percent change in body weight (%)	-1.5 ± 2.1	-2.2 ± 2.3	-2.6 ± 2.5	-3.5 ± 2.2
Body mass index (kg/m^2)	- 0.41 ± 0.53	-0.58 ± 0.62	-0.71 ± 0.67	-0.94 ± 0.59
Change in waist circumference (cm)	-1.8 ± 1.4	-2.6 ± 1.8	-2.3 ± 2.8	-4.1 ± 0.8
Waist–hip ratio	-0.01 ± 0.01	-0.02 ± 0.01	-0.01 ± 0.02	-0.02 ± 0.01
Fat mass (kg, DEXA)	- 0.4 ± 0.8	-0.6 ± 0.3	-0.2 ± 1.4	-0.7 ± 1.3
Lean mass (kg, DEXA)	-1.3 ± 0.6	-1.5 ± 0.9	-2.0 ± 1.3	-2.9 ± 1.5

Supplementary Table 5. Exploratory effect of PG-102 on body weight and body composition parameters in the phase 1 multiple ascending dose (MAD) study. PG-102 was administered once weekly for a total of five doses. Baseline values were obtained prior to the first administration. Post-treatment values were assessed at Day 36 (one week after the last administration), except for dual-energy X-ray absorptiometry (DEXA) scans, which were performed on Day 30 (one day after the final dose). Data are expressed as mean change from baseline (post-dose – pre-dose) and presented as mean ± SD (standard deviation). Data are descriptive, and no formal statistical comparisons were conducted. Source data are provided as a Source Data file.

Parameter	Placebo (n=6)	PG-102		
		15 mg (n=5)	30 mg (n=6)	30/60 mg (n=6)
**HbA1c (%)**				
Pre-dose	5.5 ± 0.3	5.1 ± 0.2	5.5 ± 0.2	5.5 ± 0.3
Post-dose (D28)	5.5 ± 0.4	5.1 ± 0.3	5.3 ± 0.3	5.4 ± 0.3
Post-dose (D57)	5.4 ± 0.4	5.1 ± 0.2	5.2 ± 0.3	5.2 ± 0.4
**FPG (mg/dL)**				
Pre-dose	87.7 ± 6.3	85.8 ± 7.1	85.7 ± 5.3	85.3 ± 8.0
Post-dose (D28)	93.3 ± 6.7	80.2 ± 6.1	80.5 ± 4.3	81.5 ± 4.3
Post-dose (D57)	91.0 ± 8.7	81.0 ± 2.1	86.8 ± 8.2	88.0 ± 4.3
**hsCRP (mg/dL)**				
Pre-dose	0.07 ± 0.03	0.05 ± 0.01	0.07 ± 0.03	0.10 ± 0.03
Post-dose (D28)	0.14 ± 0.07	0.06 ± 0.01	0.14 ± 0.09	0.10 ± 0.05
Post-dose (D57)	0.08 ± 0.04	0.08 ± 0.05	0.08 ± 0.07	0.10 ± 0.05

Supplementary Table 6. Exploratory effect of PG-102 on glycemic and inflammatory biomarker in the phase 1 multiple ascending dose (MAD) study. PG-102 was administered once weekly for a total of five doses. Baseline values were obtained prior to the first administration. Post-dose values were assessed at Day 28 (after four weekly doses, prior to the fifth administration) and Day 57 (four weeks after the last administration). Data are presented as descriptive statistics, and no formal statistical comparisons were conducted. Values are presented as mean ± SD (standard deviation). HbA1c, glycated hemoglobin; FPG, fasting plasma glucose; hsCRP, high-sensitivity C-reactive protein. hsCRP values < 0.03 mg/dL were below the assay detection limit and excluded from mean ± SD calculations; therefore, the number of observations may differ across parameters. Source data are provided as a Source Data file.

Supplementary Note 1. CONSORT 2010 Checklist

Section/Topic	Item No	Checklist item	Reported on page No
**Title and abstract**			
	1a	Identification as a randomised trial in the title	1
	1b	Structured summary of trial design, methods, results, and conclusions (for specific guidance see CONSORT for abstracts)	3
**Introduction**			
Background and objectives	2a	Scientific background and explanation of rationale	5
	2b	Specific objectives or hypotheses	5
**Methods**			
Trial design	3a	Description of trial design (such as parallel, factorial) including allocation ratio	29
	3b	Important changes to methods after trial commencement (such as eligibility criteria), with reasons	N/A
Participants	4a	Eligibility criteria for participants	28
	4b	Settings and locations where the data were collected	28
Interventions	5	The interventions for each group with sufficient details to allow replication, including how and when they were actually administered	29
Outcomes	6a	Completely defined pre-specified primary and secondary outcome measures, including how and when they were assessed	29
	6b	Any changes to trial outcomes after the trial commenced, with reasons	N/A
Sample size	7a	How sample size was determined	29
	7b	When applicable, explanation of any interim analyses and stopping guidelines	N/A
Randomisation:			

Sequence generation	8a	Method used to generate the random allocation sequence	29
	8b	Type of randomisation; details of any restriction (such as blocking and block size)	29
Allocation concealment mechanism	9	Mechanism used to implement the random allocation sequence (such as sequentially numbered containers), describing any steps taken to conceal the sequence until interventions were assigned	29
Implementation	10	Who generated the random allocation sequence, who enrolled participants, and who assigned participants to interventions	29
Blinding	11a	If done, who was blinded after assignment to interventions (for example, participants, care providers, those assessing outcomes) and how	29
	11b	If relevant, description of the similarity of interventions	29
Statistical methods	12a	Statistical methods used to compare groups for primary and secondary outcomes	30-31
	12b	Methods for additional analyses, such as subgroup analyses and adjusted analyses	N/A
**Results**			
Participant flow (a diagram is strongly recommended)	13a	For each group, the numbers of participants who were randomly assigned, received intended treatment, and were analysed for the primary outcome	13, Fig. 6 and Supplementary Table 2
	13b	For each group, losses and exclusions after randomisation, together with reasons	13, Fig. 6 and Supplementary Table 2
Recruitment	14a	Dates defining the periods of recruitment and follow-up	14
	14b	Why the trial ended or was stopped	NA
Baseline data	15	A table showing baseline demographic and clinical characteristics for each group	Table 1
Numbers analysed	16	For each group, number of participants (denominator) included in each analysis and whether the analysis was by original assigned groups	Fig. 6 and Supplementary Table 2

Outcomes and estimation	17a	For each primary and secondary outcome, results for each group, and the estimated effect size and its precision (such as 95% confidence interval)	14, 15, Supplementary Tables 4, 5, 6
	17b	For binary outcomes, presentation of both absolute and relative effect sizes is recommended	NA
Ancillary analyses	18	Results of any other analyses performed, including subgroup analyses and adjusted analyses, distinguishing pre-specified from exploratory	NA
Harms	19	All important harms or unintended effects in each group (for specific guidance see CONSORT for harms)	NA
**Discussion**			
Limitations	20	Trial limitations, addressing sources of potential bias, imprecision, and, if relevant, multiplicity of analyses	22
Generalisability	21	Generalisability (external validity, applicability) of the trial findings	22
Interpretation	22	Interpretation consistent with results, balancing benefits and harms, and considering other relevant evidence	22
**Other information**			
Registration	23	Registration number and name of trial registry	29
Protocol	24	Where the full trial protocol can be accessed, if available	32
Funding	25	Sources of funding and other support (such as supply of drugs), role of funders	38

Citation: Schulz KF, Altman DG, Moher D, for the CONSORT Group. CONSORT 2010 Statement: updated guidelines for reporting parallel group randomised trials. BMC Medicine. 2010;8:18.
© 2010 Schulz et al. This is an Open Access article distributed under the terms of the Creative Commons Attribution License (http://creativecommons.org/licenses/by/2.0), which permits unrestricted use, distribution, and reproduction in any medium, provided the original work is properly cited.

*We strongly recommend reading this statement in conjunction with the CONSORT 2010 Explanation and Elaboration for important clarifications on all the items. If relevant, we also recommend reading CONSORT extensions for cluster randomised trials, non-inferiority and equivalence trials, non-pharmacological treatments, herbal interventions, and pragmatic trials. Additional extensions are forthcoming: for those and for up-to-date references relevant to this checklist, see www.consort-statement.org.

Supplementary Note 2. Clinical Study Protocol (Redacted)

PROTOCOL

A double-blind, randomized, placebo controlled, combined single (Part A) and multiple (Part B, Part C) ascending dose, phase 1 study to investigate the safety, tolerability and pharmacokinetic and pharmacodynamics following subcutaneous injections of PG-102(MG12) in healthy adult and obesity participants

**Investigational medicinal product**	PG-102(MG12)
**Protocol No.**	SL-MG12-P1
**Protocol Version**	8.0
**Effective Date**	2024-10-07
**Study Phase**	Phase 1

CONFIDENTIALITY STATEMENT

All information included in this clinical trial protocol is provided for the purposes of the principal investigator, clinical trial personnel, Institutional Review Board (IRB), Data Safety Monitoring Board (DSMB), and regulatory authorities. Except for obtaining written informed consent from individuals receiving the investigational medicinal product as part of the trial, the information may not be disclosed to any third party without prior written consent from ProGen Co., Ltd.

Table of Contents

DEFINITIONS OF TERMS AND ABBREVIATIONS 38

1. TITLE, PHASE, AND AMENDMENT HISTORY OF THE STUDY 39

1.1. Title 39
1.2. Phase 39
1.3. Clinical Trial Protocol Identification Number and Revision History 39

2. PROTOCOL SUMMARY 40

2.1. Synopsis 40
2.2. Scheme 50
2.2.1. Study Schedule: Single Ascending Dose of PG-102 (MG12) [Part A] 53
2.2.2. Study Schedule: Multiple Ascending Dose of PG-102 (MG12) [Part B], [Part C] 57

3. INTRODUCTION 1

3.1. Study Rationale 1
3.2. Background 1
3.2.1. Summary of Key Non-Clinical Study Results 1
3.2.2. Rationale for Dose Selection in Clinical Design 4
3.3. Benefit/Risk Assessment 5

4. OBJECTIVES 6

4.1. Primary Objective 6
4.2. Secondary Objective 6
4.3. Exploratory Objective 6

5. STUDY DESIGN 7

5.1. Overall Design 7
5.1.1. [Part A] PG-102(MG12) Single Dose Administration 7
5.1.2. [Part B] and [Part C] PG-102(MG12) Multiple Dose Administration 8
5.2. Overview of Study Periods 9
5.3. Scientific Rationale for Study Design 9
5.3.1. Theoretical Rationale for the Endpoints 9
5.3.2. Theoretical Rationale for the Use of Placebo 10
5.4. Rationale for Dose Selection and Dose Escalation Criteria (Part A) 10
5.4.1. Rationale for Starting Dose Selection 10
5.4.2. Dose Escalation and Discontinuation Criteria 10
5.5. Rationale for Dose Selection and Dose Escalation Criteria (Part B and Part C) 11
5.5.1. Rationale for Starting Dose Selection 11
5.5.2. Theoretical Rationale for the Study Design 11
5.5.3. Theoretical Rationale for Dosing Interval 11
5.5.4. Basis for Dose Selection in Part C 13

6. STUDY POPULATION 14

6.1. Number of Subjects 14
6.2. Inclusion Criteria 14
6.3. Exclusion Criteria 14
6.4. Lifestyle Considerations 16
6.4.1. Meal and Dietary Restrictions 16
6.4.2. Activity Restrictions 16
6.4.3. Screen Failures 16
6.4.4. Substitution of Study Subjects 16

7. OVERVIEW AND MANAGEMENT OF CLINICAL INVESTIGATIONAL MEDICINAL PRODUCTS 17

7.1. Study Intervention 17
7.1.1. PG-102(MG12) 17
7.1.2. Placebo 17
7.2. Handling, Storage, and Inventory Management 17
7.3. Preparation and Disbursement 18

8. ADMINISTRATION OF INVESTIGATIONAL MEDICINAL PRODUCT 19

8.1. PG-102(MG12) 19
8.2. Placebo 19
8.3. Randomization 19
8.4. Blinding and Unblinding 19
8.5. Dose Adjustment 20
8.5.1. Dose Escalation and Repeated Administration 20
8.5.2. Criteria for Dose Escalation and Repeated Administration Discontinuation 21
8.6. Concomitant medication and treatment 21
8.6.1. Concomitant Medications and Treatments 21
8.6.2. Concomitant Medications and Treatments 21

9. CLINICAL TRIAL PROCEDURES AND EVALUATION 22

9.1. General Procedures 22
9.1.1. Subject Consent and Screening Number Assignment 22
9.1.2. Collection of demographic data 22
9.1.3. Medical History Investigation 22
9.1.4. Confirm Eligibility 22
9.1.5. Randomization 23
9.1.6. Assignment 23
9.1.7. Administration of the investigational medicinal product(IMP) 23
9.2. Clinical Procedures and Evaluation 24
9.2.1. Physical examinations 24
9.2.2. Body measurements 24
9.2.3. Vital signs 24
9.2.4. Concomitant medications 25
9.2.5. Electrocardiography (12-lead ECG) 25
9.2.6. Laboratory tests and fasting blood glucose 25
9.2.7. Pregnancy tests 25
9.2.8. Blood sampling for PK 26
9.2.9. Blood sampling for immunogenicity (ADA) 26
9.2.10. Oral glucose tolerance test (OGTT) 26
9.2.11. AE assessment 27
9.3. Visit Schedule 27
9.3.1. Screening Visit (SV) 27
9.3.2. [Part A] PG-102(MG12) Single Dose Administration 28
9.3.3. [Part B] and [Part C] PG-102(MG12) Multiple Dose Administration 29
9.3.4. End of Study Visit 33
9.3.5. Unscheduled visit 34
9.4. Lifestyle and Dietary Restrictions. 34
9.5. Safety Evaluation 34
9.6. Pharmacokinetic Evaluation 34
9.7. Pharmacodynamic Evaluation 34
9.8. Exploratory Evaluation 34

10. COMPLETION OF THE TRIAL AND WITHDRAWAL OF SUBJECTS 36

10.1. Criteria for Completion of Clinical Trial Subjects 36
10.2. Criteria for Early Termination of Clinical Trials 36
10.3. Criteria for Participant Early Withdrawal from the Trial 36

11. METHODS FOR ASSESSING SAFETY, EVALUATION CRITERIA, AND REPORTING PROCEDURES, INCLUDING ADVERSE EVENTS 38

11.1.1. Definitions of Safety-Related Terms 38
11.1.2. Evaluation Criteria for Adverse Events 39
11.1.3. Reporting Methods 41
11.1.4. Other Reportable Information 42
11.1.5. Follow-up of Adverse Events 42

12. DATA ANALYSIS AND STATISTICAL CONSIDERATIONS 43

12.1. Analysis Groups 43
12.2. Statistical Analysis Methods 43
12.2.1. Demographic and Clinical History Data 43
12.2.2. Analysis of Safety Evaluation Parameters 43
12.2.3. Analysis of Pharmacodynamic (PD) Evaluation Parameters 44
12.2.4. Analysis of Pharmacokinetic (PK) Evaluation Parameters 44
12.2.5. Interim Analysis 44

13. DATA MANAGEMENT 45

13.1. Record Management and Access 45
13.1.1. Source Documents 45
13.1.2. Data Collection 45
13.1.3. Record Protection and Retention 45
13.1.4. Data Safety Monitoring Plan 45

14. ETHICAL CONSIDERATIONS AND ADMINISTRATIVE PROCEDURES 46

14.1.1. Good Clinical Practice and the Declaration of Helsinki 46
14.1.2. Institutional Review Board (IRB) 46
14.2. Subject Confidentiality 47
14.3. Subject Compensation Agreement 47

15. PROTOCOL AMENDMENT 47

16. PROTOCOL VIOLATIONS AND DEVIATIONS 47

17. QUALITY CONTROL AND QUALITY ASSURANCE 48

18. INVESTIGATOR INFORMATION 48

19. APPENDIX 49

19.1. Appendix 1 Laboratory Test 49
19.2. Appendix 2 Measurement of weight, height, and waist circumference 51
19.3. Appendix 3 Self-Blood Glucose Monitoring 53
19.4. Appendix 4. Injection site reaction 54

20. LIST OF ATTACHMENTS 55

21. REFERENCE 56

List of Tables

[Table 1] Dosing Regimen and Dosage for Cohorts B1–B3 and Cohorts C1, C2 9
[Table 2] Pharmacometric PK Simulation Results for Repeated Dosing 12
[Table 3] Anticipated Drug Exposure Based on Body Mass Index (BMI) for Cohorts B3, C1, and C2 13
[Table 4] Composition of PG-102(MG12) Finished Pharmaceutical Product 6 mg/mL and 50 mg/mL 17
[Table 5] Criteria for Stopping Dose Escalation of Investigational medicinal product(Adverse Event Incidence Rate%) 21
[Table 6] Severity assessment scale for adverse events not included in the NCI CTCAE 39

List of Figures

[Figure 1] Schema [Part A] PG-102(MG12) Single Dose Administration 50
[Figure 2] Schema [Part B] PG-102(MG12) Multiple Dose Administration 51
[Figure 3] Schema [Part B] PG-102(MG12) Multiple Dose Administration 52
[Figure 4] PG-102(MG12) Single ascending dose Phase 7
[Figure 5] PG-102(MG12) Multiple ascending dose 8
[Figure 6] Pharmacometric PK Simulation Results for Repeated Dosing in Part B 12
[Figrue 7] Pharmacometric PK Simulation Results for Repeated Dosing in Part C 13

Definitions of Terms and Abbreviations

ADA	Anti Drug Antibody
AE	Adverse Event, Adverse Experience
ALT	Alanine Amino Transferase, SGPT
AST	Aspartate Amino Transferase, SGOT
BMI	Body Mass Index
CrCL	Creatinine clearance
DEXA	Dual energy X-ray absorptiometry
EOS	End of study
EOT	End of treatment
FIH	First in Human
GCP	Good clinical practice
GLP-1	Glucagon-Like Peptide -1
GLP-2	Glucagon-Like Peptide -2
HbA1c	Glycated hemoglobin (HbA1c)
HBsAg	Hepatitis B Surface Antigen
HCV	Hepatitis C Virus
HED	Human equivalent dose
HIV	Human Immunodeficiency Virus
hsCRP	high sensitivity C-reactive protein
ICH	International council on harmonization
IRB	Institutional review board
KGCP	Korean Good Clinical Practice
MedDRA	Medical Dictionary for Regulatory Activities
MRSD	Maximum Recommended Starting Dose
NOAEL	No Observed Adverse Effect Level
NOEL	No Observed Effect Level,
NTIG	NTIG® (Neo Tri-ImmunoGlobulin)
OGTT	Oral glucose tolerance test
OB	Obesity
PAD	Pharmacologically active dose
PD	Pharmacodynamics
PK	Pharmacokinetics
SAE	Serious Adverse Event
SD rat	Sprague-Dawley rat
SGLT-2	Sodium-glucose cotransporter-2
SRC	Safety Review Committee
TEAE	Treatment Emergent Adverse Event

1. Title, Phase, and Amendment History of the Study

1.1. Title

A double-blind, randomized, placebo controlled, combined single (Part A) and multiple (Part B, Part C) ascending dose, phase 1 study to investigate the safety, tolerability and pharmacokinetic and pharmacodynamics following subcutaneous injections of PG-102(MG12) in healthy adult and obesity participants

1.2. Phase

Phase 1

1.3. Clinical Trial Protocol Identification Number and Revision History

Version	Date	Comment
1.0	2023. 05. 19	Established
2.0	2023. 07. 24	Revision of clinical trial design
2.1	2023. 07. 27	Modification of the phrase
3.0	2023. 08. 14	Addition of investigational product MG12 50 mg/mL, Typo correction
3.1	2023. 10. 17	Clarification of content and correction of typos
3.2	2023. 11. 27	Revision of method of drug administration
4.0	2024. 01. 18	[Part B] Change of Design<br>Change in drug name from MG12 to PG-102 (MG12)
4.1	2024. 01. 25.	Clarification of fasting blood glucose testing
4.2	2024. 02. 02	Clarification of fasting blood glucose testing, replacement of pre-dose duplicate glucose test
5.0	2024. 03. 11	[Part B] Change of Design
5.1	2024. 04. 01	Addition of interim analysis items
6.0	2024. 05. 22	[Part B] Clarification of Cohort Changes and Interim Analysis Items
7.0	2024. 06. 24	[redacted]
7.1	2024. 07. 10	[redacted]
7.2	2024. 08. 16	Clarification of specimen collection for oral glucose tolerance test and gut microbiome test
8.0	2024. 10. 07	[redacted]

2. Protocol Summary

2.1. Synopsis

**Protocol Number**	SL-MG12-P1
**Protocol Title**	A double-blind, randomized, placebo controlled, combined single (Part A) and multiple (Part B, Part C) ascending dose, phase 1 study to investigate the safety, tolerability and pharmacokinetic and pharmacodynamics following subcutaneous injections of PG-102(MG12) in healthy adult and obesity participants
**Study Phase**	Phase 1
**Study Design**	A randomized, placebo-controlled, double-blind, single (Part A) and multiple-dose (Part B and Part C) escalation dose phase I study
**Sponsor**	Progen<br>Address: 172, Magokjungang-ro, Gangseo-gu, Seoul, Republic of Korea
**Trial Site**	Appendix 1. Information on clinical trial sponsor, clinical trial institution, and investigator
**Trial Period**	A 24-month period from the date of approval (may be subject to change depending on the enrollment rate of subjects)
**Subject**	Healthy adult subject, Obese subject
**Object**	[redacted]<br>[Part B] Multiple dose administration of PG-102(MG12) in healthy adult subject<br>1) Primary objective<br>• To evaluate the safety and tolerability of repeated subcutaneous administration of PG-102 (MG12) in healthy adult subjects by assessing treatment-emergent adverse events (TEAEs).<br>2) Secondary objective<br>• To evaluate the pharmacokinetics of PG-102 (MG12) following repeated subcutaneous administration in healthy adult subjects.<br>• To evaluate the pharmacodynamics of PG-102 (MG12) following repeated subcutaneous administration in healthy adult subjects.<br>3) Exploratory objective<br>• To assess inflammatory-related biomarkers, specifically plasma hsCRP.<br>[redacted]

	[redacted]
**Investigational Product**	PG-102(MG12)(Investigational medicinal product)<br>• Composition: GLP-1/GLP-2 hyFc<br>• Appearance and formulation: A vial containing a colorless, clear PG-102(MG12) injectable solution<br>• Contents<br>PG-102(MG12) 6 mg/mL (1 mL/vial)<br>PG-102(MG12) 50 mg/mL (0.6 mL/vial)<br>• Storage conditions: Store under refrigeration (2-8℃)<br>0.9% Sodium Chloride Injection (Placebo)<br>• Composition: Sodium chloride<br>• Storage conditions: Store in a tightly closed container a controlled room temperature (1-30℃)
**Dosing regimen of the investigational drug**	[redacted]<br>In Part B [redacted], subcutaneous injections will be administered once weekly (q1w) for a total of 5 repeated doses.
**Study procedures**	[redacted]

<table>
<tr><td></td><td>[redacted]

[Part B] Repeated Administration of PG-102 (MG12): Each cohort will enroll 8 subjects, with 6 receiving the investigational drug (PG-102 [MG12]) and 2 receiving placebo (0.9% normal saline for injection). The investigational drug and placebo will be administered subcutaneously at 1-week intervals for a total of 5 doses.

Cohort S (optional) may enroll 8 subjects, with the optimal dose of the investigational drug (PG-102 [MG12]) and placebo (0.9% normal saline for injection) administered subcutaneously at 2-week intervals for a total of 5 doses.

[redacted]

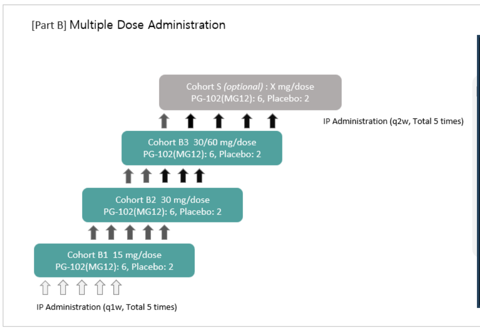

[redacted]</td></tr>
<tr><td>Dosing escalation criteria and discontinuation criteria</td><td>1) Dose escalation and repeated dose criteria:
① Dose escalation will proceed from low to high doses.
② The dose for the next stage will be determined after reviewing the following data:
[redacted]</td></tr>
</table>

	[redacted]<br>[Part B] Repeated Administration of PG-102 (MG12): Cohort B1 will proceed based on the safety data from Cohort A3, Cohort B2 will proceed based on the safety data from Cohort A4 and Cohort B1, Cohort B3 will proceed based on the safety data from Cohort A4 and Cohort B2.<br>[redacted]<br>The investigator will evaluate the data according to the dose escalation and repeated dose discontinuation criteria to determine whether to proceed with dose escalation and/or repeated dosing. Safety information and decisions regarding dose escalation/repeated dosing will be reviewed by a Safety Review Committee (SRC) to ensure there are no safety concerns before initiating the next dose of the investigational drug and/or repeated dosing.<br>2) Dose escalation and repeated dose discontinuation criteria:<br>Dose escalation and/or repeated dosing of the investigational drug will be temporarily discontinued if any of the following events occur after drug administration. Resumption or permanent discontinuation of dosing will be determined by the Safety Review Committee (SRC).<br><table><tr><td>Severity of adverse events related to the investigational drug</td><td>Incidence rate (%)</td></tr><tr><td>Moderate adverse event*</td><td>50%</td></tr><tr><td>Severe adverse event</td><td>25%</td></tr><tr><td>Serious adverse event</td><td>One or more subjects</td></tr></table>* Only drug-related moderate or severe adverse events that do not resolve with outpatient treatment (i.e., treatment not requiring hospitalization) will be considered in the following situations.
**Number of Subjects**	[redacted]<br>[Part B] Repeated Administration of PG-102 (MG12)<br>Up to 32 subjects: 24 subjects (based on 3 cohorts) and 8 subjects (Cohort S, optional).<br>Each dose group will consist of 8 subjects (6 receiving PG-102 [MG12] and 2 receiving placebo), with up to 4 dose groups (including Cohort S, optional) involving a maximum of 32 subjects.

<table>
<tr><td></td><td>[redacted]</td></tr>
<tr><td>Inclusion Criteria</td><td>The subjects must satisfy all of the following eligibility criteria
1) Male or female participants, aged 18 to 65 years inclusive at the time of signing informed consent
2) Volunteers who have received a detailed explanation of the clinical trial, fully understand it, voluntarily decide to participate, and provide written informed consent
3) Subjects who agree to abstain from alcohol consumption from 48 hours prior to screening and administration of the investigational product, and throughout the hospitalization period until discharge
4) Subjects without congenital or chronic diseases within the past 5 years and with no abnormal findings based on a medical examination
5) Subjects with a body weight of ≥ 55 kg for males and ≥ 50 kg for females at screening, and who meet the following body mass index (BMI) criteria
[redacted]
[Part B]: Subjects with a body mass index (BMI) of ≥ 25 kg/m² and < 30 kg/m² at the time of screening
[redacted]
6) Subjects deemed eligible based on health assessments performed during screening, including medical history, physical examination, vital signs, 12-lead electrocardiogram (ECG), and laboratory tests</td></tr>
<tr><td>Exclusion Criteria</td><td>Subjects who meet any of the following criteria are not eligible to participate in this clinical trial
1) Subjects who have taken prescription drugs, herbal medicines, over-the-counter drugs, or vitamin supplements within 10 days prior to the first administration of the investigational product, or who have used the following drugs and/or other substances within 90 days prior to screening
• Drugs that affect body weight (such as obesity medications, psychiatric drugs, beta blockers, diuretics, contraceptives, female hormones, proton-pump inhibitors (PPI), H2 receptor antagonists, health functional foods/supplements, and formulas designed for weight control)
• Drugs that have the potential to impact blood sugar, liver fat, and intestinal microorganisms (including GLP-1 receptor agonists, DPP-4 inhibitors, SGLT-2 inhibitors, thiazolidinedione (TZDs), fish oil, polyunsaturated fatty acids (PUFA), and ursodeoxycholic acid (UDCA)), as well as</td></tr>
</table>

	individuals who are currently using insulin [redacted]<br>2) Subjects who have participated in other clinical trials or bioequivalence studies and received investigational products within 180 days prior to the first administration of the investigational product<br>3) Subjects who have donated whole blood within 60 days prior to the first administration of the investigational product, donated blood components within 30 days prior, or received a blood transfusion within 30 days prior<br>4) Subjects who do not agree to use dual contraception methods** or to practice abstinence from the time of signing the informed consent form until 90 days after the last administration of the investigational product **Dual contraception methods: intrauterine devices (IUDs), chemical barriers (e.g., spermicides), physical barriers (e.g., male or female condoms), tubal ligation or laparoscopic sterilization, vasectomy, or strict abstinence<br>5) Subjects with a history of clinically significant diseases affecting the cardiovascular, respiratory, renal, endocrine, hematologic, gastrointestinal, central nervous, urogenital, musculoskeletal, or psychiatric systems, or malignancies, or those with active diseases in these systems (except in cases where the condition has been completely cured and does not affect the current health status)<br>6) Subjects with a history of gastrointestinal diseases that may affect the absorption of the investigational product (e.g., Crohn's disease, ulcers, acute or chronic pancreatitis) or a history of gastrointestinal surgery (excluding simple appendectomy or hernia repair)<br>7) Subjects with a history of acute proliferative retinopathy or maculopathy, severe gastroparesis, and/or severe neruopathy<br>8) Subjects with a history of surgical treatment for obesity within 2 years (example: bariatric surgery, gastric banding etc) or gastrointestinal procedures for weight loss (including LAP-BAND®), or uncontrolled gastrointestinal disorders at Screening (e.g., peptic ulcer, gastroesophageal reflux disease)<br>9) Subjects with a history of drug abuse<br>10) Subjects who are unable to abstain from consuming caffeine-containing foods and beverages (e.g., coffee, tea [black tea, green tea, etc.], caffeinated soft drinks, coffee-flavored milk, energy drinks) or from smoking during the period from 9:00 AM on the day of hospitalization until discharge<br>11) Subjects who excessively consume caffeine (> 5 cups/day) or alcohol (> 210 g/week) or are heavy smokers (> 10 cigerettes/day)<br>12) Subjects with a known allergy to GLP-1 or GLP-2 receptor agonists<br>13) Subjects with a history of hypersensitivity or clinically significant allergic

	reactions to investigational products, other drugs, or excipients<br>14) Subjects who have received intravenous administration of radiopaque iodine contrast agents (e.g., Intravenous urography, angiography, intravenous cholangiography, or contrast-enhanced computed tomography) within 48 hours prior to the first administration of the investigational product<br>15) Subjects who, after resting for at least 5 minutes in a sitting position at screening, have systolic blood pressure ≥ 150 mmHg or < 90 mmHg, or diastolic blood pressure ≥ 100 mmHg or < 50 mmHg<br>16) Subjects who have laboratory test results meeting any of the following criteria:<br>• Positive results for serum tests (HBsAg, HCV Ab, HIV Ag/Ab, Syphilis reagin test)<br>• eGFR (estimated Glomerular Filtration Rate) < 60 mL/min/1.73 m^2 based on the CKD-EPI (Chronic Kidney Disease Epidemiology Collaboration) formula<br>• Serum creatinine levels ≥ 1.5 mg/dL in males, ≥ 1.4 mg/dL in females, or creatinine clearance (CrCL) < 50 mL/min in all subjects<br>17) Subjects with significantly abnormal liver function tests or those meeting any of the following conditions<br>• Serum AST or ALT > 1.25 times the upper limit of normal [redacted]<br>• Serum total bilirubin > 1.5 times the upper limit of normal<br>18) Subjects whom the investigator deems unsuitable for participation in the clinical trial for any other reason
**Safety endpoint**	Adverse event (AE), Vital signs, Laboratory tests, Electrocardiogram (ECG), Physical examination, Immunogenicity assessment
**Efficacy endpoint**	[redacted]<br><br>[Part B] [redacted] Repeated Administration of PG-102 (MG12):<br>1) Pharmacokinetic (PK) Evaluation<br>• Evaluation timepoints: Pharmacokinetic evaluations will be conducted before the administration of the investigational drug and at specified time

	points up to Day 56 or Day 84 after administration.<br>• Evaluation parameters: C_{max}, T_{max}, $t_{1/2}$, AUC_{0-t}, AUC_{0-inf}, CL/F, $C_{max,ss}$, $T_{max,ss}$, AUC_{tau}, AUC_{inf}, $C_{avg,ss}$, $C_{trough,ss,}$ etc.<br>2) Pharmacodynamic (PD) Evaluation<br>• Evaluation timepoints: From before the administration of the investigational product to specified time points outlined in the schedule up to Day 56 or Day 84 after initiation of administration.<br>• Evaluation parameters: Changes in body weight, absolute weight loss, percentage weight change, body mass index (BMI), waist circumference, waist-hip ratio, body fat measurement (DEXA), oral glucose tolerance test (OGTT), glycated hemoglobin (HbA1c) levels. absolute weight loss, percentage weight change, body mass index (BMI), waist circumference, waist-hip ratio, body fat measurement (DEXA), oral glucose tolerance test (OGTT), glycated hemoglobin (HbA1c) levels<br>3) Exploratory Biomarker Evaluation<br>• Evaluation timepoints: Assessments will be conducted prior to the administration of the investigational drug (V1/D1), the day before the final administration (V9/D28*), and at the end-of-study visit. For Cohort S (optional), the final pre-administration assessment corresponds to V9/D56.<br>• Evaluation parameters: Evaluation of inflammatory-related biomarkers (hsCRP)<br>[redacted]
**Statistical analysis plan**	1) Definition of analysis sets<br>① Safety analysis set: All subjects who were randomized and received at least one dose of the investigational drug.<br>② Pharmacokinetic analysis set: Subject who received the investigational drug and had quantifiable concentration of PG-102(MG12) in at least one scheduled pharmacokinetics blood sample.<br>③ Pharmacodynamic analysis set: Subject who received the investigational drug and had measurable pharmacodynamic endpoints.<br>④ Immunogenicity analysis set: All subjects who were randomized and received the investigational drug and had any evaluable immunogenicity results for the investigational drug. Subjects who did not receive at least one dose of the investigational drug will be excluded from this analysis set.<br>2) Statistical analysis<br>① Quantitative data will be summarized using mean (arithmetic or geometric), standard deviation, median, minimum, maximum, and for categorical variables, frequency and percentage. More detailed data may be presented if necessary.<br>② Pharmacokinetic assessment may be conducted during the clinical trial as needed, and both the investigator and subject will remain blinded.<br>③ Demographic and other baseline data, safety, and pharmacokinetic/pharmacodynamic data will be summarized using descriptive statistics, including absolute values or changes from baseline at each measurement time point, and presented by treatment group and dose

	group.<br>If necessary, shift tables (or plots) will be presented by treatment group and dose group for variables that are expected to have clinically meaningful differences between dose groups or before and after dosing. Appropriate statistical tests will be conducted.<br>④ Pharmacokinetic parameters for PG-102(MG12) will be calculated using non-compartmental methods, and descriptive statistics will be presented by treatment group and dose group to assess linearity and dose-proportionality.<br>⑤ Concomitant medications will be recorded using the Anatomical Therapeutic Chemical (ATC) classification system. Adverse events will be standardized using the Medical Dictionary for Regulatory Activities (MedDRA) by System Organ Class (SOC) and Preferred Term (PT).<br>⑥ Interim analyses may be conducted to assess safety, pharmacokinetics, and pharmacodynamics for each part or cohort. Interim analyses will be conducted after data lock for the part or cohort to be analyzed.<br>Interim analyses will be conducted only by designated personnel, and the blinding will be broken only for the designated statistical analyst conducting the interim analysis. All procedures will be conducted and documented according to pre-specified procedures.

[redacted]

[Figure 1] Schema [Part A] PG-102(MG12) Single Dose Administration

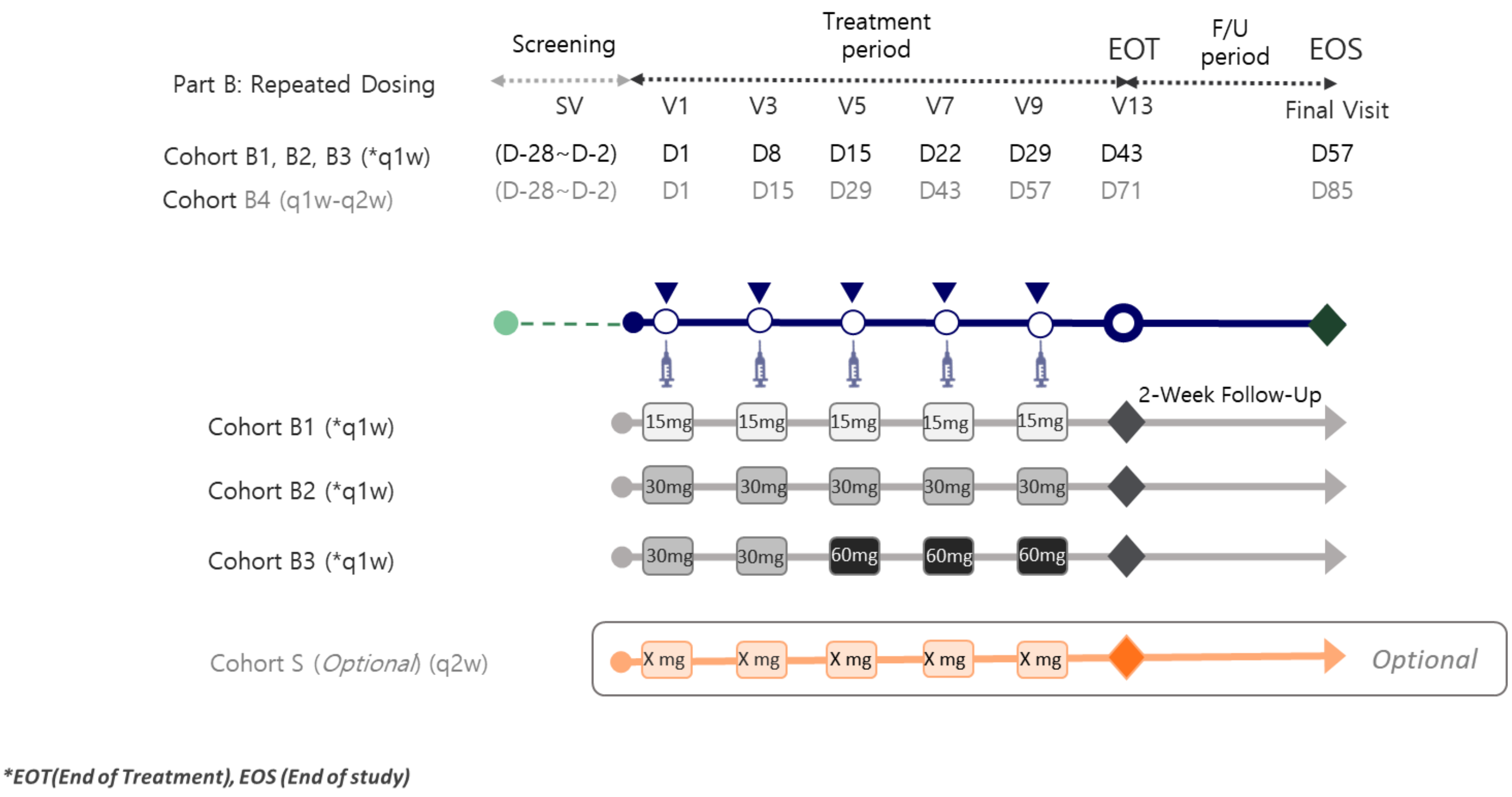

*EOT (end of treatment), EOS (end of study)

[Figure 2] Schema [Part B] PG-102(MG12) Multiple Dose Administration

[redacted]

[Figure 3] Schema [Part C] PG-102(MG12) Multiple Dose Administration

2.2.1. Study Schedule: Single Ascending Dose of PG-102 (MG12) [Part A]

[redacted]

	Screening	Treatment period							End of Study
**Visit**	**SV**	**V1/D-1**	**V1/D1**	**V1/D2**	**V1/D3**	**V2**	**V3**	**V4**	**EOS**
**Visit Date (Days)**	**D-28 ~ -2**	**D-1**	**D 1**	**D 2**	**D 3**	**D 5**	**D 8**	**D 15**	**D 29**
**Visit window(±) Ds**		**0**				**0**	**0**	**0**	**±1**
Electrocardiography (12-lead ECG) 8)	X	X	X	X	X	X	X	X	X
Hematology test 9)	X	X	X	X		X		X	X
Blood chemistry test 9)	X	X	X	X		X		X	X
Blood coagulation test 9)	X	X	X	X		X		X	X
Urinalysis 9)	X	X		X		X		X	X
Serology 9)	X								
Pregnancy test 10)	X	X							X
**Administration of IMP**									
Administration of IMP 11)			X						
**Clinical assessments**									
Blood sampling for PK 12)			X	X	X	X	X	X	X
Weight 13)	X	X					X	X	X
Body measurements (height,waist-hip) 13)		X					X	X	X
HbA1c 14)	X	(X)							X

[redacted]

[redacted]

2.2.2. Study Schedule: Multiple Ascending Dose of PG-102 (MG12) [Part B][redacted]

	Screening	Treatment period																				End of Study
**Visit**	**SV**	**V1**		**V2**	**V3**		**V4**	**V5**		**V6**	**V7**		**V8**	**V9**				**V10**	**V11**	**V12**	**V13**	**EOS**
**Visit Date: Cohort B1~B3, C1, C2**	**D-28 ~ -2**	**D-1**	**D 1**	**D3**	**D 7**	**D8**	**D10**	**D14**	**D15**	**D17**	**D21**	**D22**	**D24**	**D28**	**D29**	**D30**	**D31**	**D32**	**D34**	**D36**	**D43**	**D57**
**Visit Date(Cohort S: optional group)** [1]					***D14***	***D15***	***D17***	***D28***	***D29***	***D31***	***D42***	***D43***	***D45***	***D56***	***D57***	***D58***	***D59***	***D60***	***D62***	***D64***	***D71***	***D85***
**Visit window(±) Ds**	**D-28 ~ -1**	**0**		**±1**	**0**		**±1**	**0**		**±1**	**0**		**±1**	**0**				**0**	**0**	**0**	**0**	**±1**
**General**																						
Informed consent [2]	X																					
Demographic and Medical history [3]	X	X	X																			
Inclusion/Exclusion [4]	X	X																				
Assignment [5]		X																				
**Clinical procedures**																						
Hospitalization [6]		X			X			X			X			X								
Discharge [6]			X			X			X			X					X					
Outpatient visit [6]																		X	X	X	X	X
Telephone visit [6]				X			X			X			X									
Concomitant medications	X	X	X	X	X	X	X	X	X	X	X	X	X	X	X	X	X	X	X	X	X	X
Vital signs [7]	X	X	X		X	X		X	X		X	X		X	X	X	X	X	X	X	X	X
Physical examination [8]	X	X	X		X	X		X	X		X	X		X	X	X	X	X	X	X	X	X
Electrocardiography (12-lead ECG) [9]	X							X									X					X

	Screening	Treatment period																				End of Study
**Visit**	**SV**	**V1**		**V2**	**V3**		**V4**	**V5**		**V6**	**V7**		**V8**	**V9**				**V10**	**V11**	**V12**	**V13**	**EOS**
**Visit Date: Cohort B1~B3, C1, C2**	**D-28 ~ -2**	**D-1**	**D 1**	**D3**	**D 7**	**D8**	**D10**	**D14**	**D15**	**D17**	**D21**	**D22**	**D24**	**D28**	**D29**	**D30**	**D31**	**D32**	**D34**	**D36**	**D43**	**D57**
**Visit Date(Cohort S: optional group)** [1]					***D14***	***D15***	***D17***	***D28***	***D29***	***D31***	***D42***	***D43***	***D45***	***D56***	***D57***	***D58***	***D59***	***D60***	***D62***	***D64***	***D71***	***D85***
**Visit window(±) Ds**	**D-28 ~ -1**	**0**		**±1**	**0**		**±1**	**0**		**±1**	**0**		**±1**	**0**				**0**	**0**	**0**	**0**	**±1**
Hematology test [10]	X	X						X						X			X			X	X	X
Blood chemistry test [10]	X	X						X						X			X			X	X	X
Blood coagulation test [10]	X	X												X			X					X
Urinalysis [10]	X	X						X						X			X			X	X	X
Serology [10]	X																					
Fasting glucose[11]			X			X			X			X			X							
Pregnancy test [12]	X	X																				X
**Administration of IMP**																						
Administration of IMP [13]			X			X			X			X			X							
**Clinical assessments**																						
Blood sampling for PK [14]			X			X			X			X			X	X	X	X	X	X	X	X
Weight [15]	X	X			X			X			X			X						X	X	X
Body measurements (height,waist-hip) [15]		X			X			X			X			X						X	X	X
Body fat measurements [16]		X														X						
HbA1c [17]	X							X*						X								X

	Screening	Treatment period																				End of Study
**Visit**	**SV**	**V1**		**V2**	**V3**		**V4**	**V5**		**V6**	**V7**		**V8**	**V9**				**V10**	**V11**	**V12**	**V13**	**EOS**
**Visit Date: Cohort B1~B3, C1, C2**	**D-28 ~ -2**	**D-1**	**D 1**	**D3**	**D 7**	**D8**	**D10**	**D14**	**D15**	**D17**	**D21**	**D22**	**D24**	**D28**	**D29**	**D30**	**D31**	**D32**	**D34**	**D36**	**D43**	**D57**
**Visit Date(Cohort S: optional group)** [1]					***D14***	***D15***	***D17***	***D28***	***D29***	***D31***	***D42***	***D43***	***D45***	***D56***	***D57***	***D58***	***D59***	***D60***	***D62***	***D64***	***D71***	***D85***
**Visit window(±) Ds**	**D-28 ~ -1**	**0**		**±1**	**0**		**±1**	**0**		**±1**	**0**		**±1**	**0**				**0**	**0**	**0**	**0**	**±1**
Oral glucose tolerance test (OGTT) [18]		X														X						
**Exploratory Evaluations**																						
Inflammatory biomarker [19]			X											X								X
[redacted]		X														X						
**Safety Evaluations**																						
AE assessment [21]			X	X	X	X	X	X	X	X	X	X	X	X	X	X	X	X	X	X	X	X
Self-Blood Glucose Monitoring [22]			X																			
Immunogenicity (ADA) [23]		X													X							X

1) Every 2 weeks, subjects in Cohort S (optional group) will be visited according to the visit schedule starting from V3.
2) Subjects must be informed and written informed consent obtained prior to starting any procedures including screening.
3) Demographic information includes date of birth, age, and sex. Medical history includes any clinically significant medical conditions or abnormal findings prior to administration of the investigational drug. Congenital and chronic diseases within the past 5 years, and hypersensitivity to the investigational drug components, regardless of the duration, will be investigated.
4) On D-1 visit, the subject number assignment will be reconfirmed.
5) Subject numbers will be assigned in the order of passing the screening within 24 hours from the scheduled first hospitalization.
6) For the first four doses, subjects will be admitted the day before drug administration and discharged 6 hours after dosing. After the fifth and final dose, subjects will be

admitted for V9/D28* for pharmacokinetic evaluation and discharged 48 hours post-dose. Between doses, safety follow-ups will be conducted via telephone (V2, V4, V6, V8); all other visits will be outpatient. *For Cohort S (optional group), the final admission will be at V9/D56.

7) Vital signs include pulse, temperature, blood pressure, and respiratory rate. Blood pressure, pulse, and respiratory rate are measured after resting in a sitting position for more than 5 minutes. During hospitalization, vital signs will be conducted at the following times:
   - For the first administration of the investigational drug (V1): Pre-dose (within 1 hour before dosing), 30 minutes ± 15 minutes post-dose, 4 hours ± 1 hour post-dose, 6 hours ± 1 hour post-dose (V1/D1)
   - For the 2nd to 4th administrations of the investigational drug (V3, V5, V7): Pre-dose (within 1 hour before dosing), 4 hours ± 1 hour post-dose, 6 hours ± 1 hour post-dose (V3/D8*, V5/D15*, V7/D22*)
   - For the last administration of the investigational drug (V9): Pre-dose (within 1 hour before dosing), 4 hours ± 1 hour post-dose, 8 hours ± 1 hour post-dose, 12 hours ± 1 hour post-dose (V9/D29*), and 24 hours ± 1 hour, 36 hours ± 1 hour post-dose (V9/D30*), and pre-discharge (V9/D31*)

   *For Cohort S (optional group), please refer to the corresponding date in the table.

8) Physical examinations will be conducted at each visit, and any clinically significant findings will be reported as adverse events. During hospitalization, physical examinations will be conducted at the following times:
   - For the 1st to 4th administrations of the investigational drug (V1, V3, V5, V7): Pre-dose (within 1 hour before dosing), 4 hours ± 1 hour post-dose, 6 hours ± 1 hour post-dose (V1/D1, V3/D8*, V5/D15*, V7/D22*)
   - For the last administration of the investigational drug (V9): Pre-dose (within 1 hour before dosing), 4 hours ± 1 hour post-dose, 8 hours ± 1 hour post-dose, 12 hours ± 1 hour post-dose (V9/D29*), and 24 hours ± 1 hour, 36 hours ± 1 hour post-dose (V9/D30*), and pre-discharge (V9/D31*) *For Cohort S (optional group), please refer to the corresponding date in the table.

   *For Cohort S (optional group), please refer to the corresponding date in the table.

9) Subjects should relax and maintain a stable position for at least 10 minutes before the measurement. Electrocardiograms (ECG) may be re-tested at the discretion of the investigator (or delegated sub-investigator) if the heart rate is irregular or if there are any significant changes. Furthermore, the investigator (or delegated sub-investigator) may perform the test at any time if it is deemed clinically necessary.
10) If the subject is admitted within 7 days after the screening visit (V1/D-1), the results of the laboratory tests (hematology, clinical chemistry, coagulation, urinalysis, and serum tests) can be used as the results for V1/D-1. Furthermore, the investigator (or delegated sub-investigator) may perform the test at any time if it is deemed clinically necessary.
11) A fasting blood glucose test will be performed using a glucose meter within 1 hour prior to the administration of the investigational drug to confirm that the fasting blood glucose level is 70 mg/dL or above. If the fasting blood glucose level is less than 70 mg/dL, the subject will be instructed to consume 15 g of sugar and a repeat test will be performed 15 minutes later. The investigational drug will be administered only if the fasting blood glucose level is 70 mg/dL or above after the repeat test.
12) A pregnancy test will be conducted only for women of childbearing potential who have the possibility of becoming pregnant. This test will not be conducted in cases of surgical infertility (hysterectomy or bilateral tubal ligation) or postmenopausal women (at least 12 months have passed since non-therapy induced amenorrhea).

13) The investigational drug will be administered once a week (V1/D1, V3/D8, V5/D15, V7/D22, V9/D29). Subjects will be hospitalized the day before dosing and the drug will be administered on the first day of hospitalization. For Cohort S (optional group), the drug will be administered every two weeks (V1/D1, V3/D15, V5/D29, V7/D43, V9/D57).
14) Blood sampling for pharmacokinetic (PK) parameter assessment will be performed at the following time points:
    - For the first 4 doses of the investigational drug (V1, V3, V5, V7): Pre-dose (within 0.5 hours before dosing), 4 hours ± 15 minutes post-dose, 6 hours ± 15 minutes post-dose (V1/D1, V3/D8*, V5/D15*, V7/D22*).
    - For the last dose of the investigational drug (V9 to end-of-study visit): Pre-dose (within 0.5 hours before dosing), 4 hours ± 15 minutes post-dose, 6 hours ± 15 minutes post-dose, 12 hours ± 30 minutes post-dose (V9/D29*), 24 hours ± 1 hour post-dose, 36 hours ± 1 hour post-dose (V9/D30*), 48 hours ± 2 hours post-dose (V9/D31*), 72 hours ± 2 hours post-dose (V10/D32*), 120 hours ± 2 hours post-dose (V11/D34*), 168 hours ± 2 hours post-dose (V12/D36*), 336 hours ± 2 hours post-dose (V13/D43*), end-of-study visit (D57*).

    *For Cohort S (optional group), please refer to the corresponding date

15) Height, weight, waist and hip circumference will be measured according to Appendix Body measurements. Height will be measured only at the screening visit.
16) Dual-energy X-ray absorptiometry (DEXA) will be used to measure body composition. This measurement will be performed on days V1/D-1 to D1, prior to drug administration, and again on days V9/D30* to D31*. *For subjects in Cohort S (optional group), the DEXA scan will be conducted on days V9/D58 and D59.
17) HbA1c will be measured at screening and V9/D28, and at the end-of-study visit for cohorts B1-B3, C1, and C2. For Cohort S (optional group), HbA1c will be measured at screening, V5/D28*, V9/D56, and at the end-of-study visit.

18) According to the Clinical Practice Guideline for Diabetes 2024 in Korean Diabetes Association, the Oral Glucose Tolerance Test (OGTT) will be conducted. Fasting blood glucose will be measured after at least 8 hours of fasting. Subsequently, the subject will ingest 75g of glucose dissolved in 250-300mL of water or a commercial glucose solution within 5 minutes.

    In Part B, blood glucose levels will be measured and assessed at 30, 60, 90, and 120 minutes after glucose ingestion.

    [redacted] *For Cohort S (optional group), this corresponds to V9/D58.*

18) Plasma hs-CRP will be measured as an inflammatory biomarker at V1/D1 (predose), V9/D28*, and the end-of-study visit. For Cohort S (optional group), this corresponds to V9/D56.
19) Stool samples for gut microbiome analysis will be collected on the day prior to the first investigational drug administration (V1/D1) during the first hospitalization for cohorts C1 and C2. Additional stool samples will be collected prior to discharge (V9/D31) during the fifth hospitalization (V9/D30).
20) At each visit, all adverse events (AEs) and serious adverse events (SAEs) that have occurred since the previous visit or that are ongoing will be identified and recorded.

Medical conditions or abnormal findings of clinical significance prior to the administration of the investigational drug will be collected as medical history. Additional AE assessments will be conducted 4 ± 1 hours and 6 ± 1 hours after drug administration on the day of dosing. Local reactions at the injection site will be assessed immediately after dosing, 30 ± 10 minutes post-dose, and 1 hour ± 10 minutes post-dose and recorded.

21) During the V1 hospitalization period, subjects will be educated on hypoglycemic symptoms and management procedures. They will also be provided with a self-monitoring blood glucose (SMBG) device and trained on its usage. Subjects will perform daily SMBG from the investigational drug administration day (V1/D1) until 7 days post the last administration (V12). However, blood glucose measurements during outpatient visits or hospitalization can be conducted by a delegated study staff member and may be replaced by a fasting blood glucose test performed prior to dosing. The SMBG device distributed at V1 will be collected at V12 or a subsequent visit.
22) Blood will be collected for immunogenicity evaluation samples, and conducted at V1/D-1(prior to administration), final administration day(V9/29*) and EOS visit. *For Cohort S (optional group), this corresponds to V9/D57

3. Introduction

3.1. Study Rationale

This clinical study (SL-MG12-P1) is designed to evaluate the safety, tolerability, pharmacokinetics (PK), and pharmacodynamics (PD) of PG-102(MG12) in healthy adult subjects following [redacted] and multiple-dose administration [Part B]. [redacted]

PG-102(MG12) is a bispecific Fc fusion protein developed by fusing a Double GLP-1E-NTIG to one side of the Fc region and a Stable GLP-2-NTIG to the other. It has been designed with biological and immunological properties that regulate blood glucose levels, improve metabolic function, reduce body weight, and enhance gut barrier function (reducing leaky gut) while improving the gut microbiome.

Unlike conventional GLP-1-based drugs, PG-102(MG12) incorporates GLP-2, offering a bispecific protein therapy for metabolic diseases (e.g., obesity, diabetes, and non-alcoholic steatohepatitis [NASH]). Efficacy studies in mouse models of these conditions have demonstrated the dual effects of GLP-1 and GLP-2 (refer to the MG12 Investigator's Brochure).

PG-102(MG12) has shown improved therapeutic effects for metabolic diseases, such as obesity and type 2 diabetes, compared to existing GLP-1-based drugs. Moreover, it is being developed to explore its therapeutic potential for NASH, a condition where GLP-1 drugs are actively under clinical investigation.

Accordingly, this first-in-human (FIH) clinical trial aims to primarily evaluate the safety and tolerability of PG-102(MG12) in healthy adults, with secondary objectives to assess its pharmacokinetics and pharmacodynamics.

3.2. Background

3.2.1. Summary of Key Non-Clinical Study Results

The non-clinical studies for PG-102(MG12) included efficacy, safety pharmacology, and toxicity assessments. For efficacy studies, mouse models of obesity, diabetes, and steatohepatitis were utilized. Prior to conducting efficacy studies, it was confirmed that PG-102(MG12) demonstrated equivalent affinity for both mouse GLP-1 receptors and human GLP-1 receptors. This established the translatability of mouse efficacy results to humans.

In efficacy studies across various disease models, dose-dependent therapeutic effects were observed, including weight reduction, blood glucose regulation, and decreased steatohepatitis markers. The dosing regimen and key results for each disease model are summarized below.

In the obesity model, the minimum effective dose showing efficacy was 1 mg/kg (human equivalent dose [HED]: 0.081 mg/kg), and a dose-dependent reduction in body weight was confirmed.

Title	Test system	Administered substance	Dosage (mg/kg)	Gender/Number of Animals	Key Results
Obesity disease model efficacy test [1)]	C57BL/6 mouse (DIO)	Vehicle MG12	0, 1, 2, 4	Male/9~10, 5 (vehicle)	Dose-dependent weight loss confirmed starting from 1 mg/kg
Diabetic disease model efficacy test [2)]	BKS.Cg-Dock7m +/+ Leprdb/J (db/db)	MG12	0, 2	Male/5 animals	Reduced blood sugar and HbA1c by 1% at 2 mg/kg
Steatohepatitis disease model efficacy test [3)]	C57BL/6J mouse	Vehicle MG12	0, 0.7, 2, 6	Male/5 animals	From 0.7 mg/kg, blood AST/ALT, liver weight, and TG content decrease.

1) NEGATIVE CONTROL GROUP AND MG12 TREATMENT GROUP EXTRACTION (REPORT NUMBER: MG12-PCP-RT-K008)

2) NEGATIVE CONTROL GROUP AND MG12 TREATMENT GROUP EXTRACTION (REPORT NUMBER: MG12-PCP-RT-K004)

3) NEGATIVE CONTROL GROUP AND MG12 TREATMENT GROUP EXTRACTION (REPORT NUMBER: MG12-PCP-RT-K003)

The toxicity studies for PG-102(MG12) were conducted using SD rats and cynomolgus monkeys in repeated-dose toxicity tests. A 4-week repeated-dose toxicity study was performed, in which PG-102(MG12) was administered subcutaneously once a week for a total of 5 doses over 4 weeks. Recovery groups were included to evaluate the reversibility of any observed toxic effects.

The results showed that the no-observed-adverse-effect level (NOAEL) for PG-102(MG12) was 12 mg/kg in SD rats and 6 mg/kg in cynomolgus monkeys, corresponding to a human equivalent dose (HED) of 1.94 mg/kg.

Title	Test system	Administered substance	Dosage (mg/kg)	Gender/Number of Animals	Key Results
Subcutaneous administration toxicity test repeated for 2 weeks (1 time/3-4 days, total 5 times)	SD rat	Vehicle MG12	0, 4, 12, 40	Male and female/5 each	Repeated toxicity test dosage determination: 40 mg/kg
Repeat for 4 weeks (1 time/week, total 5 times) 4-week recovery Subcutaneous administration toxicity test	SD rat	Vehicle MG12	0, 4, 12 40	Male and female/10 each [1)]	NOAEL: 12 mg/kg
Repeat for 4 weeks (1 time/week, total 5 times) 4-week recovery Subcutaneous administration toxicity test	Cynomolgus monkey	Vehicle MG12	0, 2, 6, 20	Male and female/3 each [2)]	NOAEL: 6 mg/kg

1) RECOVERY GROUP (CONTROL GROUP/HIGH DOSE GROUP) MALE AND FEMALE/5 ANIMALS EACH

2) RECOVERY GROUP (CONTROL GROUP/HIGH DOSE GROUP) MALE AND FEMALE/2 ANIMALS EACH

The safety pharmacology test of PG-102(MG12) evaluated its effects on the cardiovascular system in cynomolgus monkey and the central nervous system and respiratory system in SD rats.

Title	Test system (Gender/Number of animals)	Dosage (mg/kg)	Key Results
Cardiovascular system	Cynomolgus monkey (Male, 3 animals/group)	0, 0.6, 2, 6	In the 6 mg/kg group, an increase in heart rate and average blood pressure were observed, and a shortening of the QT interval related to the increase in heart rate was observed. There was no effect on the increase in QTc.<br>NOEL (No observed effect level): 2 mg/kg
Central nervous system	SD rat (Male, 3 animals/group)	0, 4, 12, 40	Reversible decrease in body temperature observed, recovery in 48 hours<br>No central nervous system effects observed up to 12 mg/kg, except for decrease in body temperature<br>Partial eyelid closure observed in the 40 mg/kg group

Title	Test system (Gender/Number of animals)	Dosage (mg/kg)	Key Results
Respiratory system	SD rat (Male, 8 animals/group)	0, 4, 12, 40	Decreased respiratory rate and minute volume,<br>No change in tidal breathing rate

During cardiovascular assessments, an increased heart rate and a consequent decrease in the QT interval were observed at the highest dose of 6 mg/kg; however, there was no effect on the corrected QT interval (QTc). These changes fully resolved within 12 hours post-administration.

Similar transient increases in arterial blood pressure and heart rate have also been reported with other GLP-1 receptor agonists (GLP-1 RAs) or GLP-2-based drugs sharing a similar pharmacological mechanism. Based on these findings, the observed cardiovascular effects are considered temporary responses.

Similar drugs		Test system	Administration	Summary of Results
Trulicity (Dulaglutide)	Safety Pharmacology	Cynomolgus monkey	Single, SC	1 mg/kg, 10 mg/kg<br>Increased heart rate, observe changes in ECG
	Repeated dose toxicity (3 months)	Cynomolgus monkey	Repeat, S.C.	8.15 mg/kg observed heart rate increase
Mounjaro (Tirzepatide)	Safety Pharmacology	Cynomolgus monkey	Single, SC	0.15 mg/kg blood increase, pulse pressure decrease, heart rate increase<br>0.50 mg/kg increased heart rate
	Repeated dose toxicity (1, 3 months)	Cynomolgus monkey	Repeat, S.C.	0.05, 0.15 or 0.5 mg/kg administration group high heart rate
Saxenda (Liraglutide)	Stability Pharmacology	Rat	Single, SC	0.2 and 2 mg/kg dose groups<br>Increased heart rate and increased arterial blood pressure
Gattex (Teduglutide)	Stability Pharmacology	Beagle dog	IV	Low dose group<br>Increased heart rate, increased maximum ventricular contractility

In central nervous system (CNS) evaluations, a dose-dependent decrease in average body temperature was observed across all dose levels. However, body temperature began to recover between 12 and 24 hours post-administration and returned to baseline by 48 hours. Individual body temperatures remained within the range of 35℃ to 37℃.

Similar decreases in body temperature have also been reported with GLP-1 receptor agonists (GLP-1 RAs) that share a comparable pharmacological mechanism. These findings suggest that the temperature reduction is a transient and expected response.

Similar drugs		Test system	Administration	Summary of Results
Trulicity (Dulaglutide)	Repeated dose toxicity (1 month)	Cynomolgus monkey	2 times a week, 9 times in total	3 mg/kg administration group<br>Observe a decrease in body temperature
Saxenda (Liraglutide)	Safety Pharmacology	rat	one time	0.2 mg/kg and 2 mg/kg administration groups<br>Observe a decrease in body temperature
Ozempic (Semaglutide)	carcinogenicity test	CD-1 mouse	everyday	0.1 mg/kg to 3 mg/kg<br>Observe decreased body temperature and decreased activity

In the PG-102(MG12) treatment group, a decrease in respiratory rate (breaths per minute) was observed after drug administration, along with a reduction in minute ventilation due to the lower respiratory rate. However, no decrease in tidal volume or other respiratory-related changes were noted.

Most subjects showed recovery from these changes within 72 hours, and individual respiratory rates were largely within the normal range of 75 to 129 breaths per minute. Therefore, the decrease in respiratory rate and minute ventilation is considered a reversible response, and no impairment of respiratory function due to the drug's action is expected.

3.2.2. Rationale for Dose Selection in Clinical Design

The maximum single dose for this clinical trial was set at less than 50% of the projected human exposure to PG-102(MG12) based on the NOAEL value of 6 mg/kg from nonclinical toxicity studies, as determined through pharmacokinetic (PK) modeling.

The PK modeling applied a two-compartment model based on nonclinical data from monkeys, a species with high relevance to human physiology. Linear PK was assumed over a dose range of 2–20 mg/kg, and all available data from monkey toxicokinetic (TK) studies were utilized for monkey PK modeling.

Human PK simulations were conducted using PK parameters derived from monkey PK modeling, scaled up using allometric principles. The dosing intervals were set to weekly (Simulation h2, QW) or biweekly (Simulation h-2-1, Q2W). Doses ranging from 1 to 90 mg/dose were modeled as single or 5-weekly repeated subcutaneous (SC) administrations to predict PG-102(MG12) exposure in humans.

The maximum planned dose for humans corresponds to a 50% exposure level of the NOAEL in the monkey repeated-dose toxicity study (equivalent to 87 mg/dose in humans). To enhance safety, the maximum planned dose was reduced by 30% to 60 mg/dose. Based on the safety and tolerability results observed at 60 mg/dose, an optional cohort with dose escalation up to 80 mg/dose was included, making 80 mg/dose the maximum dose for this study.

Criteria	NOAEL exposures			Maximum planned clinical dose in humans (mg)	
		QW	Q2W	QW	Q2W
	$C_{max,ss}$ (ng/mL)	$AUC_{\tau,ss}$ (ng h/mL)	$AUC_{\tau,ss} \times 2$ (ng h/mL)	#h2	#h2-1

100% exposures	20450	1595000	3190000	174	348
50% exposures	10225	797500	1595000	87	174

In nonclinical repeated-dose toxicity studies of PG-102(MG12) conducted in SD rats (2-week or 4-week total of 5 doses) and cynomolgus monkeys (4-week total of 5 doses), the no-observed-adverse-effect level (NOAEL) was determined to be 12 mg/kg in SD rats and 6 mg/kg in monkeys. When converted to a human equivalent dose (HED), this corresponds to 1.94 mg/kg. Based on a safety factor of 10, the maximum recommended starting dose (MRSD) for clinical trials was calculated to be 0.194 mg/kg.

Considering the efficacy and toxicity data, the initial dose for clinical trials was set at 0.081 mg/kg (5 mg/dose, corresponding to 4.88–5.69 mg/dose for individuals weighing 60–70 kg). The first-in-human (FIH) clinical trial was designed with this starting dose.

For Part A (single-dose escalation), the planned doses included 5 mg/dose, 15 mg/dose, 30 mg/dose, and 60 mg/dose, with an optional cohort at 80 mg/dose. The 80 mg/dose (optional) cohort will be conducted based on a comprehensive evaluation of tolerability and safety data from the planned dose cohorts.

As of July 10, 2024, Cohort A4 (60 mg/dose) of Part A was completed, and a safe and tolerable dose of 15 mg/dose was selected for repeated dosing in Part B. Cohort B1 (15 mg/dose) has been completed, and Cohort B2 (30 mg/dose) has completed drug administration.

To date, no serious adverse events have been reported.

3.3. Benefit/Risk Assessment

This trial is a clinical study involving healthy adult and obese subjects, and there are no direct therapeutic or health benefits expected from participation in this trial. The study has experimental aspects that are unverified, and even healthy adults may experience adverse events. In addition to the predictable gastrointestinal adverse events commonly reported with GLP-1 receptor agonists, such as nausea, diarrhea, constipation, and vomiting, there may be rare, unforeseen adverse events.

The information gathered from this trial could potentially be used in the development of better treatments in the future. The investigator (or designated sub-investigator) will thoroughly explain these aspects to the subjects, and the trial will only proceed with subjects who understand and voluntarily agree to participate.

4. Objectives

This clinical trial aims to evaluate the safety, tolerability, pharmacokinetic, and pharmacodynamic properties of subcutaneous administration of PG-102(MG12) in healthy adult subjects and obese subjects.

4.1. Primary Objective

The primary objective of this clinical trial is to evaluate the safety and tolerability of PG-102(MG12) after [redacted] repeated administration (Parts B and C) in healthy adult and obese subjects, by assessing the occurrence of adverse events.

4.2. Secondary Objective

The secondary objective of this clinical trial is to evaluate the pharmacokinetic and pharmacodynamic properties of PG-102(MG12) after [redacted] repeated administration (Parts B [redacted]) in healthy adult [redacted]

4.3. Exploratory Objective

For [Part B] [redacted] the exploratory objective is to evaluate the inflammatory-related biomarkers of PG-102(MG12).

5. Study Design

5.1. Overall Design

This clinical trial is a randomized, placebo-controlled, double-blind, single (Part A) and multiple (Parts B and C) ascending dose Phase 1 study.

5.1.1. [Part A] PG-102(MG12) Single Dose Administration

[redacted]

[redacted]

[redacted]

[redacted]

[redacted]

[redacted]

[redacted]

5.1.2. [Part B] and [Part C] PG-102(MG12) Multiple Dose Administration

[Part B] will be conducted as a randomized, double-blind, placebo-controlled, 5-dose repeated subcutaneous administration, dose-escalation study in healthy adult subjects. The starting dose for Part B (15 mg/dose) was determined based on the safety and tolerability data collected from the single-dose administration in Part A. The study will include up to 4 cohorts (including the optional cohort), with each cohort comprising 6 subjects receiving the investigational drug (PG-102 [MG12]) and 2 subjects receiving placebo (0.9% normal saline for injection), administered subcutaneously in 5 repeated doses.

For Cohort S (optional), the investigational drug (PG-102 [MG12]) at the optimal dose and placebo (0.9% normal saline for injection) will be administered subcutaneously at 2-week intervals for a total of 5 doses.

The dose-escalation process for the cohorts is as follows: Cohort B1 will proceed based on the safety data from Cohort A3; Cohort B2 will proceed based on the safety data from Cohort A4 and Cohort B1; Cohort B3 will proceed based on the safety data from Cohort A4 and Cohort B2.

[redacted]

[redacted]

[redacted]

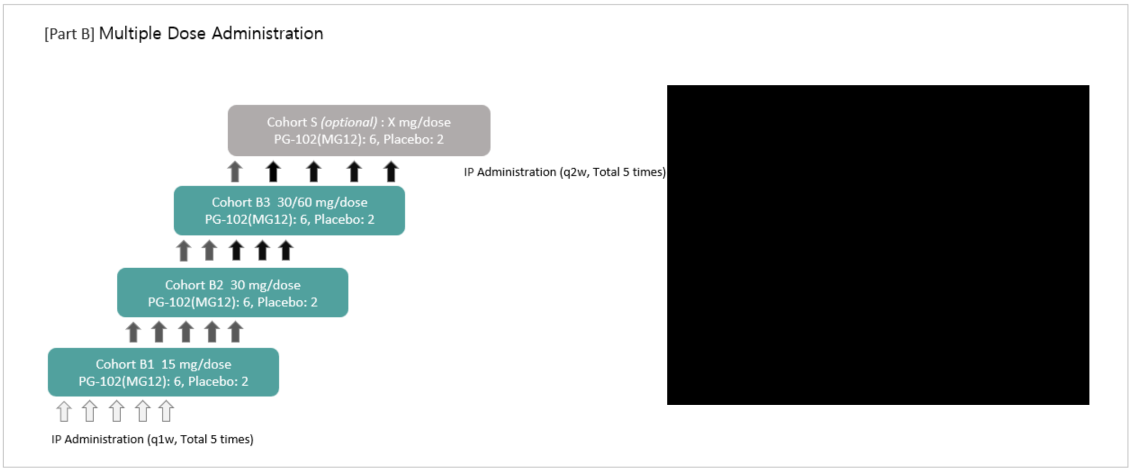

[Figure 5] PG-102(MG12) Multiple ascending dose

[Table 1] Dosing Regimen and Dosage for Cohorts B1–B3 and Cohorts C1, C2

	1st	2nd	3rd	4th	5th
**Cohort B1**	15 mg	15 mg	15 mg	15 mg	15 mg
**Cohort B2**	30 mg	30 mg	30 mg	30 mg	30 mg
**Cohort B3**	30 mg	30 mg	60 mg	60 mg	60 mg
[redacted]	[redacted]	[redacted]	[redacted]	[redacted]	[redacted]
[redacted]	[redacted]	[redacted]	[redacted]	[redacted]	[redacted]

5.2. Overview of Study Periods

The total duration of the clinical trial is approximately 24 months following the approval of the clinical trial protocol by the Institutional Review Board (IRB), and it may be subject to change depending on the enrollment rate of subjects. The study period for each subject consists of the following evaluation schedule.

- Screening Period: 4 weeks
- Treatment Period

  [redacted]

  [Part B] 6 weeks (weekly administration) or 10 weeks (biweekly administration for cohort S, if conducted as optional)

  [redacted]
- Follow-up Period: [redacted] [Part B] 2 weeks [redacted]

5.3. Scientific Rationale for Study Design

5.3.1. Theoretical Rationale for the Endpoints

5.3.1.1. Safety Endpoints

During the clinical trial, the following items will be evaluated to assess the safety and tolerability of the investigational medicinal product after its administration:

- Clinical Safety: Adverse events, previous drug history and concomitant medications, physical examination, vital signs, electrocardiogram (12-lead ECG), immunogenicity assessment (ADA)
- Laboratory Tests: Hematology tests, blood chemistry tests, coagulation tests, urinalysis, serological tests

5.3.1.2. Pharmacokinetic Endpoints

In this clinical trial, pharmacokinetic (PK) evaluations will be performed after single-dose and repeated-dose administration of PG-102(MG12), and the following variables will be assessed:

$AUC_{0\text{-}t}$	The area under the drug concentration-time curve calculated using linear trapezoidal summation from time 0 to time t_{last}, where t_{last} is the time of the last measurable concentration (Ct).
$AUC_{0\text{-}inf}$	The area under the drug concentration-time curve from time 0 to infinity, , AUC0-inf = AUC0-t + Ct/λz, where λz is the apparent terminal elimination rate constant
C_{max}	Maximum drug concentration
λz	Terminal elimination rate constant

$t_{1/2}$	Terminal half-life
t_{max}	Time to reach maximum concentration
CL/F	Apparent total clearance calculated by dose/$AUC_{0\text{-}inf}$
Vz/F	Apparent volume of distribution in the terminal phase calculated by (CL/F)/λz
$C_{max,ss}$	Steady-state maximum plasma concentration
$T_{max,ss}$	Time to reach maximum plasma concentration at steady state
AUC_{tau}	Area under the concentration-time curve over the dosing interval (0 to τ)
AUC_{inf}	Area under the concentration-time curve from time 0 to infinity
$C_{avg,ss}$	Average plasma concentration at steady state
$C_{trough,ss}$	Trough plasma concentration at steady state

5.3.1.3. Pharmacodynamic Evaluation and Biomarker Assessment

PG-102(MG12), a dual-specific Fc fusion protein of GLP-1/GLP-2, will be evaluated in this clinical trial considering its pharmacological mechanisms (blood glucose control, metabolic function improvement, weight loss, improvement in intestinal leakage, and modulation of gut microbiome). The pharmacodynamic endpoints of this study include the evaluation of hemoglobin A1c (HbA1c) levels, oral glucose tolerance test (OGTT), weight control indicators, inflammatory biomarkers, and gut microbiome. Weight control indicators will be assessed according to the "Clinical Trial Guidelines for Anti-Obesity Drugs," measuring changes in body weight, waist circumference, waist-to-hip ratio, and body fat (DEXA), as well as evaluating the inflammatory biomarker plasma hsCRP.

5.3.2. Theoretical Rationale for the Use of Placebo

This clinical trial is a Phase 1 study to evaluate the safety and tolerability of a single or repeated dose of PG-102(MG12) in healthy adults or obese subjects, with a placebo control group (administered 0.9% saline solution) included.

5.4. Rationale for Dose Selection and Dose Escalation Criteria (Part A)

5.4.1. Rationale for Starting Dose Selection

In the non-clinical repeated-dose toxicity study conducted in SD rats and cynomolgus monkeys (rats: 2 weeks or 4 weeks with a total of 5 doses, cynomolgus monkeys: 4 weeks with a total of 5 doses), the maximum no observed adverse effect level (NOAEL) of PG-102(MG12) was determined to be 12 mg/kg in rats and 6 mg/kg in cynomolgus monkeys. When converted to human equivalent doses, this corresponds to approximately 1.94 mg/kg in both species, and the maximum recommended starting dose (MRSD), considering a safety factor of 10, is estimated to be around 0.194 mg/kg.

The human equivalent dose for the effective dose based on the non-clinical efficacy studies is estimated to be 0.081 mg/kg. Therefore, the starting dose for the clinical trial (First In Human, FIH) is set at a lower dose of 0.081 mg/kg (5 mg/dose, based on a 60-70 kg body weight: 4.88 - 5.69 mg/dose).

5.4.2. Dose Escalation and Discontinuation Criteria

The dose escalation for the single-dose administration (Part A) in this clinical trial will begin with a dose of 5 mg/dose, which corresponds to one-tenth of the NOAEL from the non-clinical repeated-dose toxicity study. The dose will then be increased in three steps, with each subsequent dose being three times higher, followed by further increases of two times the previous dose, reaching a total of four dose levels up to 60 mg/dose. Afterward, the 80

mg/dose will be an optional cohort, and it can proceed only after reviewing the safety and tolerability data for 60 mg/dose.

The maximum dose of 80 mg/dose, set for this clinical trial, was determined using pharmacokinetic (PK) and pharmacodynamic (PD) modeling and simulation with an allometric scale-up approach. The PK/PD modeling and simulations were conducted based on the PG-102(MG12) non-clinical toxicity and toxicokinetic (TK) study data obtained from primates. In those non-clinical studies, PG-102(MG12) was administered at escalating doses in 3-fold increments, and dose-dependent plasma exposure and efficacy/toxicity responses were observed, with the maximum no-observed-adverse-effect level (NOAEL) confirmed at 6 mg/kg.

The pharmacometric PK simulation results suggest that administering 87 mg/dose in humans would produce an exposure level approximately 50% of the NOAEL observed in the primate non-clinical toxicity studies. Based on this, dose escalation will proceed up to 60 mg/dose in Part A, and up to 80 mg/dose in the optional cohort will be considered. Dose escalation and stopping criteria for each cohort will be based on the guidelines specified in Section 8.5 of the protocol.

5.5. Rationale for Dose Selection and Dose Escalation Criteria (Part B [redacted])

5.5.1. Rationale for Starting Dose Selection

[redacted]

Additionally, based on pharmacometric PK simulation results (refer to Table 2 in Section 5.5.3), it was predicted that when 15 mg is administered for 5 repeated doses, the PK parameters (Cmax and AUC) would increase by only about 15% compared to a single dose. Therefore, repeated dosing with 15 mg/dose was initiated in Cohort B1 of Part B.

5.5.2. Theoretical Rationale for the Study Design

The representative adverse events for GLP-1 receptor agonists, similar to the investigational medicinal product in this clinical trial, are gastrointestinal-related adverse events. These events are known to be reduced in incidence and to improve patient adherence by starting at a low dose and gradually increasing to the target dose with specific intervals (Marx et al., 2022).

Additionally, based on the pharmacometric PK simulation results, summarized in Table 1 of Section 5.1.2 (refer to Table 2 and Figure 5 in Section 5.5.3), the PK parameters (Cmax and AUC) for 4 and 5 doses were found to be similar, suggesting that the dosing regimen outlined in Table 1 would likely reach steady state.

Therefore, for the cohorts in Part B, except for Cohort B1, the plan is to increase the dose within each cohort to the target dose.

5.5.3. Theoretical Rationale for Dosing Interval

This clinical trial is primarily designed to assess the safety and tolerability of PG-102(MG12). The dosing interval for repeated administration (Part B) has been set to once a week, based on the results of nonclinical toxicity studies, TK (toxicokinetic) tests, and pharmacometric PK simulation results.

[Table 2] Pharmacometric PK Simulation Results for Repeated Dosing

	1st treatment		2nd treatment		3rd treatment		4th treatment		5th treatment	
	C_{max} (ng/mL)	AUC_{168} (ng*h/mL)	C_{max} (ng/mL)	AUC_{168} (ng*h/mL)	C_{max} (ng/mL)	AUC_{168} (ng*h/mL)	C_{max} (ng/mL)	AUC_{168} (ng*h/mL)	$C_{max,ss}$ (ng/mL)	$AUC_{t,ss}$ (ng*h/mL)
Cohort B1	1177	120605	1352	136620	1366	137858	1367	137948	1367	137954
Cohort B2	2354	241209	2704	273240	2732	275716	2734	275896	2734	275909
Cohort B3	2354	241209	2704	273240	5083	516925	5438	549136	5467	551625

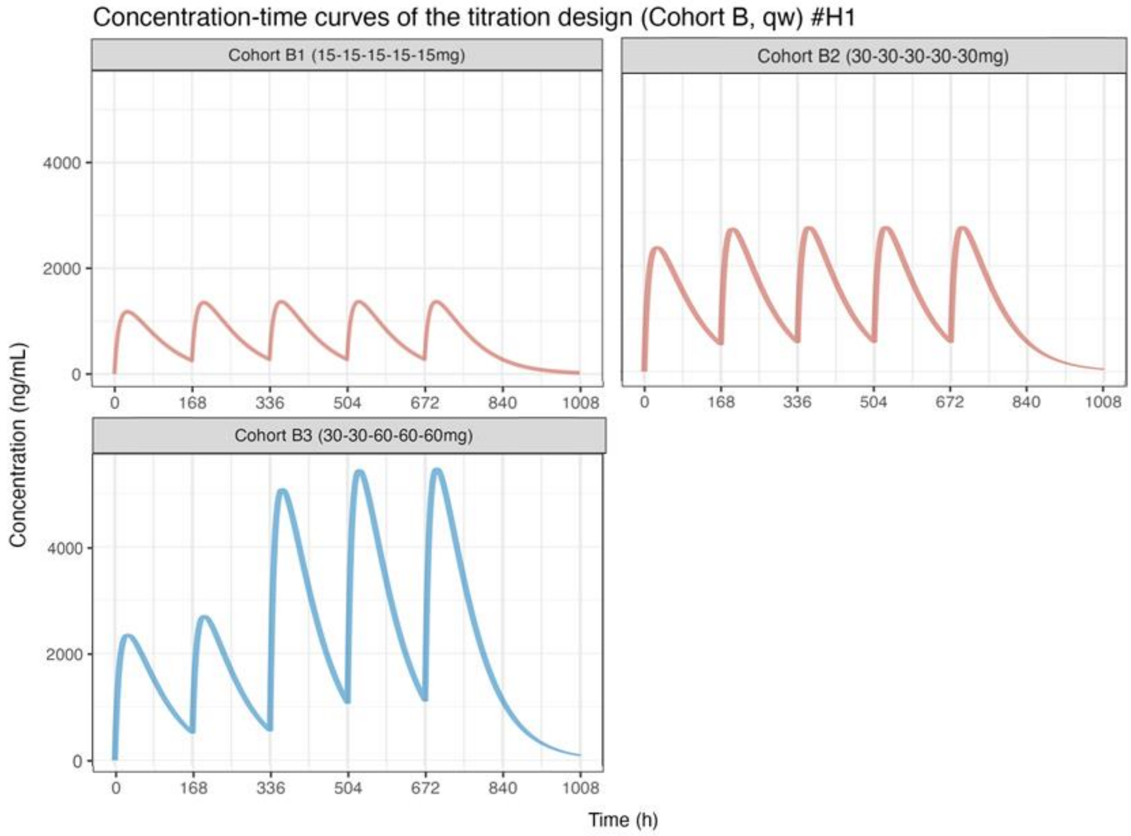

[Figure 6] Pharmacometric PK Simulation Results for Repeated Dosing in Part B

Additionally, an optional Cohort S was added, in which PG-102(MG12) or a placebo is administered once every two weeks (q2w) for a total of five doses. This cohort was introduced based on the safety and tolerability data collected during the clinical trial to determine the optimal dose.

5.5.4. Basis for Dose Selection in Part C

[redacted]

[Table 3] Anticipated Drug Exposure Based on Body Mass Index (BMI) for Cohorts B3, C1, and C2

	[redacted]		[redacted]
	[redacted]	[redacted]	[redacted]
[redacted]	[redacted]	[redacted]	[redacted]
[redacted]	[redacted]	[redacted]	[redacted]
[redacted]	[redacted]	[redacted]	[redacted]

[redacted]

2) Reflecting BMI 30 kg/m^2 compared to BMI 25 kg/m^2

[redacted]

6. Study Population

6.1. Number of Subjects

[redacted]

Part B will include a total of 32 subjects, with 8 subjects per dose group (6 receiving PG-102(MG12) and 2 receiving placebo) across 4 dose groups (including optional Cohort S).

[redacted]

6.2. Inclusion Criteria

The subjects **must satisfy all of the following eligibility criteria**

1) Male or female participants, aged 18 to 65 years inclusive at the time of signing informed consent
2) Volunteers who have received a detailed explanation of the clinical trial, fully understand it, voluntarily decide to participate, and provide written informed consent
3) Subjects who agree to abstain from alcohol consumption from 48 hours prior to screening and administration of the investigational product, and throughout the hospitalization period until discharge
4) Subjects without congenital or chronic diseases within the past 5 years and with no abnormal findings based on a medical examination
5) Subjects with a body weight of ≥ 55 kg for males and ≥ 50 kg for females at screening, and who meet the following body mass index (BMI) criteria

   [redacted]

   [Part B]: Subjects with a body mass index (BMI) of ≥ 25 kg/m² and < 30 kg/m² at the time of screening

   [redacted]

6) Subjects deemed eligible based on health assessments performed during screening, including medical history, physical examination, vital signs, 12-lead electrocardiogram (ECG), and laboratory tests

6.3. Exclusion Criteria

Subjects who **meet any of the following criteria are not eligible to participate in this clinical trial**

1) Subjects who have taken prescription drugs, herbal medicines, over-the-counter drugs, or vitamin supplements within 10 days prior to the first administration of the investigational product, or who have used the following drugs and/or other substances within 90 days prior to screening
   - Drugs that affect body weight (such as obesity medications, psychiatric drugs, beta blockers, diuretics, contraceptives, female hormones, proton-pump inhibitors (PPI), H2 receptor antagonists, health functional foods/supplements, and formulas designed for weight control)

- Drugs that have the potential to impact blood sugar, liver fat, and intestinal microorganisms (including GLP-1 receptor agonists, DPP-4 inhibitors, SGLT-2 inhibitors, thiazolidinedione (TZDs), fish oil, polyunsaturated fatty acids (PUFA), and ursodeoxycholic acid (UDCA)), as well as individuals who are currently using insulin

  [redacted]

2) Subjects who have participated in other clinical trials or bioequivalence studies and received investigational products within 180 days prior to the first administration of the investigational product
3) Subjects who have donated whole blood within 60 days prior to the first administration of the investigational product, donated blood components within 30 days prior, or received a blood transfusion within 30 days prior
4) Subjects who do not agree to use dual contraception methods** or to practice abstinence from the time of signing the informed consent form until 90 days after the last administration of the investigational product
**Dual contraception methods: intrauterine devices (IUDs), chemical barriers (e.g., spermicides), physical barriers (e.g., male or female condoms), tubal ligation or laparoscopic sterilization, vasectomy, or strict abstinence
5) Subjects with a history of clinically significant diseases affecting the cardiovascular, respiratory, renal, endocrine, hematologic, gastrointestinal, central nervous, urogenital, musculoskeletal, or psychiatric systems, or malignancies, or those with active diseases in these systems (except in cases where the condition has been completely cured and does not affect the current health status)
6) Subjects with a history of gastrointestinal diseases that may affect the absorption of the investigational product (e.g., Crohn's disease, ulcers, acute or chronic pancreatitis) or a history of gastrointestinal surgery (excluding simple appendectomy or hernia repair)
7) Subjects with a history of acute proliferative retinopathy or maculopathy, severe gastroparesis, and/or severe neruopathy
8) Subjects with a history of surgical treatment for obesity within 2 years (example: bariatric surgery, gastric banding etc) or gastrointestinal procedures for weight loss (including LAP-BAND®), or uncontrolled gastrointestinal disorders at Screening (e.g., peptic ulcer, gastroesophageal reflux disease)
9) Subjects with a history of drug abuse
10) Subjects who are unable to abstain from consuming caffeine-containing foods and beverages (e.g., coffee, tea [black tea, green tea, etc.], caffeinated soft drinks, coffee-flavored milk, energy drinks) or from smoking during the period from 9:00 AM on the day of hospitalization until discharge
11) Subjects who excessively consume caffeine (> 5 cups/day) or alcohol (> 210 g/week) or are heavy smokers (> 10 cigerettes/day)
12) Subjects with a known allergy to GLP-1 or GLP-2 receptor agonists
13) Subjects with a history of hypersensitivity or clinically significant allergic reactions to investigational products, other drugs, or excipients
14) Subjects who have received intravenous administration of radiopaque iodine contrast agents (e.g.,

Intravenous urography, angiography, intravenous cholangiography, or contrast-enhanced computed tomography) within 48 hours prior to the first administration of the investigational product

15) Subjects who, after resting for at least 5 minutes in a sitting position at screening, have systolic blood pressure ≥ 150 mmHg or < 90 mmHg, or diastolic blood pressure ≥ 100 mmHg or < 50 mmHg

16) Subjects who have laboratory test results meeting any of the following criteria:
   - Positive results for serum tests (HBsAg, HCV Ab, HIV Ag/Ab, Syphilis reagin test)
   - eGFR (estimated Glomerular Filtration Rate) < 60 mL/min/1.73 m^2 based on the CKD-EPI (Chronic Kidney Disease Epidemiology Collaboration) formula
   - Serum creatinine levels ≥ 1.5 mg/dL in males, ≥ 1.4 mg/dL in females, or creatinine clearance (CrCL) < 50 mL/min in all subjects

17) Subjects with significantly abnormal liver function tests or those meeting any of the following conditions
   - Serum AST or ALT > 1.25 times the upper limit of normal

     [redacted]
   - Serum total bilirubin > 1.5 times the upper limit of normal

18) Subjects whom the investigator deems unsuitable for participation in the clinical trial for any other reason

6.4. Lifestyle Considerations

6.4.1. Meal and Dietary Restrictions

The subjects' diet and dietary restrictions will follow the guidelines specified in Section 9.4, "Lifestyle and Dietary Restrictions."

6.4.2. Activity Restrictions

No special restrictions on activity are required during the clinical trial period. However, subjects are advised to maintain a regular level of physical activity/exercise after receiving the clinical trial medication.

6.4.3. Screen Failures

Screening failure is defined as when a subject agrees to participate in the clinical trial but is not assigned a subject number. A subject who fails screening may undergo rescreening once, and in this case, a new screening number will be assigned to the subject.

6.4.4. Substitution of Study Subjects

If a subject drops out of the trial early, no replacement will be made. However, if a subject has been assigned a subject number but withdraws consent before receiving the dose, or if they are unable to proceed with dosing due to the results of D-1 screening tests, a qualified subject from the pool of screened candidates may be substituted.

7. Overview and management of clinical investigational medicinal products

7.1. Study Intervention

7.1.1. PG-102(MG12)

PG-102(MG12) finished pharmaceutical product is a colorless, transparent injectable solution. The active ingredient content of the PG-102(MG12) finished pharmaceutical product is manufactured in concentrations of 6 mg/mL and 50 mg/mL, respectively. The composition is as shown in Table 1. Each vial of PG-102(MG12) 6 mg/mL contains 1.0 mL, and each vial of PG-102(MG12) 50 mg/mL contains 0.6 mL.

[Table 4] Composition of PG-102(MG12) Finished Pharmaceutical Product 6 mg/mL and 50 mg/mL

Purpose	Ingredients	Specification	Amount (1 mL)	
			MG12 6 mg/mL	MG12 50 mg/mL
Active Ingredient	PG-102(MG12)	In-house specification	6.00 mg	50.00 mg
Buffer	Sodium Dihydrogen Phosphate Dihydrate	Ph. Eur.	2.44 mg	2.44 mg
Buffer	Disodium Hydrogen Phosphate Dihydrate	Ph. Eur.	0.98 mg	0.98 mg
Stabilizer	Sucrose	Ph. Eur.	30.00 mg	30.00 mg
Stabilizer	Glycine	Ph. Eur.	3.75 mg	3.75 mg
Stabilizer	L-Histidine	Ph. Eur.	7.75 mg	7.75 mg
Stabilizer	L-Methionine	Ph. Eur.	7.46 mg	7.46 mg
Stabilizer	Polysorbate 80	Ph. Eur.	0.50 mg	0.50 mg
Solvent	Water for injection (WFI)	Ph. Eur.	q.s. 1 mL	q.s. 1 mL

1) In-house specification
2) Ph. Eur, European Pharmacopeia
3) q.s., quantum sufficit

7.1.2. Placebo

In this clinical trial, the placebo information is as follows, and a product approved by the Korean Ministry of Food and Drug Safety (MFDS) will be used. Relevant information will be provided in the investigational medicinal product manual.

Items	Product name or ingredients	Storage condition
Placebo	0.9% saline solution for injection	Sealed container, store at room temperature (1-30℃)

7.2. Handling, Storage, and Inventory Management

The labeling requirements for investigational medicinal products (IMPs) shall follow the "Regulations on the Manufacture and Quality Control of Pharmaceutical Products," and detailed information can be found in the investigational medicinal product manual. IMPs must be stored in a separate location with a locking device at the clinical trial site, and access is restricted to authorized individuals (the managing pharmacist or those designated by the clinical trial principal investigator, hereafter referred to as the "managing pharmacist"). The managing

pharmacist at the clinical trial site must receive a receipt for the IMPs and store them properly, ensuring that the IMPs are maintained at the appropriate temperature in suitable storage conditions. Furthermore, the managing pharmacist must ensure that the IMPs are not used for purposes other than the clinical trial.

The sponsor is responsible for verifying the quantity and storage conditions of the IMPs during the clinical trial and taking necessary actions to ensure the trial is conducted appropriately. Additionally, the sponsor must ensure that unused IMPs are retrieved and disposed of when the clinical trial is discontinued or concluded. Used vials can be disposed of according to the regulations of the clinical trial site.

7.3. Preparation and Disbursement

To minimize exposure to investigational medicinal products (IMPs), a qualified person in charge of IMP management must perform preparation, dispensing, and safe disposal. If it is anticipated that there may be difficulties in administering the IMP to subjects due to the operating hours of the clinical pharmacy, the responsibility for IMP management may be temporarily delegated to the principal investigator or a designated blinded trial staff, based on prior agreement. Detailed instructions for the preparation and dispensing of IMPs are provided in the investigational medicinal product manual.

8. Administration of Investigational Medicinal Product

8.1. PG-102(MG12)

PG-102(MG12) is administered as a subcutaneous injection in the abdomen slowly, according to the dosing regimen for each cohort: [redacted], [Part B] involves weekly dosing (with Cohort S (optional group) receiving a dose every two weeks), for a total of 5 doses. [redacted] administered slowly subcutaneously in the abdomen.

Dosage	Site of Administration	Method of Administration
Less than or equal to 1.0 mL	Abdomen<br>(5 cm away from the navel)	Slow subcutaneous injection on one side
Greater than 1.0 mL	Abdomen<br>(5 cm away from the navel)	Slow subcutaneous injection on both sides<br>(avoiding the same site)

8.2. Placebo

The placebo will be administered with the same frequency and dosing schedule as PG-102(MG12) in [redacted] [Part B][redacted]

8.3. Randomization

The randomization codes for trial subjects who meet the inclusion/exclusion criteria will be generated by the person designated by the sponsor to create the randomization codes. The randomization ratio will be [redacted] 3:1 (Part B), [redacted] for the experimental group and the placebo group, respectively. The randomization codes will be securely stored until the blinding is broken. The emergency unblinding envelope containing the randomization code will be provided to the principal investigator or a designated sub-investigator.

8.4. Blinding and Unblinding

This clinical trial is a double-blind study, where both the investigator and the subject remain blinded. To maintain blinding, the preparation of the investigational medicinal product (e.g., dilution and syringe/vial filling) will be carried out by the managing pharmacist or a person whose blinding has been broken. The drug preparation should be done in a separate space, and the filled syringes (or vials) will be provided to the investigator without any information about the specific drug, ensuring that blinding is maintained. Both the subject and the investigator will remain blinded to avoid any bias when the subject describes improvements or adverse events and when the investigator assesses efficacy.

The allocation information for clinical trial subjects and investigational medicinal products will remain blinded to everyone except for the randomization code generator, the managing pharmacist, or the person responsible for the investigational medicinal products whose blinding has been broken. Blinding will not be broken before the final statistical analysis, unless unblinding is required for urgent safety concerns, such as a serious adverse event, or for an interim analysis.

The double-blind status may be broken in situations where the investigator needs to know the investigational medicinal product information for clinical management of the trial subject. If a reported serious adverse event is an unexpected adverse event related to the investigational medicinal product, the principal investigator may decide, in consultation with the sponsor, to unblind the allocation information for the specific subject. Once the unblinding envelope is opened, the principal investigator must inform the sponsor of the unblinding. The date and reason for unblinding of the subject must be documented in the Blinding Decoding Log, and the information must also be detailed in the source documents and case report forms.

8.5. Dose Adjustment

The dose escalation of PG-102(MG12) will be determined after reviewing the safety and tolerability data collected up to 14 days post-dose for each cohort. The safety information and the decision regarding dose escalation will be reviewed and determined by the Safety Review Committee (SRC) each time the dose is increased.

The SRC will conduct the review within 4 weeks from the last dose administration of the final subject in each dose cohort. The review will be based on adverse event monitoring, vital signs, physical examinations, laboratory tests (hematological/biochemical/coagulation/urine tests), and electrocardiogram (ECG) results collected up to 14 days post-dose of the investigational medicinal product or placebo. The decision to escalate the dose will be made according to the dose escalation criteria outlined in section 8.5.2. If the majority of the attending SRC members approve, the next dose level will be initiated. [redacted]

8.5.1. Dose Escalation and Repeated Administration

8.5.1.1. [Part A] PG-102(MG12) Single Dose Administration

[redacted]

[redacted]

8.5.1.2. [Part B] PG-102(MG12) Multiple Dose Administration

In [Part B], subcutaneous injections will be administered once weekly (q1w) for a total of 5 doses. For Cohort S (optional), subcutaneous injections will be administered every two weeks (q2w) for a total of 5 doses.

In [Part B], dose escalation will proceed as follows: Cohort B1 will advance based on the safety data from Cohort A3; Cohort B2 will advance based on the safety data from Cohort A4 and Cohort B1; and Cohort B3 will advance based on the safety data from Cohort A4 and Cohort B2.

The safety data for each cohort will consist of safety and tolerability information collected up to 14 days after the final administration of the investigational medicinal product in all subjects of the cohort. These data will be reviewed by the Safety Review Committee (SRC) to confirm the absence of any safety concerns before initiating the administration of the investigational drug at the next cohort's dose level.

8.5.1.3. [Part C] PG-102(MG12) Multiple Dose Administration

[redacted]

[redacted]

[redacted]

8.5.2. Criteria for Dose Escalation and Repeated Administration Discontinuation

The dose escalation and/or repeated dosing of the investigational medicinal product will be temporarily suspended in the following cases after administration. The severity of drug-related adverse events will be evaluated according to the severity grading criteria in section 11.1.2.1. The decision to resume dosing or permanently discontinue dosing will be made following a review by the Safety Review Committee (SRC), with the blinding maintained.

[Table 5] Criteria for Stopping Dose Escalation of Investigational medicinal product(Adverse Event Incidence Rate%)

Severity of adverse events related to Investigational medicinal product	Incidence rate (%)
Moderate adverse event*	50%
Severe adverse event	25%
Serious adverse event	1 or more people

* Only drug-related moderate or severe adverse events that do not resolve with outpatient treatment (i.e., treatment not requiring hospitalization) will be considered in the following situations.

8.6. Concomitant medication and treatment

8.6.1. Concomitant Medications and Treatments

Medications that do not affect the safety or pharmacokinetic properties of the investigational medicinal product may be allowed at the investigator's discretion.

[redacted]

For all concomitant medications (including treatment for other conditions or adverse events), detailed information about the medication (e.g., product name or active ingredient, purpose, dosage, and duration of administration) must be recorded in the case report form. Concomitant use of medications should be minimized during the study, except for essential medications.

8.6.2. Concomitant Medications and Treatments

The following medications or treatments are prohibited during the clinical trial, including the follow-up period:

- Weight-affecting medications (e.g., obesity treatments, psychiatric medications, beta-blockers, diuretics, contraceptives, hormone therapies, proton-pump inhibitors (PPIs), H2 receptor antagonists, weight control health supplements, and formulated foods).
- Medications that may affect blood sugar, liver fat, or gut microbiome (e.g., GLP-1 receptor agonists, DPP-4 inhibitors, SGLT-2 inhibitors, thiazolidinediones (TZDs), fish oil, polyunsaturated fatty acids (PUFAs), ursodeoxycholic acid (UDCA), insulin).

However, for subjects in [Part C], stable use of antihypertensive or antihyperlipidemic medications is allowed.

At the investigator's discretion, if the use of a prohibited concomitant medication is necessary for the treatment of the subject during the clinical trial, the investigator must evaluate whether the subject can continue participating in the trial and record this decision in the case report form with detailed information.

9. Clinical Trial Procedures and Evaluation

9.1. General Procedures

9.1.1. Subject Consent and Screening Number Assignment

The principal investigator or a designated sub-investigator must provide the subject (or the subject's legal representative) with a detailed explanation of the purpose and procedures of the clinical trial before enrollment and obtain written consent. The written consent must be obtained before any clinical trial procedures are performed. The investigator must provide the subject with a copy of the signed consent form.

After obtaining written consent, the investigator assigns a screening number to the subject in the order in which the consent forms were received. The initials and screening number assigned to each subject will be used as the subject identification code.

Example: S-001
- ‘S’: the first letter of "Screening"
- ‘001’: Sereal number

The screening number of subjects who are excluded during screening will not be reassigned to other subjects. If a subject is excluded during screening, one re-screening is allowed. A new screening number will be assigned to the subject undergoing re-screening, and the original screening number will not be reused.

9.1.2. Collection of demographic data

During the screening visit, demographic information such as the subject's date of birth, age, and gender will be collected.

9.1.3. Medical History Investigation.

The subject's medical history will be thoroughly investigated and recorded through interviews and review of past medical records. Medical history and medication history will include treatment and medication use for weight loss, obesity-related medical history, and medication use, and will be evaluated according to the inclusion criteria (section 6.2) and exclusion criteria (section 6.3).

In the medical history section, information on any known drug allergies will be collected during the screening visit, including the onset date (year and month), duration, and the investigator’s opinion. Any clinically significant medical conditions or abnormal findings prior to the administration of the investigational medicinal product will be considered part of the subject's medical history. Congenital and chronic diseases will be reviewed within the last 5 years, and hypersensitivity to the investigational medicinal product components will be investigated regardless of the time frame.

9.1.4. Confirm Eligibility

The principal investigator or a designated sub-investigator will review all inclusion and exclusion criteria to verify whether the subject is eligible for the clinical trial. Detailed information on the inclusion/exclusion criteria can be

found in sections 6.2 Inclusion Criteria and 6.3 Exclusion Criteria.

9.1.5. Randomization

Subjects who meet the inclusion/exclusion criteria will be randomized into either the treatment group or the placebo group for each cohort, with a ratio of 4:1 (Part A), 3:1 (Part B), or 2:1 (Part C). The randomization table will be generated before the clinical trial using the randomization program in the SAS system, which applies permutations of randomly generated numbers (random numbers) sequentially starting from subject number 1, using SAS® version 9.4 (or higher).

9.1.6. Assignment

Subject numbers will be assigned to subjects who pass the screening and are scheduled for hospitalization within 24 hours from the first scheduled hospitalization. The subject numbers will be assigned in the order in which they pass the screening. The subject number format will consist of the cohort designation (e.g., A1–A5, B1, B2, B3, S, C1, C2), the subject number (e.g., 01, 02, 03...), and an indication of whether the subject is a replacement (0 for original subjects, and a number added to the replacement subject's number corresponding to the subject they replaced).

For example, in cohort A1, the subject numbers will follow the format A1-XXX (e.g., A1-010, A1-020, A1-030…).

A subject can only be assigned one subject number, and no two subjects can share the same subject number. The subject number assigned to each subject will be used as the subject identification code.

If a subject is replaced before receiving the investigational medicinal product, the replacement subject's number will be indicated by adding a digit to the number of the subject they replaced. For example, if subject A1-010 is replaced, the replacement subject will be assigned A1-011, and if this replacement subject is further replaced, the new subject will be assigned A1-012, and so on.

9.1.7. Administration of the investigational medicinal product(IMP)

[redacted]

For Parts B [redacted], the IMP will be administered once a week (V1/D1, V3/D8, V5/D15, V7/D22, V9/D29), with hospitalization on the day before each dose (i.e., on V1/D0). The IMP will be administered on the first day of hospitalization (V1/D1).

For Cohort S (optional cohort), the IMP will be administered every two weeks (V1/D1, V3/D15, V5/D29, V7/D43, V9/D57), and the subjects will be hospitalized and administered the IMP in the same manner as other cohorts in Part B.

The dosage for each cohort will be based on the administration plan in section 2.2 (Clinical Trial Activity Plan) and section 8.5.1 (Dose Escalation Stage).

Subjects must fast for at least 8 hours before receiving the IMP and the administration will be via subcutaneous injection into the abdomen. The dosing times for each subject will be set with appropriate intervals around 9:00 AM on the first day of hospitalization, which will serve as the reference dosing time for the duration of the trial. The IMP will be administered within ±5 minutes of the reference time.

For detailed administration procedures, refer to the investigational medicinal product manual.

9.2. Clinical Procedures and Evaluation

9.2.1. Physical examinations

Physical examinations will be conducted at each visit. The examiner will assess the subject's general condition, including the head, neck, heart, lungs, abdomen, liver, skin, and extremities, to check for any signs of health issues or adverse events. Any abnormal findings in the physical examination that meet the criteria for adverse events must be reported. Additionally, the following exams will be conducted at the designated times during the IMP administration visits:

[redacted]

[Part B] [redacted]

- For the 1st to 4th administrations of the investigational product (V1, V3, V5, V7): Pre-dose (within 1 hour before administration) and at 4 hours ± 1 hour and 6 hours ± 1 hour post-dose (V1/D1, V3/D8*, V5/D15*, V7/D22*).
- For the final administration of the investigational product (V9): Pre-dose (within 1 hour before administration) and at 4 hours ± 1 hour, 8 hours ± 1 hour, and 12 hours ± 1 hour post-dose (V9/D29*), as well as at 24 hours ± 1 hour and 36 hours ± 1 hour post-dose (V9/D30*), and before discharge (V9/D31*).

* For Cohort S (optional), refer to the corresponding days in Section 2.2 Clinical Trial Activities Plan.

9.2.2. Body measurements

Body measurements will be conducted in accordance with the schedule in section 2.2 for assessing weight change and waist-to-hip ratio. Waist and hip circumference will be measured according to Appendix 2. Height (in cm) will be measured at screening, and weight (in kg) will be measured according to the schedule. Height (cm) and weight (kg) will be recorded to the nearest decimal place.

9.2.3. Vital signs

Vital signs include pulse (beats/min), body temperature (℃), sitting systolic and diastolic blood pressure (mmHg), and respiratory rate at each visit. Vital signs are measured before other planned tests, if possible, and after resting in a sitting position for at least 5 minutes. At the time of the investigational medicinal product administration visit, additional procedures are performed according to the schedule below.

[redacted]

[Part B] [redacted]

- For the first administration of the investigational product (V1): Pre-dose (within 1 hour before administration), and at 30 minutes ± 15 minutes, 4 hours ± 1 hour, and 6 hours ± 1 hour post-dose (V1/D1).

- For the 2nd to 4th administrations of the investigational product (V3, V5, V7): Pre-dose (within 1 hour before administration), and at 4 hours ± 1 hour and 6 hours ± 1 hour post-dose (V3/D8*, V5/D15*, V7/D22*).
- For the final administration of the investigational product (V9): Pre-dose (within 1 hour before administration), and at 4 hours ± 1 hour, 8 hours ± 1 hour, and 12 hours ± 1 hour post-dose (V9/D29*), as well as at 24 hours ± 1 hour and 36 hours ± 1 hour post-dose (V9/D30*), and before discharge (V9/D31*).

* For Cohort S (optional), refer to the corresponding days in Section 2.2 Clinical Trial Activities Plan.

9.2.4. Concomitant medications

The investigator or a qualified representative investigates the drug name (ingredient name), purpose of administration, daily administration dose, administration route, and administration period regarding the concomitant drugs being administered from the time of screening to each visit to the institution.

For drugs administered to test subjects within 10 days before the first investigational medicinal product administration, record the drug name (ingredient name), purpose of administration, daily dose, administration route, administration period, etc.

9.2.5. Electrocardiography (12-lead ECG)

Electrocardiogram examination is performed according to the schedule in 2.2 Clinical Trial Activity Plan. The test subject must relax and maintain a stable posture for at least 10 minutes before the test is performed. If irregular heart rate or serious changes are observed, electrocardiogram testing may be reexamined at the discretion of the principal investigator (or delegated investigator).

9.2.6. Laboratory tests and fasting blood glucose

Laboratory tests (blood and blood chemistry tests, coagulation tests, urinalysis, serum tests) will be performed according to the schedule in section 2.2 of the Clinical Trial Activity Plan for each visit. If the subject is admitted within 7 days after the screening test (V1/D-1), the results from the screening test can be used as the V1/D-1 test results. If the laboratory test values at the screening visit meet the inclusion/exclusion criteria, but the test results at the dosing visit fall outside the criteria, the subject may still be enrolled if the investigator (or delegated investigator) determines that the values are clinically insignificant. Laboratory tests may be conducted additionally if clinically necessary, and the test items are referred to in Appendix 1.

Additionally, for repeated dosing, a fasting blood glucose test using a glucose meter will be performed within 1 hour before the administration of the investigational medicinal product, and the investigational medicinal product will only be administered after confirming that the fasting blood glucose is 70 mg/dL or higher.

9.2.7. Pregnancy tests

Pregnancy tests using urine hCG will be conducted only for women of childbearing potential, according to the schedule in section 2.2 of the Clinical Trial Activity Plan. However, at the screening visit, a serum hCG test will be used. Pregnancy tests will not be performed for women who are surgically sterile (e.g., hysterectomy or bilateral tubal ligation) or for postmenopausal women (defined as non-therapy induced amenorrhea for at least 12 months).

A woman of childbearing potential is defined as a woman who has not experienced menopause and has not undergone surgical sterilization. A woman is considered infertile if she meets at least one of the following criteria:

1) Natural amenorrhea for 12 months

2) A serum FSH level exceeding 40 mIU/mL with 6 months of natural amenorrhea,

3) A history of surgical sterilization (e.g., bilateral oophorectomy, tubal ligation).

9.2.8. Blood sampling for PK

The blood sampling schedule for pharmacokinetic (PK) parameter evaluation in this clinical trial for [redacted], [Part B], [redacted] is as follows:

[redacted]

[Part B] [redacted]

- For the 1st to 4th administrations of the investigational product (V1, V3, V5, V7): Pre-dose (within 0.5 hours before administration), and at 4 hours ± 15 minutes and 6 hours ± 15 minutes post-dose (V1/D1, V3/D8*, V5/D15*, V7/D22*).

- For the final administration of the investigational product (V9 to end-of-study visit): Pre-dose (within 0.5 hours before administration), and at 4 hours ± 15 minutes, 6 hours ± 15 minutes, and 12 hours ± 30 minutes post-dose (V9/D29*), as well as at 24 hours ± 1 hour and 36 hours ± 1 hour post-dose (V9/D30*), 48 hours ± 2 hours (V9/D31*), 72 hours ± 2 hours (V10/D32*), 120 hours ± 2 hours (V11/D34*), 168 hours ± 2 hours (V12/D36*), 336 hours ± 2 hours (V13/D43*), and the end-of-study visit (D57*).

*For Cohort S (optional), refer to the corresponding days in Section 2.2 Clinical Trial Activities Plan.

Blood collection methods for pharmacokinetic (PK) evaluation, as well as precautions during blood collection, storage of blood samples for analysis, and sample transportation, will be conducted according to the pharmacokinetic evaluation manual.

9.2.9. Blood sampling for immunogenicity (ADA)

The blood sampling for immunogenicity evaluation in [redacted]. In [Part B] [redacted], blood samples will be collected before the administration of the investigational medicinal product (V1/D-1), on V9/D29 (for Cohort S, this will be V9/D57), and at the end-of-study visit. Guidelines for the collection, storage, and shipping of the blood samples will be provided in a separate manual.

9.2.10. Oral glucose tolerance test (OGTT)

The OGTT will be performed according to the 2021 Diabetes Treatment Guidelines of the Korean Diabetes Association. Prior to the test, the subject must fast for at least 8 hours. After fasting, fasting blood glucose will be measured, and then the subject will drink 75g of glucose dissolved in 250–300mL of water or a commercially available glucose solution within 5 minutes.

[redacted]

For [Part B], blood glucose will be measured at 30 minutes, 60 minutes, 90 minutes, and 120 minutes after drinking the glucose solution. This will be performed on the day of Hospitalization (V1/D-1) and on the second day of Hospitalization (V9/D30*).

[redacted]

*For Cohort S (optional group), this corresponds to V9/D58.

9.2.11. AE assessment

Adverse events (AEs) and serious adverse events (SAEs) will be identified and collected from the first dose of the investigational medicinal product until the end of the clinical trial. At each visit, any new or ongoing AEs or SAEs that occurred since the previous visit will be identified and recorded. Any clinically significant medical condition or abnormal findings prior to the administration of the investigational medicinal product will be collected as medical history.

[redacted]

The evaluation method and detailed information on adverse events are described in section 11, which covers safety evaluation methods, evaluation criteria, and reporting procedures.

9.3. Visit Schedule

9.3.1. Screening Visit (SV)

The screening procedures must be completed within 28 days before the first administration of the investigational medicinal product, and the following steps are performed to confirm the eligibility of the subject:

1) Explain the trial process to the subject before initiating any procedures and obtain written informed consent, then assign a screening number.
2) Confirm the inclusion/exclusion criteria.
3) Collect demographic information and investigate past/present medical history.
4) Perform a physical examination.
5) Conduct body measurements. Refer to Appendix 2 for the methods of measuring height and weight.
6) Measure vital signs (pulse, temperature, blood pressure, respiratory rate).
7) Perform an electrocardiogram (12-lead ECG).
8) Conduct laboratory tests (hematology, blood chemistry, coagulation, serology, and urine tests). Perform a pregnancy test for women of childbearing potential.

   However, if clinically significant abnormal results are found, clinical significance must be confirmed. One re-test is allowed during screening, and the re-test results will be used.
9) Review concomitant medications.
10) Measure body mass index (BMI).

11) Perform a hemoglobin A1c (HbA1c) test.

9.3.2. [Part A] PG-102(MG12) Single Dose Administration

[redacted]

[redacted]

9.3.3. [Part B] [redacted] PG-102(MG12) Multiple Dose Administration

9.3.3.1. 1st Hospitalization (V1/D-1~D1)

Subjects will be admitted on Day -1, prior to the first administration of the investigational medicinal product. After completing all procedures 6 hours post-administration on Day 1, subjects will be discharged. Before admission, inclusion/exclusion criteria will be rechecked to confirm eligibility for enrollment, and subject numbers will be assigned. Education on self-monitoring of blood glucose (SMBG) and device distribution can be conducted at any time during the initial hospitalization period.

V1/D-1(Day of Admission)

1) Inclusion/exclusion criteria will be rechecked to confirm eligibility. Subject numbers will be assigned in the order of screening completion within 24 hours prior to the scheduled admission.
2) A physical examination will be performed.
3) Anthropometric measurements will be conducted. Refer to Appendix 2 for methods of measuring body weight and waist circumference.
4) Vital signs (pulse, temperature, blood pressure, respiratory rate) will be measured.
5) Laboratory tests (hematology, blood chemistry, coagulation tests, and urinalysis) will be conducted. A pregnancy test will be performed for women of childbearing potential. If hospitalization occurs within 7 days after screening, the results of the screening tests may be used as V1/D-1 results.
6) An oral glucose tolerance test (OGTT) will be conducted.

   [Part B] Blood glucose levels will be measured at fasting, and at 30, 60, 90, and 120 minutes after consuming glucose.

[redacted]

7) Body fat measurement (DEXA) will be conducted during the V1/D-1 to V1/D1 period before administration.

8) Blood sampling for immunogenicity evaluation (ADA) will be performed in accordance with Section 9.2.9.

[redacted]

10) Concomitant medications since the last visit will be reviewed.

V1/D1 (Administration of Investigational Medicinal Product and Discharge)

1) Conduct a physical examination.
2) Measure vital signs (pulse, temperature, blood pressure, respiratory rate).
3) Perform fasting blood glucose testing.
4) Collect blood samples for exploratory evaluation (inflammatory biomarkers).
5) Collect blood samples for pharmacokinetic evaluation (in accordance with the sampling time points in Section 9.2.8).
6) Administer the investigational medicinal product (IMP) or placebo.
7) Review adverse events (AEs) and concomitant medications.
8) Discharge the subject.

*Fasting blood glucose testing and blood sampling for exploratory evaluation must be conducted prior to the administration of the investigational medicinal product.

9.3.3.2. 2nd~4th Hospitalization (V3, V5, V7)

V3/D7, V5/D14, V7/D21 (Day of Admission, for Cohort S: V3/D14, V5/D28, V7/D42)

1) Con duct a physical examination.
2) Perform anthropometric measurements. Refer to Appendix 2 for methods of measuring body weight and waist circumference.
3) Conduct 12-lead electrocardiogram (ECG) testing (only at V5/D14 [for Cohort S: V5/D28]).
4) Measure vital signs (pulse, temperature, blood pressure, respiratory rate).

5) Perform laboratory tests (hematology, blood chemistry, and urinalysis; only at V5/D14 [for Cohort S: V5/D28]).

6) Conduct glycated hemoglobin (HbA1c) testing (only at V5/D28 for Cohort S).

7) Review adverse events (AEs) and concomitant medications since the last visit.

V3/D8, V5/D15, V7/D22 (Administration of Investigational medicinal product and Discharge, for Cohort S V3/D15, V5/D29, V7/D43)

1) Conduct a physical examination.

2) Measure vital signs (pulse, temperature, blood pressure, respiratory rate).

3) Perform fasting blood glucose testing.

4) Collect blood samples for pharmacokinetic evaluation (in accordance with the sampling time points in Section 9.2.8).

5) Administer the investigational medicinal product (IMP) or placebo.

6) Review adverse events (AEs) and concomitant medications.

7) Discharge the subject

9.3.3.3. 5th Hospitalization (V9/D28~D31, For Cohort S, V9/D56~D59)

Subjects will be admitted on D28 (D56 for Cohort S) prior to the final administration of the investigational medicinal product and discharged on D31 (D59 for Cohort S) after completing all tests 48 hours post-administration.

V9/D28 (Day of Admission, D56 for Cohort S)

1) Conduct a physical examination.

2) Perform anthropometric measurements. Refer to Appendix 2 for methods of measuring body weight and waist circumference.

3) Measure vital signs (pulse, temperature, blood pressure, respiratory rate).

4) Perform laboratory tests (hematology, blood chemistry, coagulation tests, and urinalysis).

5) Conduct glycated hemoglobin (HbA1c) testing.

6) Collect blood samples for exploratory evaluation (inflammatory biomarkers).

7) Review adverse events (AEs) and concomitant medications since the last visit.

V9/D29 (Administration of Investigational Medicinal Product, D57 for Cohort S)

1) Conduct a physical examination.

2) Measure vital signs (pulse, temperature, blood pressure, respiratory rate).

3) Perform fasting blood glucose testing.

4) Collect blood samples for pharmacokinetic evaluation (in accordance with Section 9.2.8 sampling time points).
5) Collect blood samples for immunogenicity evaluation.
6) Administer the investigational medicinal product (IMP) or placebo.
7) Review adverse events (AEs) and concomitant medications.

V9/D30 (D58 for Cohort S)

1) Conduct a physical examination.
2) Measure vital signs (pulse, temperature, blood pressure, respiratory rate).
3) Perform body fat measurement (DEXA) between V9/D30 and V9/D31.
4) Conduct an oral glucose tolerance test (OGTT)

   [Part B]: Measure blood glucose levels at fasting, and 30, 60, 90, and 120 minutes after consuming glucose[redacted]
5) Collect blood samples for pharmacokinetic evaluation (in accordance with Section 9.2.8 sampl ing time points).

[redacted]

7) Review adverse events (AEs) and concomitant medications.

V9/D31 (Discharge, D59 for Cohort S)

1) Conduct a physical examination.
2) Perform a 12-lead electrocardiogram (ECG).
3) Measure vital signs (pulse, temperature, blood pressure, respiratory rate).
4) Perform laboratory tests (hematology, blood chemistry, coagulation tests, and urinalysis).
5) Collect blood samples for pharmacokinetic evaluation (in accordance with Section 9.2.8 sampling time points).
6) Review adverse events (AEs) and concomitant medications.
7) Discharge the subject

9.3.3.4. Telephone Visit (V2, V4, V6, V8)

Adverse events (AEs) and concomitant medications since the last visit will be reviewed via telephone.

9.3.3.5. V10, V11

1) Conduct a physical examination.
2) Measure vital signs (pulse, temperature, blood pressure, respiratory rate).
3) Collect blood samples for pharmacokinetic evaluation (in accordance with Section 9.2.8 sampling time points).
4) Review adverse events (AEs) and concomitant medications since the last visit.

9.3.3.6. V12, V13

1) Conduct a physical examination.
2) Perform anthropometric measurements. Refer to Appendix 2 for methods of measuring body weight and waist circumference.
3) Measure vital signs (pulse, temperature, blood pressure, respiratory rate).
4) Perform laboratory tests (hematology, blood chemistry, and urinalysis).
5) Collect blood samples for pharmacokinetic evaluation (in accordance with Section 9.2.8 sampling time points).
6) Review adverse events (AEs) and concomitant medications since the last visit.

9.3.4. End of Study Visit

The following procedures are performed during the end-of-study visit after the administration of the investigational medicinal product PG-102(MG12).

1) Perform a physical examination.
2) Measure physical parameters. For weight and waist circumference, refer to Appendix 2 for the measurement methods.
3) Measure vital signs (pulse, body temperature, blood pressure, respiratory rate).
4) Perform a 12-lead ECG (electrocardiogram).
5) Conduct laboratory tests (hematology, biochemistry, coagulation tests, and urinalysis). Pregnancy tests are performed for women of childbearing potential.
6) Conduct a HbA1c (glycated hemoglobin) test.
7) Perform exploratory assessments (inflammatory biomarkers) by collecting blood samples (only in Part B [redacted]).
8) Perform pharmacokinetic (PK) evaluations and immunogenicity assessments (ADA) by collecting blood samples.
9) Adverse events and concomitant medications since the last visit will be reviewed.

For subjects who are withdrawn early, the procedures for the end-of-study visit will be determined based on the evaluation items and the investigator's judgment.

9.3.5. Unscheduled visit

Unscheduled visits refer to visits that occur outside of the regular scheduled visits. These visits may be conducted at any time based on the investigator's judgment if an additional visit is necessary for the subject. Unscheduled visits may occur if, during the clinical trial, laboratory tests do not return to baseline (or normal range) or do not show trends toward recovery. Additionally, if an adverse event has not resolved after an unplanned visit or if laboratory tests do not return to baseline (or normal range), or do not show trends toward recovery, an unscheduled visit may also be conducted. During an unscheduled visit, the investigator may perform necessary tests, evaluations, and blood collection as needed.

If for any reason a subject visits on a day other than the scheduled date, the occurrence must be documented in the electronic case report form (eCRF) or other relevant forms. The visit schedule should not be altered due to an unscheduled visit.

9.4. Lifestyle and Dietary Restrictions.

The subjects must fast for 8 hours prior to the administration of the investigational medicinal product, excluding water. However, in the case of Part B [redacted], if the subject's fasting blood glucose is below 70 mg/dL prior to the administration of the investigational medicinal product, they may consume 15 g of sugar.

The subjects are instructed to maintain their usual diet but are advised to avoid excessive low-calorie or high-calorie diets.

9.5. Safety Evaluation

Safety evaluation is conducted through the monitoring of adverse events (both subjective and objective), vital signs, physical examinations, laboratory tests (hematological, biochemical, coagulation, urinalysis), electrocardiogram (ECG) tests, and immunogenicity assessment (ADA).

9.6. Pharmacokinetic Evaluation

The pharmacokinetic evaluation of PG-102(MG12) in blood after the administration of the investigational medicinal product will be conducted in [redacted], [Part B][redacted]. Blood for pharmacokinetic parameter (PK) evaluation will be collected according to section 9.2.8 and the clinical trial activity plan in section 2.2 to assess the concentration of PG-102(MG12) in serum. Detailed procedures for the pharmacokinetic evaluation will be followed according to the pharmacokinetic evaluation manual.

9.7. Pharmacodynamic Evaluation

Pharmacodynamic evaluation will be performed through assessments such as weight change, waist circumference, waist-hip ratio, body fat measurement (DEXA), HbA1c levels, and oral glucose tolerance tests (OGTT). Body fat measurement (DEXA) will be conducted only in [Part B] [redacted]

9.8. Exploratory Evaluation

For exploratory evaluation, blood samples from trial subjects in [Part B] and [Part C] may be collected and stored, during which plasma hsCRP, an inflammatory biomarker, will be evaluated as part of the exploratory assessment. [redacted] Blood and fecal samples will be collected according to the clinical trial activity plan outlined in section 2.2.

10. Completion of the Trial and Withdrawal of Subjects

10.1. Criteria for Completion of Clinical Trial Subjects

In this clinical trial, a subject who completes the final visit is defined as a "completed subject."

A subject who is withdrawn early from the trial will conclude their participation after completing the scheduled procedures for the final visit.

If a subject fails to attend the scheduled visit or refuses to participate, the investigator (or designated sub investigator) must make every effort to contact the subject via available methods, such as written or telephone communication. All attempts and related matters should be documented as evidence.

10.2. Criteria for Early Termination of Clinical Trials

The investigator or blinded safety review committee (SRC) may decide to stop the clinical trial, either partially or entirely, if it is determined that continuing the trial is not appropriate based on the results observed during the trial, or for safety or management reasons. This may occur if new information arises about the investigational medicinal product that negatively impacts the benefit-risk ratio for subjects. The reasons for early termination include, but are not limited to, the following.

- The occurrence of new and significant drug-related adverse events, or if the incidence and severity of expected drug-related adverse events are deemed to be excessively high and unexpected.
- If the sponsor of the investigational medicinal product determines that continuing the clinical trial is not justified from a medical or ethical standpoint.
- If the supply of the investigational medicinal product is discontinued.

If the clinical trial is subject to early termination, the decision to continue the trial will be evaluated by the Safety Review Committee (SRC). Additionally, if requested by government regulatory authorities, the clinical trial may be halted or discontinued.

In the event of early termination or discontinuation of the trial, the investigator must inform the Institutional Review Board (IRB) in accordance with the relevant regulations. Furthermore, the investigator must immediately notify the trial Subjects, ensure appropriate actions and follow-up are taken, and return the investigational medicinal product to the sponsor.

10.3. Criteria for Participant Early Withdrawal from the Trial

The completion status of all Subjects in the clinical trial must be documented, and if drug administration or observation is discontinued, the reason for discontinuation must be recorded. Subjects may discontinue treatment or the trial if any of the following occur:

1) Voluntary withdrawal of consent by the participant (or their legal representative).
2) Participation of a participant who does not meet the inclusion/exclusion criteria.
3) Determination that the administration of concomitant medication, which could influence the study results, is necessary during the trial period.
4) Unacceptable toxicity based on the potential reactions and severity of adverse events observed in individual Subjects related to the investigational medicinal product.

5) The investigator's judgment that the participant's condition has changed, making further participation unsafe or unethical.
6) Confirmation of pregnancy during the investigational medicinal product administration period.
7) Other cases where the investigator deems it necessary to discontinue the trial.
8) Discontinuation of the clinical trial by the sponsor's decision.

11. Methods for Assessing Safety, Evaluation Criteria, and Reporting Procedures, including Adverse Events

11.1.1. Definitions of Safety-Related Terms

1) Adverse Event, AE

Any harmful and unintended sign (e.g., abnormal laboratory findings), symptom, or disease occurring in Subjects who have received the investigational medicinal product, regardless of its causal relationship with the investigational medicinal product.

2) Treatment-emergent adverse event, TEAE

An AE that occurs after the administration of the investigational medicinal product or a pre-existing condition that worsens in intensity or frequency following administration.

3) Adverse Drug Reaction, ADR

A harmful and unintended response to any dose of the investigational medicinal product where a causal relationship with the product cannot be ruled out.

4) Serious AE·ADR

A severe AE or ADR that meets one or more of the following criteria.

- Death.
- Is life-threatening.
- Requires hospitalization or prolongation of existing hospitalization.
- Results in permanent or significant disability or functional impairment.
- Causes congenital anomalies or birth defects.
- Leads to other medically significant conditions such as dependency, abuse, or critical blood disorders.

The following do not qualify as "hospitalization" for SAE reporting purposes:

- Planned procedures (e.g., surgery, tests) arranged before trial enrollment.
- Hospitalization or extended stays for monitoring resolved or improving conditions.
- Hospitalizations for diagnostics or educational purposes.
- Non-medical reasons (e.g., temporary absence of family support).
- Transfers to hospice, nursing homes, or rehabilitation facilities.
- Emergency room visits lasting less than 24 hours (hospitalizations exceeding 24 hours are considered SAEs).
- Hospitalizations planned before the consent process.

Other medically important situations are considered serious adverse events (SAEs) if, based on the investigator's medical judgment, they pose a significant risk to the subject or require intervention to prevent the listed symptoms, even if they do not immediately threaten life, result in death, or require hospitalization.

Even if the situations mentioned above do not apply, any circumstance deemed to have a significant impact on the safety and health of the trial subject from a medical standpoint should be evaluated by the responsible physician (the investigator or a designee sub- investigator) and relevant experts. They will determine, based on their medical judgment, whether the event should be classified as a serious adverse event and take appropriate action accordingly.

11.1.2. Evaluation Criteria for Adverse Events

11.1.2.1. Severity

The severity of adverse events is determined using the adverse event severity grading scale from the latest NCI CTCAE v5.0. For severity assessment of adverse events not included in the NCI CTCAE, refer to the table below.

[Table 6] Severity assessment scale for adverse events not included in the NCI CTCAE

Grade	Severity	Descriptions
1	Mild	Asymptomatic or mild symptoms; clinical or diagnostic observations only; intervention not indicated.
2	Moderate	Minimal, local or noninvasive intervention indicated; limiting age-appropriate instrumental IADLs[a].
3	Severe	Severe or medically significant but not immediately life-threatening; hospitalization or prolongation of hospitalization indicated; limiting self-care ADL[b, c].
4	Life threatening	Life-threatening consequences or urgent intervention indicated[d].
5	Death	Death related to AE[d].

[a] Instrumental activities of daily living (IADLs) refer to preparing meals, shopping for groceries or clothes, using the telephone, managing money, etc.

[b] Self care ADL refer to bathing, dressing and undressing, feeding self, using the toilet, taking medications, and not bedridden.

[c] When an adverse event is considered a "medically significant event", it should be reported as a serious adverse event.

[d] Grade 4 and 5 adverse events should be reported as serious adverse events according to the definition of a serious adverse event.

11.1.2.2. Evaluation Criteria for Causality with the Investigational Medicinal Products

In the event of an adverse event, the investigator evaluates the relationship with the investigational medicinal product as follows: 'Clearly related,' 'Likely related,' or 'Suspected to be related.' If the evaluation is 'related' or 'likely related,' it is considered to be related

1) Definitely related

- If there is evidence that the investigational medicinal product has been administered.
- If the temporal sequence between the administration of the investigational medicinal product and the occurrence of an adverse event is reasonable.
- If the adverse event can be most plausibly explained by the administration of the investigational medicinal product rather than any other reason.
- If the adverse event disappears upon discontinuation of the investigational medicinal product (if applicable).
- If re-administration (if possible) results in a positive outcome.
- If the adverse event shows a pattern consistent with known information about the investigational

medicinal product or drugs of the same class.

2) Probably related

- If there is evidence that the investigational medicinal product was administered,
- If the temporal sequence between the administration of the investigational medicinal product and the occurrence of the adverse event is plausible,
- If the adverse event is more convincingly explained by the administration of the investigational medicinal product than by other causes,
- If the adverse event resolves after discontinuation of the investigational medicinal product (if applicable).

3) Possibly related

- If there is evidence that the investigational medicinal product was administered,
- If the adverse event is judged to be attributable to the drug, on par with other possible causes,
- If the adverse event disappears after discontinuation of the investigational medicinal product (if performed)

4) Unlikely, probably not related

- If there is evidence that the investigational medicinal product was administered,
- If there is a more likely cause for the adverse event,
- If the result of discontinuing the investigational medicinal product (if performed) is negative or ambiguous,
- If the result of re-administration (only if applicable) is negative or ambiguous.

5) *Unknown, unassessable

- If the information is insufficient or contradictory and cannot be assessed, and it cannot be supplemented or confirmed,

  *If the relationship between the investigational medicinal product and the adverse event is unclear at the time of the initial adverse event occurrence, and additional tests or follow-up observations regarding

6) Not related, None

- If the trial participant did not receive the investigational medicinal product, or
- If the temporal sequence between the drug administration and the onset of the adverse event is not plausible, or
- If there is another clearly identifiable cause for the adverse event.

11.1.2.3. Treatment and Outcome

The principal investigator and a designated sub-investigator must ensure the utmost safety of the study subjects during the clinical trial. In the event of a serious adverse event (SAE) or adverse drug reaction (ADR), prompt and appropriate measures must be taken to minimize the impact of the adverse event. The principal investigator, in consultation with the sponsor, may decide to discontinue the trial.

All adverse events observed during the clinical trial, regardless of their relation to the investigational medicinal product, must be thoroughly documented in the case report form (CRF), including details of symptoms and signs, onset date/end date, severity, treatment and outcomes, causality with the investigational medicinal product, and seriousness. Furthermore, these adverse events must be monitored until they return to the pre-administration state or baseline, or until the principal investigator or a designated sub-investigator deem the adverse event resolved or further observation unnecessary.

1) Action Taken with Investigational medicinal product

① Dose maintained

② Temporarily discontinued

③ Permanently discontinued

④ Unknown

⑤ Not applicable

2) Action Taken Outside of Investigational medicinal product

① No treatment given

② Administered therapeutic medication

③ Non-pharmacological intervention

④ Both pharmacological and non-pharmacological interventions

3) Outcome

① Recovered/Resolved

② Recovering/Resolving

③ Not recovered/Not resolved

④ Recovered with sequelae/Resolved with sequelae

⑤ Death

⑥ Unknown

11.1.3. Reporting Methods

11.1.3.1. Reporting of Adverse Events

The investigator is responsible for educating the clinical trial subjects (or their representatives) about all potential adverse events that may occur after the administration of the investigational medicinal product. The investigator ensures that the subjects are instructed to report any symptoms that appear after the administration of the drug.

The investigator records all symptoms resulting from the use of the investigational medicinal product, including local/systemic reactions or laboratory abnormalities, in the case report form. These records should include the type of symptoms, the onset and resolution dates, severity, treatment and outcome, as well as the relationship to the investigational medicinal product. Additionally, drug-related adverse events should be followed up until they are resolved (e.g., when the adverse event disappears or follow-up is no longer possible).

11.1.3.2. Expedited Reporting

The investigator must report all serious adverse events (SAEs) that occur during the clinical trial to the sponsor immediately or within 24 hours of becoming aware of the event, regardless of their relationship to the investigational medicinal product. The initial report should, as much as possible, include all details required in the SAE report form and must also be documented in the adverse event section of the case report form (CRF). Additionally, safety information must be reported in compliance with the reporting criteria and timelines established by the clinical trial site's Institutional Review Board (IRB).

The sponsor, upon receiving the initial report, must review the information, contact the investigator for additional details if necessary, and assess the causality between the SAE and the investigational medicinal product.

For serious and unexpected adverse drug reactions (SUSARs), the following reporting deadlines apply to the Ministry of Food and Drug Safety (MFDS) and, if required, the IRB:

- For events resulting in death or considered life-threatening: The initial report must be submitted within 7 days of becoming aware of the event.

  If all required information (e.g., name of the adverse reaction, final observation results, summary of the adverse reaction) according to Adverse Drug Reaction Report Form No. 77 is not available at the time of the initial report, the sponsor must submit a follow-up report with detailed information within 15 days of first becoming aware of the event.

- For other serious and unexpected adverse drug reactions: The report must be submitted within 15 days of becoming aware of the event.

11.1.4. Other Reportable Information

11.1.4.1. Pregnancy

If a female subject or the spouse (or partner) of a male subject becomes pregnant during the clinical trial, the investigator must complete an initial pregnancy report and submit it to the sponsor within 24 hours of becoming aware of the pregnancy. The investigator must track and document the progress and outcome of all pregnancies, even if the subject withdraws consent or discontinues participation in the clinical trial. Additionally, upon becoming aware of the delivery or the outcome of any pregnancy, the investigator must complete a follow-up pregnancy report and submit it to the sponsor within 24 hours.

If a subject is confirmed to be pregnant during the clinical trial, this is not considered an adverse event; however, the subject must withdraw from the trial. The progress of the pregnant subject and fetus must then be tracked and reported accordingly.

11.1.5. Follow-up of Adverse Events

Adverse events collected during the study will be monitored until the scheduled dosing and all visit procedures are completed. However, if new adverse events occur at the final visit or ongoing adverse drug reactions remain unresolved, follow-up observation will continue until the adverse drug reaction is resolved or the investigator determines that further follow-up is unnecessary. The investigator must make reasonable efforts to gather information on unresolved adverse drug reactions by the end of the clinical trial or after the subject's discontinuation from the trial and ensure that all related matters are appropriately documented.

12. Data Analysis and Statistical Considerations

12.1. Analysis Groups

1) Safety Evaluation Group: Refers to the group of trial Subjects who were randomly assigned and received at least one dose of the investigational medicinal product in this clinical trial.

2) Pharmacokinetics Evaluation Group: Refers to the group of Subjects who received the investigational medicinal product, completed the planned pharmacokinetic blood sampling, and have a measurable concentration of PG-102(MG12).

3) Pharmacodynamics Evaluation Group: Refers to the group of Subjects who received the investigational medicinal product and have measurable pharmacodynamics evaluation results after receiving at least one dose of the study drug.
4) Immunogenicity Evaluation Group: Refers to all Subjects who received the investigational medicinal product and have evaluable immunogenicity results for the study drug in any form. However, Subjects who did not receive the investigational medicinal product at least once will be excluded from the analysis group.

12.2. Statistical Analysis Methods

This clinical trial is designed to evaluate the safety, tolerability, and efficacy of the investigational medicinal product, and hypothesis testing for statistical analysis will not be performed. All statistical analyses will be summarized using descriptive statistics with R version 4.0 or higher. For descriptive statistical summaries, continuous variables will be presented with the number of subjects, mean, standard deviation, median, minimum, and maximum, while categorical variables will be presented with frequencies and percentages.

12.2.1. Demographic and Clinical History Data

Demographic information of the subjects (such as age, gender, etc.) and baseline characteristics prior to treatment will be presented for the safety analysis population. For continuous variables, descriptive statistics (number of subjects, mean, standard deviation, median, minimum, maximum) will be provided, while for categorical variables, frequencies and percentages will be presented.

All collected medical history information will be standardized using MedDRA Version 22.0 (or a later version) to System Organ Class (SOC) and Preferred Term (PT). For the safety analysis population, the frequency, percentage, and number of occurrences will be presented for each SOC and PT.

12.2.2. Analysis of Safety Evaluation Parameters

The analysis of safety will be conducted for the safety evaluation population.

1) Adverse Event

For treatment-emergent adverse events (TEAEs), adverse drug reactions (ADRs), serious adverse events (SAEs), and serious adverse drug reactions (SADRs) that occur after the administration of the investigational medicinal product, the number of subjects experiencing the events, the incidence rate (%), and the number of occurrences will be presented.

Adverse events will be summarized by distinguishing between local adverse events related to the administration site of the investigational medicinal product and systemic adverse events unrelated to the administration site. All reported adverse events will be standardized using MedDRA Version 22.0

(or a later version) by SOC and PT. The frequency, percentage, and number of occurrences will be presented for each SOC and PT.

2) Vital Signs, Laboratory Tests, Electrocardiogram (ECG) Tests, and Physical Examinations

For vital signs, laboratory test results, and electrocardiogram (ECG) results, continuous variables are summarized using descriptive statistics (number of subjects, mean, standard deviation, median, minimum, and maximum) for the change from baseline at each time point.

If necessary, shift tables (or plots) can be presented by treatment group and dose group for items that are expected to show clinically significant differences before and after administration, and appropriate statistical tests can be performed.

3) Immunogenicity

Anti-drug antibodies (ADA) are measured using validated methods, and descriptive statistics are presented for the change from baseline at each time point.

12.2.3. Analysis of Pharmacodynamic (PD) Evaluation Parameters

Continuous data is summarized using descriptive statistics (such as number of observed subjects, mean, standard deviation, median, minimum, and maximum), while categorical data is summarized by the number of subjects and percentages for each category.

12.2.4. Analysis of Pharmacokinetic (PK) Evaluation Parameters

Pharmacokinetic evaluation is performed using WinNonlin or the R program to calculate pharmacokinetic parameters using the noncompartmental method. The calculated pharmacokinetic parameters are summarized with descriptive statistics (such as number of subjects, mean, standard deviation, median, minimum, and maximum) for each subject and treatment group.

12.2.5. Interim Analysis

A interim analysis may be conducted to assess the safety, pharmacodynamics, and pharmacokinetics for each part or cohort. The interim analysis can be performed once the data lock is completed after the termination of the part or cohort intended for analysis.

Only designated personnel are allowed to perform the interim

13. Data Management

13.1. Record Management and Access

13.1.1. Source Documents

Source documents are defined as the results of data collection activities and observations in the clinical trial. Source documents include medical records, electronic data, and records obtained from devices, and may include other records. All source documents in this clinical trial shall be recorded and maintained by the investigator at the clinical trial site, and only authorized personnel shall have access to and review the source documents.

13.1.2. Data Collection

All EDC systems used in this clinical trial may only be accessed by authorized personnel, and all tracking of entering, modifying, saving, and deleting electronic case report forms (eCRFs) through the EDC system will be recorded. The investigator shall certify, via electronic signature, that the data entered into the eCRF is accurate, complete, legible, and timely. Electronic case report forms created through the EDC system will be transferred to an electronic storage medium at the end of the trial and delivered to the clinical trial site, and will be stored under the same standards as other source documents.

13.1.3. Record Protection and Retention

In accordance with applicable regulations governing the retention of clinical trial records, the investigator shall retain, under appropriate storage conditions, all records related to the conduct of the clinical trial, including the clinical trial protocol, records of the manufacture and control of the investigational product, and other related documents (including electronic records), for a period of 3 years from the date of approval. The sponsor may request an extension of the retention period if deemed necessary.

These records shall be subject to inspection by the relevant regulatory authorities. The investigator shall take reasonable precautions to prevent loss or premature damage to these records.

13.1.4. Data Safety Monitoring Plan

The investigator shall implement measures to protect the confidentiality of subject data by ensuring that all documents related to this clinical trial are not disclosed to unauthorized parties. To guarantee the integrity of the trial data, the investigator shall periodically verify the storage location of source documents and ensure that there has been no unauthorized access. Furthermore, the investigator shall review whether the clinical trial is being conducted in accordance with the clinical trial protocol (e.g., appropriateness of subject recruitment, safety and efficacy assessments, and timely reporting of adverse events) to protect the safety of subjects and ensure data completeness by creating case report forms based on source documents.

The monitor shall report safety data from this monitoring process to the investigator for medical judgment. The investigator shall make a final decision based on recommendations from the safety monitoring committee, which shall include:

1) Recommendation to continue or terminate the clinical trial.
2) Recommendation on improvements to subject recruitment, selection, retention, and management, adherence to the clinical trial protocol, and data management and quality control procedures to ensure the integrity of the clinical trial.
3) Evaluation of information regarding risks that outweigh the benefits of the investigational product,

adverse events, or lack of efficacy, and recommendation on how to mitigate risks such as adverse events.

4) Review of monitoring reports on protocol violations and early withdrawals, and assessment of data completeness.

14. Ethical Considerations and Administrative Procedures

14.1.1. Good Clinical Practice and the Declaration of Helsinki

This clinical trial will be conducted in accordance with the Good Clinical Practice (GCP) guidelines specified in [attached 4] of the Pharmaceutical Affairs Act Enforcement Regulations and the International Council for Harmonisation of Technical Requirements for Pharmaceuticals for Human Use (ICH) Guideline E6 (R2). All relevant regulations will be adhered to, ensuring that the trial is conducted ethically and scientifically.

Furthermore, this clinical trial will be conducted in accordance with the Declaration of Helsinki, respecting the dignity and rights of human subjects and ensuring that no harm comes to the subjects

14.1.2. Institutional Review Board (IRB)

Prior to initiating the clinical trial, the investigator must obtain written approval from the Institutional Review Board (IRB) for the clinical trial protocol, subject information and consent forms, and materials and procedures related to subject recruitment. The IRB of each clinical trial institution shall review the ethical, scientific, and medical soundness of the clinical trial and provide a written decision to the investigator and sponsor prior to the start of the trial.

The investigator shall report to the IRB the progress of the trial, serious adverse events, life-threatening problems, or deaths, and shall notify the IRB upon completion of the trial.

The investigator shall obtain written informed consent from the subject (or the subject's legal representative) prior to conducting the trial, after fully explaining the nature and procedures of the trial, the potential benefits and risks of the investigational product. Written informed consent must be obtained prior to any trial procedures. The investigator shall provide the subject (or the subject's legal representative) with a copy of the signed consent form and one copy of the information sheet, and the original shall be kept in the investigator's file.

Under no circumstances shall the investigator or sub-investigator coerce or unduly influence the subject to participate in the trial. Informed consent forms and any information (verbal or written) related to the clinical trial shall not contain any content that restricts or implies the restriction of the rights of the subject or the subject's legal representative, or that waives or implies the waiver of the liability of the investigator, the clinical trial institution, the sponsor, or the sponsor's representative.

Furthermore, easy-to-understand language should be used when obtaining consent from the subject.

Before obtaining the subject's consent, the investigator or a physician delegated by the investigator shall provide the subject or the subject's legal representative with sufficient time and opportunity to ask questions about the details of the clinical trial and to decide whether to participate in the trial, and shall answer all questions related to the clinical trial truthfully.

If the subject or the subject's legal representative is unable to read the consent form, subject information sheet, or other written information, a witness must be present during the entire consent process. In this case, the investigator or a delegate of the investigator shall read and explain the consent form, subject information sheet, and other written information to the subject or the subject's legal representative. The subject or the subject's legal

representative shall verbally agree to participate in the clinical trial and, if possible, sign and date the consent form. The witness shall also sign and date the consent form. Prior to signing the consent form, the witness shall confirm the following:

1) Whether the consent form, subject information sheet, and other written information have been accurately explained to the subject or the subject's legal representative.
2) Whether the subject or the subject's legal representative has understood the information.
3) Whether the consent process has been conducted voluntarily by the subject or the subject's legal representative.

By signing the consent form, the subject agrees to participate in the clinical trial and to the collection and use of their personal information related to the clinical trial. Personal information collected in connection with the clinical trial includes personally identifiable information, demographic information, medical records (past medical history, treatment history, etc.), and test results performed in connection with the clinical trial. All collected data will be handled in accordance with applicable laws, regulations, and guidelines regarding the protection of personal information.

The Institutional Review Board (IRB) will evaluate and approve this clinical trial protocol in accordance with the Korean Good Clinical Practice (KGCP) guidelines and will conduct periodic reviews to ensure that the clinical trial is being conducted in accordance with the protocol. All study personnel, including the investigator, shall thoroughly review and understand the clinical trial protocol. The investigator shall take adequate measures to address unexpected adverse events, report as necessary, and provide adequate education to the subjects. The conduct of the clinical trial shall be in compliance with the KGCP guidelines.

14.2. Subject Confidentiality

All subject names will be kept confidential and records will be kept and evaluated using the identification numbers assigned at the start of the trial. Subjects will be informed that all clinical trial data will be stored in a computer and treated as strictly confidential. Signed subject consent forms will be retained by the investigator. The investigator will maintain a list of subject identification numbers and names for future reference.

Subject consent forms and subject lists will be retained by the clinical trial site for 3 years from the date of approval. Subsequent retention and management will be determined in consultation with the sponsor. To prevent potential errors, the investigator will contact the sponsor prior to discarding the retained records. Additionally, subject information obtained during the trial will not be disclosed to any third party, except to government agencies, sponsors, monitors, auditors, and the Institutional Review Board (IRB).

14.3. Subject Compensation Agreement

In the event of any adverse event caused by the investigational product, the sponsor shall compensate the subject in accordance with the subject compensation agreement.

15. Protocol Amendment

Any changes to an approved clinical trial protocol must be approved by the Institutional Review Board (IRB) and, if necessary, reported to or approved by the Ministry of Food and Drug Safety.

16. Protocol Violations and Deviations

This clinical trial must be conducted in accordance with the protocol approved by the Ministry of Food and Drug Safety and/or the Institutional Review Board (IRB).

Violations and deviations refer to any non-compliance with the clinical trial protocol, KGCP guidelines, and clinical trial manuals. Non-compliance can occur with anyone involved in the clinical trial, including subjects, investigators, and other study personnel. To prevent protocol deviations and violations, clinical trial staff and study investigators shall have a thorough understanding of the clinical trial protocol, receive adequate training, establish procedures to address unexpected adverse events, and the clinical trial site shall develop measures to correct any deviations or violations and take immediate corrective actions when they occur. All aspects of the clinical trial shall be conducted in accordance with the clinical trial protocol and relevant regulations. Any deviations or violations shall be documented and reported, including the nature of the deviation or violation, the reason, and the corrective actions taken.

17. Quality control and Quality Assurance

1) Monitoring of the Clinical Trial Institution

Monitoring will be conducted to ensure the protection of subjects' rights and welfare, to verify that reported clinical trial data are accurate, complete, and verifiable by comparison with source documents, and to confirm that the clinical trial is being conducted in accordance with the approved protocol and relevant KGCP regulations.

Monitoring of the clinical trial will be conducted through regular visits and communications by the monitor. The timing of these visits will be mutually agreed upon by the investigator and the monitor.

Monitors will primarily verify source documents, records of investigational product management, and the condition of document storage, and will review the overall conduct of the clinical trial. The monitor shall discuss any identified issues with the investigator, and the investigator shall cooperate in this process.

2) Audit

In addition to routine monitoring, audits may be conducted to ensure the reliability of the clinical trial. Audits will include verification that the clinical trial is being conducted in accordance with the clinical trial protocol, standard operating procedures, and KGCP, and a review of all source data, drug records, and case report forms. Authorized representatives may request access to source documents and other original records for the purpose of auditing the site, and the investigator shall permit such access and cooperate in this process.

3) Inspections

The Ministry of Food and Drug Safety may conduct inspections during or after the clinical trial. If an inspection is scheduled, the investigator shall immediately notify the sponsor. The Ministry of Food and Drug Safety may request access to source documents and other original records for the purpose of inspecting the site, and the investigator shall permit such access and cooperate in this process.

18. Investigator Information

Information on the investigator and the clinical trial site will be specified separately in Attachment.

19. Appendix

19.1. Appendix 1 Laboratory Test

Hematology test	Hematocrit
	Hemoglobin
	Erythrocyte count (RBC)
	Mean cell volume
	Mean cell hemoglobin
	Mean cell hemoglobin concentration
	Leukocytes (WBC)
	Platelets
	Differential WBC Absolute counts of:
	Neutrophils
	Lymphocytes
	Monocytes
	Eosinophils
	Basophils
Blood chemistry test (Fasting)	Sodium
	Potassium
	Chloride
	Serum creatinine
	Calcium
	Phosphorus
	Glucose
	Insulin
	Blood urea nitrogen
	Uric acid
	Total protein
	Albumin
	Total bilirubin
	Alkaline phosphatase
	Alanine aminotransferase (ALT)
	Aspartate aminotransferase (AST)
	Lipase
	Amylase
	Total cholesterol
	Triglyceride
	eGFR
	Creatinine clearance (only screening)
Blood coagulation test	Prothrombin time (PT)
	Partial thromboplastin time (PTT)
	International normalized ratio (INR)
Urinalysis	Specific gravity
	pH
	Protein
	Glucose
	Ketones
	Bilirubin
	Urobilinogen
	Occult blood

	Leukocytes
	Microscopy
Lipid Panel	HDL-C
	LDL-C (calculated)
	Total cholesterol
	Triglyceride
Serology	Hepatitis B surface antigen
	Hepatitis C antibody
	HIV antibody and/or HIV antigen
	Syphilis reagin test
Biomarkers	plasma hsCRP
Others	Pregnancy test (urine, serum)
	Hemoglobin A1c
	OGTT serum glucose
	Microbiota Test

19.2. Appendix 2 Measurement of weight, height, and waist circumference

1) Height Measurement

Step 1: Have the subject take off his/her shoes and hat and step onto the stadiometer.

Step 2: Have the subject stand on the stadiometer or lean against the wall, with his/her feet together, knees straight, and heels against the stadiometer backboard or wall.

Step 3: Have the subject look straight ahead without raising or lowering his/her head.

Step 4: Have the subject inhale and stand upright, and measure height. Record the measurement in centimeters (cm) to the first decimal place.

2) Weight Measurement

Weight is measured consistently using a calibrated electronic scale that measures in kilograms (kg) to the first decimal place. The subject should urinate before the measurement and use the same scale if possible. In addition, weight should be measured in a fasting state. If the subject is not fasting, the measurement should be taken again during the visit within the allowable range of visits.

Step 1: Have the subject remove shoes, outer clothing (coat, jacket, etc.), and hat. Step 2: The subject steps onto a scale placed on a flat surface, placing one foot on each side of the scale.

Step 3: Have the subject stand still with their arms at their sides, and record their weight in kilograms (kg) to the first decimal place.

3) BMI Calculation

It is calculated based on height and weight, and is rounded down to the third decimal place and recorded to the second decimal place.

$$\text{BMI} = \frac{\text{Weight (kg)}}{\text{Height (m)}^2}$$

Height (cm) and weight (kg) are recorded to the first decimal place

4) Waist circumference measurement

Waist circumference is measured at the midpoint between the lower edge of the last palpable rib and the upper iliac crest. Measure using an inelastic tape measure, lying flat on the skin without compressing soft tissue. Waist circumference is measured twice and rounded to the nearest 0.5 cm. The results of the two measurements are recorded on the electronic case record sheet. If the difference between the two measurements exceeds 1 cm, discard this set of measurements and measure twice more and record them.

Step 1: Have the subject wear thin clothing, stand with both feet together, both arms down at the side, and weight evenly distributed.

Step 2: Have the subject maintain a relaxed state, and then measure the waist circumference.

5) Hip circumference measurement

Hip circumference is measured as the circumference of the greater trochanter of the femur or the widest part of the body. Using an inflexible tape measure, place it flat on the skin without compressing the soft tissue, measure twice, round to the nearest 0.5 cm, and record the results of the two measurements in the electronic case record. If the difference between the two measurements exceeds 1 cm, discard this set of measurements and measure

twice more and record them.

6) Waist-to-hip ratio (WHR)

The waist-to-hip ratio is calculated by taking the average of the two measurements of waist and hip circumferences

$$\mathrm{WHR} = \frac{\mathrm{Waist\ circumference\ (cm)}}{\mathrm{Hip\ circumference\ (cm)}}$$

19.3. Appendix 3 Self-Blood Glucose Monitoring

For self-blood glucose monitoring, the investigator or a designated study staff shall distribute a self-blood glucose monitoring manual to the subjects during the V1 hospitalization period and educate the subjects on the timing and method of measurement. However, blood glucose tests during outpatient visits or hospitalization may be conducted by a study staff designated by the investigator and may be substituted by a fasting blood glucose test performed prior to dosing. For details, please refer to the self-blood glucose monitoring manual.

(In cases where self-blood glucose monitoring is not performed during periods other than outpatient visits or hospitalization, the subject shall be required to document the reason, and based on the reason, it may not be considered a protocol deviation.)

19.4. Appendix 4. Injection site reaction

Assessment of local injection site adverse events will be conducted by the investigator (or a designated sub-investigator) on the following dosing days according to the study schedule: 2.2.1 Study Schedule: [redacted] 2.2.2 Study Schedule: [Part B] [redacted] Repeated Doses of PG-102 (MG12) on the dosing days (V1/D1, V3/D8*, V5/D15*, V7/D22*, V9/D29*). (* For Cohort S (optional), V3/D15, V5/D29, V7/D43, and V9/D57).

The investigator (or a designated sub-investigator) will observe and assess any adverse events occurring at the injection site after administration of the investigational product.

1) Definition of Injection site reaction (Adverse drug reaction at injection site)

Adverse drug reactions at Injection site are defined as tenderness, warmth, erythema, pruritus, hematoma, induration, nodule, bleeding, bruising, pain, edema, lipodystrophy, swelling, local irritation, redness, infection, ulceration, or necrosis.

2) Assessment Time Points

[redacted]

[Part B][redacted]: Immediately after administration of the investigational product on V1/D1, V3/D8*, V5/D15*, V7/D22*, and V9/D29*, 30 ± 10 minutes post-dose, and 1 hour ± 10 minutes post-dose. (* For Cohort S (optional), V3/D15, V5/D29, V7/D43, and V9/D57)

3) Assessment Criteria

According to the following criteria:

[Table 1] Assessment Criteria for Injection site reaction

Grade / Contents	Mild (Grade 1)[1)]	Moderate (Grade 2)[2)]	Severe (Grade 3)[3)]	Life threatening (Grade 4)
Injection site reaction	Tenderness with/without associated symptoms (e.g., warmth, erythema, pruritus)	Pain, swelling, lipodystrophy	Ulceration or necrosis; severe tissue damage; surgical intervention required	Life-threatening outcome or urgent intervention required

[1)] Subject does not interfere with normal daily life (functioning) and most likely does not require treatment

[2)] Subject can continue with the study but may require treatment

[3)] Subject is so uncomfortable that continued participation in the study is impossible and treatment or hospitalization may be required

20. List of Attachments

Attachment 1: Information on the Clinical Trial Sponsor, Clinical Trial Institution, and Investigator

Attachment 2: Subject Information Sheet and Consent Form

Attachment 3: Consent Form for Human-derived Specimen Research

Attachment 4: Consent Form for Sharing Pregnancy Information

Attachment 5: Subject compensation Agreement

Attachment 6: Self-Blood Glucose Monitoring

21. Reference

1. *「Clinical Trial Guidelines for Obesity Drugs」 Ministry of Food and Drug Safety, 2015.*
2. *Notice No. 2021-87 by the Ministry of Food and Drug Safety, 「Regulations on the Manufacture and Quality Control of Drugs」 [Effective from November 4, 2021]*
3. *Investigator's Brochure(MG12)_230519_Ver 1.0*
4. *Marx N, Husain M, Lehrke M, Verma S, Sattar N. GLP-1 Receptor Agonists for the Reduction of Atherosclerotic Cardiovascular Risk in Patients With Type 2 Diabetes. Circulation. 2022;146(24):1882-1894.*

Supplementary Note 3. Statistical Analysis Plan (Redacted)

21.1.1.1. Statistical Analysis Plan

Protocol Number	SL-MG12-P1 (Ver. 8.0) Date: 2024-10-07
Study Title	A double-blind, randomized, placebo controlled, combined single (Part A) multiple (Part B, Part C) ascending dose, phase 1 study to investigate the safety, tolerability and pharmacokinetic and pharmacodynamics following subcutaneous injections of PG-102(MG12) in healthy adult participants
Investigational Product	Investigational Product: PG-102(MG12)
Study Objective	The purpose of this study is to evaluate the safety, tolerability, pharmacokinetic, and pharmacodynamic characteristics of subcutaneous administration of PG-102 (MG12) in healthy adult participants and obese participants
Sponsor	Progen Co., Ltd.
Institution	Catholic University of Korea, Seoul St. Mary's Hospital
Principal Investigator	Seunghoon Han, Associate Professor, Department of Pharmacology, College of Medicine, Catholic University of Korea / Clinical Pharmacology Department, Seoul St. Mary's Hospital
Scope of SAP	The demographic information and related data, pharmacokinetic data, pharmacodynamic data, and safety data collected from the SL-MG12-P1 clinical trial by Progen Co., Ltd. will be subjected to statistical analysis
Statistical Analysis Summary	1. Demographic Information<br>• All subjects who have been assigned a subject number will be analyzed.<br>• Descriptive statistical analysis: Descriptive statistics will be provided for key demographic variables (age, weight, height, etc.).<br>2. Safety Evaluation<br>• Safety evaluation will be conducted for all subjects who have received at least one dose of the clinical trial medication.<br>• Adverse events will be described using MedDRA (latest version) terminology. The number of subjects experiencing adverse events and the percentage will be presented by SOC and PT. The severity, seriousness, and causal relationship to the clinical trial medication will be described.<br>• Descriptive statistics will be provided for safety evaluation variables such as laboratory tests, and subjects with clinically significant changes will be described.<br>3. Pharmacokinetic Evaluation<br>• Analysis will be performed on subjects who have completed pharmacokinetic blood sampling according to the clinical trial protocol and have measurable drug concentrations.

	• Pharmacokinetic parameters will be calculated using a non-compartmental method.<br>• Pharmacokinetic parameters will be summarized descriptively by dose group and treatment group.<br>• The linearity and dose-dependency will be assessed.<br>4. Pharmacodynamic Evaluation<br>• Continuous data will be summarized using descriptive statistics (number of observed subjects, mean, standard deviation, median, minimum, and maximum values, etc.), while categorical data will be summarized by the number and percentage of subjects in each category.
Version	3.1
Date	2024-11-26

CONFIDENTIAL

22. Signature Page

Prepared by:

Seongpil Han		
Sub-Investigator,	*Date (yyyy-mm-dd)*	*Signature*
Seoul St. Mary's Hospital		

Reviewed by:

Project Manager,	*Date (yyyy-mm-dd)*	*Signature*
Progen Co., Ltd.		

Approved by:

Seunghoon Han		
Principal Investigator,<br>Seoul St. Mary's Hospital	*Date (yyyy-mm-dd)*	*Signature*

Clinical Research Manager	*Date (yyyy-mm-dd)*	*Signature*
Progen Co., Ltd.		

TABLE OF CONTENTS

SIGNATURE PAGE 59

▣ ACRONYMS AND DEFINITIONS OF TERMS 61

1. REVISION HISTORY 62

2. PROJECT OVERVIEW 62

3. INTRODUCTION 62

3.1. Objective 62
3.2. Scope 63

4. STATISTICAL METHODS 63

4.1. Datasets 63
4.2. General Considerations 64
4.3. Subject Disposition and Demographic Information 64
4.3.1. Subject disposition 64
4.3.2. Demographic information with baseline characteristics 64
4.4. Safety 65
4.4.1. Adverse events (AEs) 65
4.4.2. Outcomes from vital signs, physical examinations, laboratory tests and ECGs 65
4.4.3. Concomitant medication 65
4.5. Pharmacokinetics 65
4.5.1. Pharmacokinetic Evaluation Variables 65
4.5.2. Principles for PK parameter determination 66
4.5.3. Basic PK evaluation 66
4.5.4. Assessment on the dose-proportionality 67
4.6. Pharmacodynamics 68
4.6.1. Pharmacodynamic parameters 68
4.6.2. Principles for PD parameter determination 70

5. LIST OF TABLES AND FIGURES 71

▣ Acronyms and Definitions of Terms

- ADR Adverse drug reaction
- AE Adverse event
- BMI Body Mass Index (kg/m^2)
- CV Coefficient of variation
- DEXA Dual Energy X-ray Absorptiometry
- ECG Electrocardiogram
- FMI Fat Mass Index
- HbA1c Hemoglobin A1c
- LLOQ Lower limit of quantification
- LMI Lean Mass Index
- OGTT Oral Glucose Tolerance Test
- PK Pharmacokinetics
- SD Standard deviation
- VAT Visceral Adipose Tissue
- WHR Waist-Hip Ratio
- max Maximum value
- mean Mean value
- median Median value
- min Minimum value

1. REVISION HISTORY

Edition No.	Release Date	Description of Changes
1.0	2024-03-08	NA (First version)
2.0	2024-05-10	Addition of Interim Analysis Timepoint in Section 4.2 (Reflecting Protocol ver 5.1)
3.0	2024-07-26	Reflecting Protocol ver 7.1
3.1	2024-11-26	Reflecting Pharmacologic Assessment (PD) Detailed Assessment Parameters

2. PROJECT OVERVIEW

Sponsor	Progen Co., Ltd.
Protocol No.	SL-MG12-P1 (Ver. 8.0) Date: 2024-10-07
Study title	A double-blind, randomized, placebo controlled, combined single (Part A) multiple (Part B, Part C) ascending dose, phase 1 study to investigate the safety, tolerability and pharmacokinetic and pharmacodynamics following subcutaneous injections of PG-102(MG12) in healthy adult participants
Institution	Catholic University of Korea, Seoul St. Mary's Hospital
Number of subjects	[redacted]<br>[Part B] PG-102(MG12) Multiple Dose<br>Up to 40 subjects: 32 subjects (based on 3 cohorts), 8 subjects (Cohort S, optional)<br>[redacted]

3. INTRODUCTION

3.1. Objective

This Statistical Analysis Plan (SAP) has been prepared for the purpose of conducting scientifically valid and reliable statistical analysis on various data obtained from ProGen's SL-MG12-P1 clinical trial, while adhering to the clinical trial protocol. Additionally, it supplements and specifies the detailed procedures and methods of statistical analysis not outlined in the protocol.

[redacted] In Part B, subjects will receive a subcutaneous injection once weekly (q1w) for five consecutive weeks, after which safety, pharmacokinetic, and pharmacodynamic characteristics will be evaluated. [redacted]

3.2. Scope

This SAP applies to the statistical analysis of the following data collected from Progen's SL-MG12-P1 clinical trial:

• Demographic information and related data

• Safety data

• Pharmacokinetic data

• Pharmacodynamic data

4. STATISTICAL METHODS

4.1. Datasets

- Demographics set (DS)
  - Includes data from all subjects who have been assigned a subject number (Assigned subjects are included regardless of their adherence to the planned study proce dures or whether they discontinued the study early)

- Safety set (SS)
  - Includes data from all subjects who have received at least one dose of the investigationa l product

- PK set (PS)
  - Includes data from subjects who have completed all planned pharmacokinetic blood samp ling and have measurable drug concentrations
  - Data from clinical trial subjects will be excluded in the following cases:
    - If the measured concentration in any sample is below the lower limit of quantification (LLOQ).
    - If any sample is missing (missing sample).
    - If a protocol violation related to inclusion/exclusion criteria occurs.
    - If a prohibited concomitant medication was taken.
    - If an investigational product with improper handling (e.g., failure to adhere to storage temperature requirements) was administered.

- PD set (PD)
  - Includes data from subjects who have received at least one dose of the investigational p roduct and have measurable pharmacodynamic assessment results

4.2. General Considerations

- Pharmacokinetic (PK) assessments may be conducted as needed during the clinical trial, maintaining blinding for both the investigator and the subject. The analysis will be performed using the PK set that satisfies the 4.1 Datasets at the evaluation time point.
- Continuous data will be presented using appropriate descriptive statistics for each item. The values for all variables will be reported up to two decimal places by default, although this may be adjusted as appropriate.
- Categorical data will be presented using frequency tables (frequency, percentage (%)). Percentages will be rounded to one decimal place.
- For all statistical analyses, only the actual measured data will be used, with no imputation for missing values.
- When performing statistical tests, parametric or non-parametric methods will be selected appropriately based on the nature of the data. In the absence of specific instructions, a significance level of 0.05 with a two-tailed test will be used.
- Statistical analysis of pharmacokinetic data and other statistical analyses will be conducted using R (Version 4.0 or higher).
- Interim analyses may be conducted to perform safety, pharmacodynamics, and pharmacokinetics analysis for each part or cohort. Interim analyses will be conducted after data lock is completed for the part or cohort that is intended to be analyzed. Only designated personnel will perform the interim analysis, and unblinding will be restricted to the designated statistical analysis personnel who are conducting the interim analysis

4.3. Subject Disposition and Demographic Information

22.1.1. 4.3.1. Subject disposition

- The number of screened subjects, the number of subjects who were excluded during screenin g, the number of enrolled subjects, the number of subjects who withdrew prematurely, and t he number of completed subjects will be summarized and presented in a table and flowchart, along with the reasons for screening failure or premature withdrawal.

22.1.2. 4.3.2. Demographic information with baseline characteristics

- Descriptive statistics (mean, standard deviation, minimum, maximum, etc.) will be provided for age, weight, height, vital signs (systolic blood pressure, diastolic blood pressure, pulse, temperature), and key electrocardiogram results (ventricular rate, PR interval, QRSD, QT, QTc) values used in the evaluation of inclusion/exclusion criteria.

- Frequency analysis will be conducted for medical history, physical examination results, and lifestyle habits. After presenting the number of subjects with a medical history, the medical history will be categorized by organ system (SOC), and the number of subjects corresponding to each organ system will be presented. Physical examination results will also be categorized by organ system, and the number of subjects corresponding to each system will be presented. For lifestyle habits, the number of subjects who have engaged in smoking, alcohol consumption, and caffeine intake will be provided.

4.4. Safety

4.4.1. Adverse events (AEs)

- Analysis Dataset: SS
- The number of subjects with treatment-emergent adverse events (TEAE), adverse drug reactions (ADR), serious adverse events (SAE), and serious adverse drug reactions (SADR) will be presented along with the incidence rate (%), and the number of occurrences.
- Adverse events will be summarized by categorizing them into local adverse events related to the administration site of the investigational product, and systemic adverse events unrelated to the administration site. All reported adverse events will be standardized using MedDRA Version 22.0 (or a later version) for SOC (System Organ Class) and PT (Preferred Term). Frequency, percentages, and the number of occurrences will be presented for each SOC and PT.

4.4.2. Outcomes from vital signs, physical examinations, laboratory tests and ECGs

- Descriptive statistics will be presented for the test items deemed necessary by the investi gator after a comprehensive review. If clinical significance is suspected based on descrip tive statistical analysis of changes before and after drug administration, appropriate statist ical tests may be performed. The choice of descriptive statistical methods for each item will follow Section 4.3.2. Summary tables and lists for each subject will be provided as appendices.

4.4.3. Concomitant medication

- In the case of concomitant medications, coding will be performed using the ATC/DDD I ndex 2022 across five levels: Level 1 (Anatomic Main Group), Level 2 (Therapeutic Ma in Group), Level 3 (Therapeutic Sub Group), Level 4 (Chemical/Therapeutic Sub Group), and Level 5 (Chemical Substance). The number of subjects and occurrences within eac h category will be presented. A summary table and list for each subject will be provide d as appendices.

4.5. Pharmacokinetics

4.5.1. Pharmacokinetic Evaluation Variables

Accumulation ratio	Accumulation ratio = $AUC_{last} / AUC_{\tau,ss}$
AUC_{last}	Area under the concentration-time curve from 0 hours to the last measurable concentration time
AUC_{inf}	Area under the concentration-time curve from 0 hours to infinity,<br>$AUC_{inf} = AUC_{last} + C_{last}/\lambda$
$AUC_{\tau,ss}$	Area under the concentration-time curve over one dosing interval after the last dose
$\%AUC_{extra}$	The percentage of AUC_{inf} extrapolated from the last dose to infinity<br>$\% AUC_{extra} = (AUC_{inf} - AUC_{last}) / AUC_{inf} * 100(\%)$

CL_{ss}/F	Oral clearance estimated from data after the last dose
C_{max}	The maximum concentration observed after the first dose
$C_{max\ ss}$	The maximum concentration observed after the last dose
$C_{min,ss}$	The minimum concentration observed during the dosing interval after the last dose
$C_{av,ss}$	The average concentration during the dosing interval after the last dose
PTF	Peak-Trough Fluctuation
T_{max}	Time to reach the maximum concentration after the first dose
$T_{max,ss}$	Time to reach the maximum concentration after the last dose
$t_{1/2}$	The half-life calculated from the data after the last dose
$V_{d,ss}/F$	Volume of distribution calculated after the last dose, adjusted for oral administration

4.5.2. **Principles for PK parameter determination**

- The pharmacokinetic evaluation variables are calculated using the non-compartmental met hod. The area under the concentration-time curve (AUC) and related variables are calcul ated using the linear trapezoidal rule, regardless of whether the concentration is increasin g or decreasing.
- Actual blood sampling times are used in the analysis. If the sampling occurs within a p redefined allowed deviation from the scheduled time, the scheduled time is used in the analysis. If there are deviations from the allowed time, the actual sampling time is used.
- When measured drug concentrations are below the LLOQ (the minimum quantifiable con centration), the value is recorded as "<LLOQ".
  For concentrations before Tmax (the time of maximum concentration), values below LL OQ are treated as zero. This is common practice in PK studies where non-quantifiable c oncentrations prior to Tmax are often not considered to have contributed to the drug's e ffect.
  For data points after Tmax that are below LLOQ, they are excluded from the analysis a s they do not provide useful information for the pharmacokinetic model.
- If a sample was not collected (marked as not applicable) or the sample was missing, th ose concentration values are excluded from the analysis as they don't contribute to the c alculation of PK parameters.

4.5.3. **Basic PK evaluation**

- Analysis Dataset: PS
- The blood concentration-time profiles for each subject will be presented as linear or log/ linear graphs, and the mean blood concentration-time curve will also be presented using the same method.
- Descriptive statistical analysis: The pharmacokinetic parameters that can be calculated wil l be summarized using descriptive statistics (median, minimum, maximum, mean, standar

d deviation).

4.5.4. **Assessment on the dose-proportionality**

- Analysis Dataset: PS
- The pharmacokinetic parameters for evaluation will include AUC_{last}, AUC_{inf}, $AUC_{\tau,ss}$, C_{max}, and $C_{max,ss}$.
- The blood concentration-time profiles will be presented as linear or log/linear graphs for each subject, and the mean blood concentration-time curve will also be shown using the same method.
- The mean values of the dose-normalized pharmacokinetic parameters for dose proportionality evaluation will be presented in a table and shown graphically.
- Dose proportionality will be assessed using a linear regression model between the logarithmically transformed dose and the logarithmically transformed pharmacokinetic parameter values, with the results presented for each parameter.
  (Additional statistical methods may be considered for dose proportionality evaluation if necessary.)

4.6. **Pharmacodynamics**

4.6.1. **Pharmacodynamic parameters**

- Analysis Dataset: PD
- [redacted]
- In Part B, Pharmacodynamic evaluation will be conducted through the following measure: 1) evaluation of weight change, 2) waist circumference and waist-hip ratio (waist-hip ratio), 3) body fat measurement (DEXA), 4) assessment of glycated hemoglobin (HbA1c) levels, and 5) fasting blood glucose and blood glucose (mg/dl) measurement after 30, 60, 90 and 120 minutes through oral glucose tolerance test (OGTT).

[redacted]

[redacted]	
[redacted]	[redacted]
	[redacted]
[redacted]	[redacted]
[redacted]	[redacted]
[redacted]	[redacted]
	[redacted]

Part B

PD parameters	
1. Chage in Body weight	Body Weight (kg)
	Body mass index (kg/m2)
2. Waist circumference and waist-hip ratio	Waist-hip ratio(WHR)
3. Body fat measurement (DEXA)	Fat Mass (kg)
	Lean Mass (kg)
	Est. VAT mass (g)
	FMI(kg/m2)
	LMI(kg/m2)
4. Glycated hemoglobin (HbA1c) levels	HbA1c (%)
5. Oral glucose tolerance test (OGTT)	Fasting glucose (mg/dl)

	Glucose at 30 min (mg/dl)
	Glucose at 60 min (mg/dl)
	Glucose at 90 min (mg/dl)
	Glucose at 120 min (mg/dl)
	Glucose area under the curve (AUC 0-2h)
Exploratory parameters	
Exploratory parameters	hsCRP (mg/dl)

4.6.2. **Principles for PD parameter determination**

- For continuous data, descriptive statistics (number of observed subjects, mean, standard d eviation, median, minimum, and maximum) will be summarized by dose group and treat ment group.
  For categorical data, the number and percentage of subjects in each category will be su mmarized and presented.

5. LIST OF TABLES AND FIGURES

The tables and figures will be presented in the following format (In accordance with the clinical trial report table of contents)

Figure #. Subject dispositions (flowchart) – Cohort #

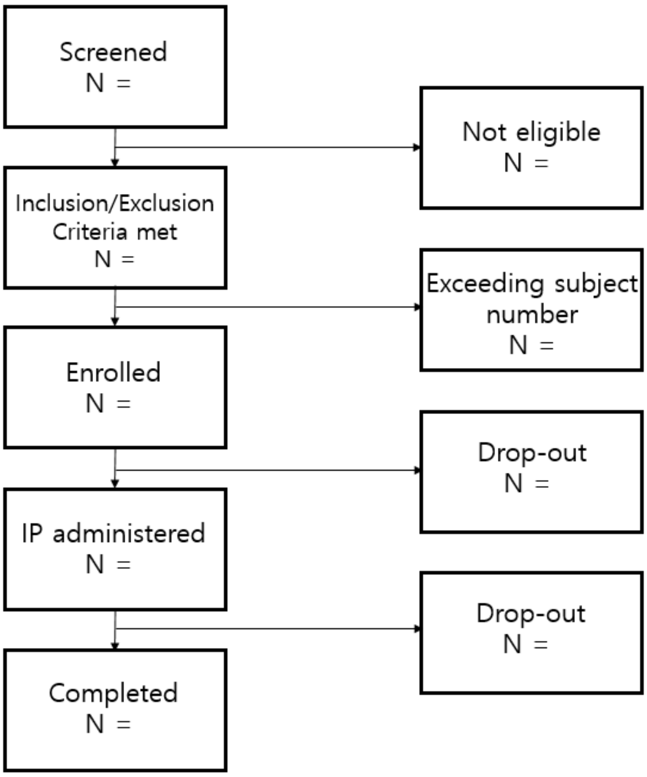

Table #. Subject disposition

Descriptions	Cohort 1	Cohort 2	Cohort #
**Screening summary**			
Number of volunteers screened	##	##	##
Number of subject enrolled	#	#	#
Number of volunteers NOT enrolled	#	#	#
**Reason for screening failure**			
Eligibility criteria	#	#	#
Consent withdrawal	#	#	#
Others	#	#	#
**Subject allocation**			
Enrolled	#	#	#
Treated	#	#	#
Non-treated	#	#	#
Completed	#	#	#
Drop-out	#	#	#
**Reason for drop-out**			
Consent withdrawal	#	#	#
Serious protocol non-compliance	#	#	#
Investigators decision related to adverse event	#	#	#
Others	#	#	#

Table #. Subject demographics & baseline characteristics summary (continuous variables)

Variables	Unit	Cohort 1<br>*N* =	Cohort 2<br>*N* =	Cohort #<br>*N* =	Total<br>N = ##
**Demographics**					
Age	years	##.## ± ##.##<br>(## - ##)	##.## ± ##.##<br>(## - ##)	##.## ± ##.##<br>(## - ##)	##.## ± ##.##<br>(## - ##)
Height	cm	###.## ± ##.##<br>(###.## - ###.##)	###.## ± ##.##<br>(###.## - ###.##)	###.## ± ##.##<br>(###.## - ###.##)	###.## ± ##.##<br>(###.## - ###.##)
Weight	kg	##.## ± ##.##<br>(##.## - ##.##)	##.## ± ##.##<br>(##.## - ##.##)	##.## ± ##.##<br>(##.## - ##.##)	##.## ± ##.##<br>(##.## - ##.##)
**Vital sign**					
Systolic blood pressure	mm Hg	###.## ± ###.##<br>(### - ###)	###.## ± ###.##<br>(### - ###)	###.## ± ###.##<br>(### - ###)	###.## ± ###.##<br>(### - ###)
Diastolic blood pressure	mm Hg	##.## ± ##.##<br>(## - ##)	##.## ± ##.##<br>(## - ##)	##.## ± ##.##<br>(## - ##)	##.## ± ##.##<br>(## - ##)
Pulse rate	bpm	##.## ± ##.##<br>(## - ##)	##.## ± ##.##<br>(## - ##)	##.## ± ##.##<br>(## - ##)	##.## ± ##.##<br>(## - ##)
Body temperature	˚C	##.## ± ##.##<br>(##.## - ##.##)	##.## ± ##.##<br>(##.## - ##.##)	##.## ± ##.##<br>(##.## - ##.##)	##.## ± ##.##<br>(##.## - ##.##)
**ECG**					
Ventricular rate	bpm	##.## ± ##.##<br>(## - ##)	##.## ± ##.##<br>(## - ##)	##.## ± ##.##<br>(## - ##)	##.## ± ##.##<br>(## - ##)
PR	msec	##.## ± ##.##	##.## ± ##.##	##.## ± ##.##	##.## ± ##.##

		(##.## - ##.##)	(##.## - ##.##)	(##.## - ##.##)	(##.## - ##.##)
QRS	msec	##.## ± ##.##<br>(##.## - ##.##)	##.## ± ##.##<br>(##.## - ##.##)	##.## ± ##.##<br>(##.## - ##.##)	##.## ± ##.##<br>(##.## - ##.##)
QT	msec	##.## ± ##.##<br>(##.## - ##.##)	##.## ± ##.##<br>(##.## - ##.##)	##.## ± ##.##<br>(##.## - ##.##)	##.## ± ##.##<br>(##.## - ##.##)
QTc	msec	##.## ± ##.##<br>(##.## - ##.##)	##.## ± ##.##<br>(##.## - ##.##)	##.## ± ##.##<br>(##.## - ##.##)	##.## ± ##.##<br>(##.## - ##.##)

All data are presented as mean ± standard deviation (range)

Table #. Subject demographics & baseline characteristics summary (discrete variables)

**Variables**	**Cohort 1**<br>***N* =**	**Cohort 2**<br>***N* =**	**Cohort 3**<br>***N* =**	**Total**<br>**N = ##**
**Medical history**				
Number of subjects no medical history	#(##.##)	#(##.##)	#(##.##)	#(##.##)
Number of subjects having clinically NOT significant medical history	#(##.##)	#(##.##)	#(##.##)	#(##.##)
**Affected organ systems in subjects having medical history**				
Integumentary system (skin/mucosa)	#(##.##)	#(##.##)	#(##.##)	#(##.##)
Head & neck	#(##.##)	#(##.##)	#(##.##)	#(##.##)
Ophthalmologic system (eye)	#(##.##)	#(##.##)	#(##.##)	#(##.##)
Ear, nose, & throat	#(##.##)	#(##.##)	#(##.##)	#(##.##)
Endocrine system	#(##.##)	#(##.##)	#(##.##)	#(##.##)

Respiratory system	#(##.##)	#(##.##)	#(##.##)	#(##.##)
Cardiovascular system	#(##.##)	#(##.##)	#(##.##)	#(##.##)
Gastrointestinal / Hepatobiliary system	#(##.##)	#(##.##)	#(##.##)	#(##.##)
Kidney / Genitourinary system	#(##.##)	#(##.##)	#(##.##)	#(##.##)
Musculoskeletal system	#(##.##)	#(##.##)	#(##.##)	#(##.##)
Neuropsychiatry	#(##.##)	#(##.##)	#(##.##)	#(##.##)
Hematology / Malignancies	#(##.##)	#(##.##)	#(##.##)	#(##.##)
Allergy / Immune system	#(##.##)	#(##.##)	#(##.##)	#(##.##)
Drug anaphylaxis (Hypersensitivity)	#(##.##)	#(##.##)	#(##.##)	#(##.##)
Others	#(##.##)	#(##.##)	#(##.##)	#(##.##)
**Physical examination**				
Number of subjects showing normal findings & clinically NOT significant abnormality(ies)	#(##.##)	#(##.##)	#(##.##)	#(##.##)
Number of subjects having clinically significant abnormality(ies)	#(##.##)	#(##.##)	#(##.##)	#(##.##)
**Affected organ systems in subjects having abnormality(ies) in physical examination**				
Integumentary system (skin/mucosa)	#(##.##)	#(##.##)	#(##.##)	#(##.##)
Head & neck	#(##.##)	#(##.##)	#(##.##)	#(##.##)
Ophthalmologic system (eye)	#(##.##)	#(##.##)	#(##.##)	#(##.##)
Ear, nose, & throat	#(##.##)	#(##.##)	#(##.##)	#(##.##)
Endocrine system	#(##.##)	#(##.##)	#(##.##)	#(##.##)

Respiratory system	#(##.##)	#(##.##)	#(##.##)	#(##.##)
Cardiovascular system	#(##.##)	#(##.##)	#(##.##)	#(##.##)
Gastrointestinal / Hepatobiliary system	#(##.##)	#(##.##)	#(##.##)	#(##.##)
Kidney / Genitourinary system	#(##.##)	#(##.##)	#(##.##)	#(##.##)
Musculoskeletal system	#(##.##)	#(##.##)	#(##.##)	#(##.##)
Neuropsychiatry	#(##.##)	#(##.##)	#(##.##)	#(##.##)
Hematology / Malignancies	#(##.##)	#(##.##)	#(##.##)	#(##.##)
Allergy / Immune system	#(##.##)	#(##.##)	#(##.##)	#(##.##)
Drug anaphylaxis (Hypersensitivity)	#(##.##)	#(##.##)	#(##.##)	#(##.##)
Others	#(##.##)	#(##.##)	#(##.##)	#(##.##)
**Lifestyle – Smoking**				
Number of smokers	#(##.##)	#(##.##)	#(##.##)	#(##.##)
Number of non-smokers	#(##.##)	#(##.##)	#(##.##)	#(##.##)
**Lifestyle - Alcohol consumption**				
Number of subjects consuming alcohol	#(##.##)	#(##.##)	#(##.##)	#(##.##)
(NOT exceeding the amount indicated in exclusion criteria)				
Number of subjects NOT consuming alcohol	#(##.##)	#(##.##)	#(##.##)	#(##.##)
**Lifestyle - Caffeine consumption**				
Number of subjects consuming caffeine	#(##.##)	#(##.##)	#(##.##)	#(##.##)

(NOT exceeding the amount indicated in exclusion criteria)				
Number of subjects NOT consuming caffeine	#(##.##)	#(##.##)	#(##.##)	#(##.##)
**ECG outcome**				
Number of subjects showing normal ECG findings & clinically NOT significant abnormality (ies)	#(##.##)	#(##.##)	#(##.##)	#(##.##)
Number of subjects showing clinically significant ECG abnormality (ies)	#(##.##)	#(##.##)	#(##.##)	#(##.##)

All data are presented as number of subject (proportion)

Table #. PK parameters for PG-102(MG12) by cohorts (N = ##)

**PK parameters**	**Cohort 1** *N* =	**Cohort 2** *N* =	**Cohort #** *N* =
AUC_{last} (ng·hr/mL)	#####.## ± #####.##	#####.## ± #####.##	#####.## ± #####.##
AUC_{inf} (ng·hr/mL)	#####.## ± #####.##	#####.## ± #####.##	#####.## ± #####.##
$AUC_{T,ss}$ (ng·hr/mL)	#####.## ± #####.##	#####.## ± #####.##	#####.## ± #####.##
% AUC_{extra} (%)	##.## ± ##.##	##.## ± ##.##	##.## ± ##.##
C_{max} (ng/mL)	####.## ± ###.##	####.## ± ###.##	####.## ± ###.##
$C_{max,ss}$ (ng/mL)	####.## ± ###.##	####.## ± ###.##	####.## ± ###.##
$C_{min,ss}$ (ng/mL)	####.## ± ###.##	####.## ± ###.##	####.## ± ###.##
$C_{av,ss}$ (ng/mL)	####.## ± ###.##	####.## ± ###.##	####.## ± ###.##
CL_{ss}/F (L/hr)	#.## ± #.##	#.## ± #.##	#.## ± #.##
t_{max} (hr)[1)]	#.## (#.## – #.##)	#.## (#.## – #.##)	#.## (#.## – #.##)
$t_{max,ss}$ (hr)[1)]	#.## (#.## – #.##)	#.## (#.## – #.##)	#.## (#.## – #.##)
$t_{1/2}$ (hr)	#.## ± #.##	#.## ± #.##	#.## ± #.##
$Vd_{,ss}/F$ (L)	##.## ± ##.##	##.## ± ##.##	##.## ± ##.##
Accumulation ratio	#.##	#.##	#.##
PTF (%)	##.##	##.##	##.##

[1)]*Median (Range)*

Table #. Dose-normalized PK parameters of PG-102(MG12) by dose

Cohort (Dose)	C_{max}/Dose	AUC_{last}/Dose	AUC_{inf}/Dose	$AUC_{\tau,ss}$/Dose	$C_{max,ss}$/Dose
Cohort 1	##.##	###.##	###.##	###.##	##.##
Cohort 2	##.##	###.##	###.##	###.##	##.##
Cohort #	##.##	###.##	###.##	###.##	##.##

Table #. Dose proportionality

Model applied: ln(Parameter) = $\beta_0 + \beta_1 \cdot \ln(\text{Dose})$				
Parameter	β_0	β_1		
		Point estimate	Margin of Proportionality	Dose Proportionality
C_{max}	#.####	#.####		Shown / NOT shown
AUC_{last}	#.####	#.####	(#.####, #.####)	Shown / NOT shown
AUC_{inf}	#.####	#.####		Shown / NOT shown
$AUC_{\tau,ss}$	#.####	#.####		Shown / NOT shown
$C_{max,ss}$	#.####	#.####		Shown / NOT shown

Table #. Summary of Adverse Events by Cohort

Variables	Cohort 1<br>N = #	Cohort 2<br>N = #	Cohort #<br>N = #	Placebo<br>N = #	Total<br>N = ##

Number of AE	# (##.#) [#]	# (##.#) [#]	# (##.#) [#]	# (##.#) [#]	# (##.#) [#]
Number of TEAE	# (##.#) [#]	# (##.#) [#]	# (##.#) [#]	# (##.#) [#]	# (##.#) [#]
Number of ADR	# (##.#) [#]	# (##.#) [#]	# (##.#) [#]	# (##.#) [#]	# (##.#) [#]
Number of SAE	# (##.#) [#]	# (##.#) [#]	# (##.#) [#]	# (##.#) [#]	# (##.#) [#]

All data presented as 'Number of subjects, (proportion), [Number of events]'

Table #. Adverse event and adverse drug reaction by severity

Severity	Cohort 1 N = #		Cohort 2 N = #		Cohort # N = #		Placebo N = #		Total N = ##	
	**AEs**	**ADRs†**	**AEs**	**ADRs†**	**AEs**	**ADRs†**	**AEs**	**ADRs†**	**AEs**	**ADRs†**
Mild	#[#]	#[#]	#[#]	#[#]	#[#]	#[#]	#[#]	#[#]	#[#]	#[#]
Moderate	#[#]	#[#]	#[#]	#[#]	#[#]	#[#]	#[#]	#[#]	#[#]	#[#]
Severe	#[#]	#[#]	#[#]	#[#]	#[#]	#[#]	#[#]	#[#]	#[#]	#[#]
**Total**	**#[#]**	**#[#]**	**#[#]**	**#[#]**	**#[#]**	**#[#]**	**#[#]**	**#[#]**	**#[#]**	**#[#]**

All data presented as 'Number of subject, [Number of events]'

†Adverse event considered to have 'definitely related', 'probably related', 'possible related', 'unlikely', and 'unknown' relationship to the investigational products

Table #. Adverse event and adverse drug reaction by system organ class (SOC) and preferred term (PT)

SOC/PT	Cohort 1 N = #		Cohort 2 N = #		Cohort # N = #		Placebo N = #		Total N = ##	
	AEs	ADRs†	AEs	ADRs†	AEs	ADRs†	AEs	ADRs†	AEs	ADRs†
**SOC #1**	#[#]	#[#]	#[#]	#[#]	#[#]	#[#]	#[#]	#[#]	#[#]	#[#]
PT #1	#[#]	#[#]	#[#]	#[#]	#[#]	#[#]	#[#]	#[#]	#[#]	#[#]
PT #2	#[#]	#[#]	#[#]	#[#]	#[#]	#[#]	#[#]	#[#]	#[#]	#[#]
**SOC #2**	#[#]	#[#]	#[#]	#[#]	#[#]	#[#]	#[#]	#[#]	#[#]	#[#]
PT #1	#[#]	#[#]	#[#]	#[#]	#[#]	#[#]	#[#]	#[#]	#[#]	#[#]
PT #2	#[#]	#[#]	#[#]	#[#]	#[#]	#[#]	#[#]	#[#]	#[#]	#[#]
**Total**	**#[#]**	**#[#]**	**#[#]**	**#[#]**	**#[#]**	**#[#]**	**#[#]**	**#[#]**	**#[#]**	**#[#]**

All data presented as 'Number of subject, [Number of events]'

†*Adverse event considered to have 'definitely related', 'probably related', 'possible related', 'unlikely', and 'unknown' relationship to the investigational products*

Table #. PD parameters for PG-102(MG12) by cohorts of Part A (N = ##)

[redacted]

…

Table #. PD parameters for PG-102(MG12) of Part A (N = ##)

[redacted]

Table #. PD parameters for PG-102(MG12) by cohorts of Part B (N = ##)

PD parameters	Baseline<br>Cohort 1<br><br>*N* =	Visit #<br>Cohort 1<br><br>*N* =	Visit #<br>Cohort 1<br><br>*N* =
**Body weight**			
Body Weight (kg)	#####.## ± #####.##	#####.## ± #####.##	#####.## ± #####.##
Body mass index (kg/m2)	#####.## ± #####.##	#####.## ± #####.##	#####.## ± #####.##
**Waist circumference and waist-hip ratio**			
Waist-hip ratio (WHR)	#####.## ± #####.##	#####.## ± #####.##	#####.## ± #####.##
**Body fat measurement (DEXA)**			
Fat Mass (kg)	#####.## ± #####.##	#####.## ± #####.##	#####.## ± #####.##
Lean Mass (kg)	#####.## ± #####.##	#####.## ± #####.##	#####.## ± #####.##
Est. VAT mass (g)	#####.## ± #####.##	#####.## ± #####.##	#####.## ± #####.##
FMI(kg/m2)	#####.## ± #####.##	#####.## ± #####.##	#####.## ± #####.##
LMI(kg/m2)	#####.## ± #####.##	#####.## ± #####.##	#####.## ± #####.##
**Glycated hemoglobin (HbA1c) levels**			
HbA1c (%)	#####.## ± #####.##	#####.## ± #####.##	#####.## ± #####.##
**Oral glucose tolerance test (OGTT)**			
Fasting glucose (mg/dl)	#####.## ± #####.##	#####.## ± #####.##	#####.## ± #####.##
Glucose at 30 min	#####.## ± #####.##	#####.## ± #####.##	#####.## ± #####.##

(mg/dl)			
Glucose at 60 min (mg/dl)	#####.## ± #####.##	#####.## ± #####.##	#####.## ± #####.##
Glucose at 90 min (mg/dl)	#####.## ± #####.##	#####.## ± #####.##	#####.## ± #####.##
Glucose at 120 min (mg/dl)	#####.## ± #####.##	#####.## ± #####.##	#####.## ± #####.##
Glucose area under the curve (AUC $_{0-2h}$)			
**Exploratory parameters**			
hsCRP (mg/dl)	#####.## ± #####.##	#####.## ± #####.##	#####.## ± #####.##
…			

Table #. PD parameters for PG-102(MG12) of Part B (N = ##)

PD parameters	*Cohort 1*<br>*N =*	*Cohort 2*<br>*N =*	*Cohort #*<br>*N =*	*Placebo*<br>*N =*
**Change in Body weight***				
Change in Body Weight (kg)	# (##.#)	# (##.#)	# (##.#)	# (##.#)
Change in Body Weight (%)	# (##.#)	# (##.#)	# (##.#)	# (##.#)
Change in Body mass index (kg/m2)	# (##.#)	# (##.#)	# (##.#)	# (##.#)
**Waist circumference and waist-hip ratio***				
Change in Waist-hip ratio(WHR)	# (##.#)	# (##.#)	# (##.#)	# (##.#)
**Change in Body fat measurement (DEXA)**				
Change in Fat Mass (kg)	# (##.#)	# (##.#)	# (##.#)	# (##.#)
Change in Lean Mass (kg)	# (##.#)	# (##.#)	# (##.#)	# (##.#)
Change in Est. VAT mass (g)	# (##.#)	# (##.#)	# (##.#)	# (##.#)
Change in FMI(kg/m2)	# (##.#)	# (##.#)	# (##.#)	# (##.#)
Change in LMI(kg/m2)	# (##.#)	# (##.#)	# (##.#)	# (##.#)
**Change in Glycated hemoglobin (HbA1c) levels**				
Change in HbA1c (%)	# (##.#)	# (##.#)	# (##.#)	# (##.#)
**Oral glucose tolerance test (OGTT)**				
Change in Fasting glucose (mg/dl)	# (##.#)	# (##.#)	# (##.#)	# (##.#)

Change in Glucose area under the curve (AUC $_{0-2h}$)	# (##.#)	# (##.#)	# (##.#)	# (##.#)
**Exploratory parameters**				
Change in hsCRP (mg/dl)	# (##.#)	# (##.#)	# (##.#)	# (##.#)
…				

*The changes in weight, waist circumference and waist-hip ratio are presented as a table of changes in the end of treatment (EOT) time compared to the baseline, and the change (absolute value and rate of change) by measurement time is presented as a graph.
