# Supplementary material for: Bispecific GLP-1/GLP-2 agonism in advanced type 2 diabetes: preclinical characterization and a randomized, double-blind, placebo-controlled phase I trial
Source: Nat Commun. 2026 Mar 24;17:4477. doi: 10.1038/s41467-026-71080-0 (PMC13187202; doi:10.1038/s41467-026-71080-0)
Supplement: Supplementary file 2 — Reporting Summary [file 41467_2026_71080_MOESM2_ESM.pdf]

nature portfolio

Corresponding author(s): Young Chul Sung

Last updated by author(s): 15/02/26

Reporting Summary

Nature Portfolio wishes to improve the reproducibility of the work that we publish. This form provides structure for consistency and transparency in reporting. For further information on Nature Portfolio policies, see our Editorial Policies and the Editorial Policy Checklist.

Statistics

For all statistical analyses, confirm that the following items are present in the figure legend, table legend, main text, or Methods section.

n/a	Confirmed	
☐	☒	The exact sample size (n) for each experimental group/condition, given as a discrete number and unit of measurement
☐	☒	A statement on whether measurements were taken from distinct samples or whether the same sample was measured repeatedly
☐	☒	The statistical test(s) used AND whether they are one- or two-sided<br>*Only common tests should be described solely by name; describe more complex techniques in the Methods section.*
☒	☐	A description of all covariates tested
☒	☐	A description of any assumptions or corrections, such as tests of normality and adjustment for multiple comparisons
☐	☒	A full description of the statistical parameters including central tendency (e.g. means) or other basic estimates (e.g. regression coefficient) AND variation (e.g. standard deviation) or associated estimates of uncertainty (e.g. confidence intervals)
☐	☒	For null hypothesis testing, the test statistic (e.g. F, t, r) with confidence intervals, effect sizes, degrees of freedom and P value noted<br>*Give P values as exact values whenever suitable.*
☒	☐	For Bayesian analysis, information on the choice of priors and Markov chain Monte Carlo settings
☒	☐	For hierarchical and complex designs, identification of the appropriate level for tests and full reporting of outcomes
☒	☐	Estimates of effect sizes (e.g. Cohen's d, Pearson's r), indicating how they were calculated

Our web collection on statistics for biologists contains articles on many of the points above.

Software and code

Policy information about availability of computer code

Data collection	No code was used.
Data analysis	Data analysis was performed using R software version 4.3.2 (R Foundation for Statistical Computing, Vienna, Austria). The code used for analysis is maintained in a private GitHub repository with two-factor authentication and can be made available upon reasonable request.

For manuscripts utilizing custom algorithms or software that are central to the research but not yet described in published literature, software must be made available to editors and reviewers. We strongly encourage code deposition in a community repository (e.g. GitHub). See the Nature Portfolio guidelines for submitting code & software for further information.

Data

Policy information about availability of data

All manuscripts must include a data availability statement. This statement should provide the following information, where applicable:

- Accession codes, unique identifiers, or web links for publicly available datasets
- A description of any restrictions on data availability
- For clinical datasets or third party data, please ensure that the statement adheres to our policy

All data supporting the findings of this study are provided within the paper and its Supplementary Information files. Source data underlying all figures and tables are provided with this paper.

Research involving human participants, their data, or biological material

Policy information about studies with human participants or human data. See also policy information about sex, gender (identity/presentation), and sexual orientation and race, ethnicity and racism.

Reporting on sex and gender	Participant sex was recorded as both numerical counts and percentages of the total study population. Enrollment was not restricted by sex or gender, and participants self-identified their sex and birth gender at the time of informed consent. The small number of female participants limited the feasibility of sex- or gender-based analyses.
Reporting on race, ethnicity, or other socially relevant groupings	The study was conducted exclusively with participants residing in the Republic of Korea.
Population characteristics	Female and male participants aged 19 to 65 years, with a BMI ranging from 25.0 kg/m² to 30.0 kg/m², inclusive. All participants consented to the use of double contraception methods throughout the study. Participants were required to be healthy, as determined by the investigator or a medically qualified designee, based on a comprehensive medical evaluation, including medical history, physical examination, laboratory tests, and ECGs.
Recruitment	Participants were recruited using advertisements, as needed. In accordance with industry standards, clinical site was selected based on feasibility assessments, ensuring prior experience in metabolic trials, regulatory compliance, and access to the target participant population. Recruitment adhered to strict inclusion and exclusion criteria outlined in the study protocol, minimizing selection bias and ensuring the intended population was appropriately represented in the trial.
Ethics oversight	The study protocol and informed consent forms were reviewed and approved by the Institutional Review Board at the Catholic University Seoul St. Mary's Hospital, Korea.

Note that full information on the approval of the study protocol must also be provided in the manuscript.

Field-specific reporting

Please select the one below that is the best fit for your research. If you are not sure, read the appropriate sections before making your selection.

☒ Life sciences ☐ Behavioural & social sciences ☐ Ecological, evolutionary & environmental sciences

For a reference copy of the document with all sections, see nature.com/documents/nr-reporting-summary-flat.pdf

Life sciences study design

All studies must disclose on these points even when the disclosure is negative.

Sample size	Clinical study: The sample size was not determined based on statistical inferences. In the Multiple Ascending Dose (MAD) phase, each cohort consisted of 8 participants, randomized in an 6:2 ratio to receive either PG-102 or a placebo. The primary objectives were to assess safety, tolerability, pharmacokinetics, and pharmacodynamics.
Data exclusions	Clinical study: For the pharmacokinetic (PK) analysis, participants with incomplete data were excluded to maintain the integrity of the results.
Replication	Clinical study: Clinical samples were analyzed for each analyte across all designated time points to ensure consistency and reliability of the data.
Randomization	Clinical study: Participants in each cohort were randomly assigned to either the treatment group or the placebo group.
Blinding	Clinical study: This study was conducted as a double-blind trial. Both participants and investigators were blinded to the treatment or placebo assignments during dosing, data collection, and analysis.

Reporting for specific materials, systems and methods

We require information from authors about some types of materials, experimental systems and methods used in many studies. Here, indicate whether each material, system or method listed is relevant to your study. If you are not sure if a list item applies to your research, read the appropriate section before selecting a response.

Materials & experimental systems

n/a	Involved in the study	
☐	☒	Antibodies
☐	☒	Eukaryotic cell lines
☒	☐	Palaeontology and archaeology
☐	☒	Animals and other organisms
☐	☒	Clinical data
☒	☐	Dual use research of concern
☒	☐	Plants

Methods

n/a	Involved in the study	
☒	☐	ChIP-seq
☐	☒	Flow cytometry
☒	☐	MRI-based neuroimaging

Antibodies

Antibodies used

1) Clinical pharmacokinetic (PK) assays were conducted using the following monoclonal antibodies: GLP-1 Monoclonal Antibody, Biotin (Invitrogen, Catalog No: ABS 033-10B-005) and GLP-2 Antibody (AbFRONTIER, Catalog No: K190747). Clinical PK was assessed using ELISA.
2) Preclinical immunofluorescence staining of pancreatic tissues was performed using the following antibodies: anti-insulin polyclonal antibody (Abcam, Catalog No: ab181547), anti-glucagon monoclonal antibody (Abcam, Catalog No: ab10988), and anti-Ki-67 polyclonal antibody (Abcam, Catalog No: ab15580). For immunohistochemistry, anti-insulin monoclonal antibody (Santa Cruz, Catalog No: sc-8033) was used.
3) Glucose-stimulated insulin secretion from isolated islets was measured using an ELISA kit (ALPCO). For flow cytometry analysis, the following antibodies were used: anti-GLP-1 receptor (Alomone Labs, Catalog No: AGR-021-F) and anti-GLP-2 receptor (Alomone Labs, Catalog No: AGR-022).

Validation

1) Pharmacokinetic (PK) validation was performed at a GCLP-certified institution, in accordance with the Bioanalytical Method Validation Guidance for Industry (FDA, 2018) and the Guideline on Bioanalytical Method Validation (EMA, 2011).
2) All antibodies used in preclinical studies were commercially sourced, and experiments were conducted following the manufacturer's protocols and product specifications.

Eukaryotic cell lines

Policy information about cell lines and Sex and Gender in Research

Cell line source(s)

1) The parental cell line utilized in this study was the commercially available CHOZN® ZFN-modified GS-/- CHO cell line. This line was generated through transfection of the CHOZN® ZFN-modified GS-/- CHO cell line with the pCGS3-D23A expression vector.
2) The 3T3-L1 adipocytes and L6-GLUT4myc myoblasts, employed in the in vitro glucose uptake study, are also commercially available.

Authentication

The cell lines were not independently authenticated.

Mycoplasma contamination

All cell lines tested negative for mycoplasma contamination (MCB/WCB: no detection of mycoplasma).

Commonly misidentified lines
(See ICLAC register)

None

Animals and other research organisms

Policy information about studies involving animals; ARRIVE guidelines recommended for reporting animal research, and Sex and Gender in Research

Laboratory animals

Mice were housed in AAALAC International-accredited facilities and maintained in accordance with IACUC oversight. Mice (Mus musculus) were housed in individual static or ventilated caging (IVC) systems (LB-MC-001) with irradiated corncob bedding (SAFE SELECT, Safe Lab). Lighting in animal holding rooms followed a 12:12-hour light-dark cycle, with ambient temperature and humidity ranges maintained at 68–79°F and 40–60%, respectively. Animals were provided ad libitum access to irradiated pelleted feed (Envigo Teklad Global 18% Protein Rodent Diet, irradiated T.2018C) and reverse-osmosis (RO) chlorinated water (2–3 ppm) via an automatic watering system. Male db/db mice (BKS-Leprdb/dbJOriRj) and control db/m mice were purchased from Janvier-Labs (Le Genest-Saint-Isle, France).

Wild animals

No wild animals were used in these studies.

Reporting on sex

All studies were performed in male mice to minimize variability and ensure reproducibility. Female mice undergo estrous cycle with hormonal fluctuations that affect body weight, adiposity, insulin sensitivity, and energy metabolism, leading to higher variability and requiring larger sample sizes. Moreover, male mice develop diet-induced obesity and insulin resistance more rapidly and consistently, making them the preferred choice for robust metabolic disease models and for direct comparison with prior studies.

Field-collected samples

No field-collected samples were utilized in these studies.

Ethics oversight

All studies involving non-human animals complied with relevant ethical regulations and were approved by the GI Biome Institutional Animal Care and Use Committee, in accordance with the Guide for Experimental Animal Research of Laboratory Animals (Approval Number: GIB-23-02-007).

Note that full information on the approval of the study protocol must also be provided in the manuscript.

Clinical data

Policy information about clinical studies

All manuscripts should comply with the ICMJE guidelines for publication of clinical research and a completed CONSORT checklist must be included with all submissions.

Clinical trial registration

NCT06309667

Study protocol

The multiple ascending dose (MAD) study commenced in March 2024, and this paper reports data from the MAD cohorts. The clinical study protocol is provided in the Supplementary Information (Supplementary Note 2).

Data collection

The clinical study was conducted at the Catholic University Seoul St. Mary Hospital in the Republic of Korea. The MAD study began in March 2024 and completed in November 2024. Data collection was performed by trained study personnel adhering to a detailed study protocol under the sponsor's supervision, ensuring compliance with Good Clinical Practices (GCP) and adherence to the protocol. Participants in the MAD cohorts were randomized in an 6:2 ratio (8 participants per cohort) to receive either PG-102 or a placebo.

Outcomes

Assessment methods for all endpoints were standardized and predefined in the approved study protocol, including the timing of each evaluation. Study site staff received training in protocol implementation and assessment procedures to maintain consistency.
Primary endpoints comprised the incidence of treatment-emergent adverse events (TEAEs), changes in laboratory safety parameters, vital signs, and 12-lead electrocardiogram results.
Secondary endpoints included pharmacokinetic (PK) parameters of PG-102, including Cmax, Tmax, and AUC.
Exploratory endpoints evaluated pharmacodynamic (PD) parameters such as oral glucose tolerance test (OGTT) results, HbA1c levels, and changes in body weight (BW), waist circumference, and body mass index (BMI).

Plants

Seed stocks

Report on the source of all seed stocks or other plant material used. If applicable, state the seed stock centre and catalogue number. If plant specimens were collected from the field, describe the collection location, date and sampling procedures.

Novel plant genotypes

Describe the methods by which all novel plant genotypes were produced. This includes those generated by transgenic approaches, gene editing, chemical/radiation-based mutagenesis and hybridization. For transgenic lines, describe the transformation method, the number of independent lines analyzed and the generation upon which experiments were performed. For gene-edited lines, describe the editor used, the endogenous sequence targeted for editing, the targeting guide RNA sequence (if applicable) and how the editor was applied.

Authentication

Describe any authentication procedures for each seed stock used or novel genotype generated. Describe any experiments used to assess the effect of a mutation and, where applicable, how potential secondary effects (e.g. second site T-DNA insertions, mosiacism, off-target gene editing) were examined.

Flow Cytometry

Plots

Confirm that:

- [ ] The axis labels state the marker and fluorochrome used (e.g. CD4-FITC).
- [ ] The axis scales are clearly visible. Include numbers along axes only for bottom left plot of group (a 'group' is an analysis of identical markers).
- [ ] All plots are contour plots with outliers or pseudocolor plots.
- [ ] A numerical value for number of cells or percentage (with statistics) is provided.

Methodology

Sample preparation

Cells were seeded into 96-well plates at a density of 5 × 10^5 cells per well, and staining procedures were performed in-plate.Cells were washed twice with FACS buffer (centrifugation: 1800 rpm, 4 °C, 3 min). Fc blocking was conducted using TruStain FcX™ (anti-mouse CD16/32; 2 µL/well) for 10 min on ice. Without additional washing, cells were incubated with anti-GLP-1R, anti-GLP-2R, or isotype control antibodies (1:50 dilution) at 4 °C for 30 min. Cells were then washed twice with FACS buffer. For GLP-2R staining, a secondary antibody (goat anti-rabbit IgG, APC-conjugated; 1:500 dilution) was added and incubated at 4 °C for 30 min. After two final washes with FACS buffer, samples were immediately analyzed by flow cytometry.

Instrument

Flow cytometric analysis was performed using a CytoFLEX LX flow cytometer (Beckman Coulter, Brea, USA). Cell counting was performed with Countess 3 (Invitrogen, CA, USA), and centrifugation steps utilized a Centrifuge 5910R (Eppendorf, Hamburg, Germany).

Software	Data acquisition and analysis were carried out using FlowJo v10.8.1 (BD Biosciences).
Cell population abundance	A total of 20,000 events were acquired per sample. The abundance of relevant cell populations was determined based on live/dead staining and receptor-specific marker expression. The proportion of live cells exceeded 97% in all samples, confirming high purity.
Gating strategy	The following gating strategy was applied:Total cells → Single cells → Live/Dead → GLP-1R$^+$ and GLP-2R$^+$ populations. Forward and side scatter (FSC/SSC) were used to exclude debris and doublets. Gates for positive and negative staining were established using isotype control antibodies. Live/dead gating was performed with viability dye exclusion, and all gates were consistently applied across biological replicates. of note, quantitative data (MFI) were summarized as mean ± SD from flow cytometric analyses; representative raw flow plots were not included, as Extended Data Fig. 5 presents aggregated MFI data only.

☐ Tick this box to confirm that a figure exemplifying the gating strategy is provided in the Supplementary Information.
